# Quantitative reconstitution of yeast RNA processing bodies

**DOI:** 10.1073/pnas.2214064120

**Published:** 2023-03-27

**Authors:** Simon L. Currie, Wenmin Xing, Denise Muhlrad, Carolyn J. Decker, Roy Parker, Michael K. Rosen

**Affiliations:** ^a^HHMI, UT Southwestern Medical Center, Dallas TX 75390; ^b^Department of Biophysics, UT Southwestern Medical Center, Dallas, TX 75390; ^c^HHMI, University of Colorado, Boulder, CO 80309; ^d^Department of Biochemistry, University of Colorado, Boulder, CO 80309

**Keywords:** biomolecular condensates, phase separation, RNA, protein, P bodies

## Abstract

Many biomolecular condensates appear to form through liquid–liquid phase separation (LLPS). Individual condensate components can often undergo LLPS in vitro, capturing some features of the native structures. However, natural condensates contain dozens of components with different concentrations, dynamics, and contributions to compartment formation. Most biochemical reconstitutions of condensates have not benefited from quantitative knowledge of these cellular features nor attempted to capture natural complexity. Here, we build on prior quantitative cellular studies to reconstitute yeast RNA processing bodies (P bodies) from purified components. Individually, five of the seven highly concentrated P-body proteins form homotypic condensates at cellular protein and salt concentrations, using both structured domains and intrinsically disordered regions. Combining the seven proteins together at their cellular concentrations with RNA yields phase-separated droplets with partition coefficients and dynamics of most proteins in reasonable agreement with cellular values. RNA delays the maturation of proteins within and promotes the reversibility of, P bodies. Our ability to quantitatively recapitulate the composition and dynamics of a condensate from its most concentrated components suggests that simple interactions between these components carry much of the information that defines the physical properties of the cellular structure.

## Significance

Biomolecular condensates concentrate related biomolecules within cells. Biochemical reconstitutions using one to a few molecules indicate that many condensates are formed through liquid–liquid phase separation. However, it is unclear to what extent the principles gleaned from simplified reconstitutions apply to complex cellular condensates that are enriched in tens to hundreds of different molecules. Here, we used the seven most highly concentrated proteins in yeast RNA processing bodies (P bodies) and RNA to reconstitute these archetypal condensates. Combining proteins and RNA at their cellular concentrations generated in vitro P bodies with protein partitioning and dynamics that are quantitatively consistent with cellular measurements. Our findings suggest that much of the information specifying condensate properties is carried by interactions between the most abundant components.

Biomolecular condensates are cellular compartments that lack an enclosing membrane barrier, yet are compositionally distinct from the surrounding environment (1, 2). Many condensates have liquid-like behaviors in cells, for example, fusion and rounding due to surface tension, and rapid dynamics of key molecules. Some also have been shown to form through liquid–liquid phase transitions, based on sharp appearance/disappearance with changes in physical parameters (e.g., concentration, environmental conditions), dynamic behaviors, and in vitro–in vivo correlations (3–11). Many studies have demonstrated that condensates can be formed in vitro using simple components from their natural counterparts—through phase separation of only a single or a few types of molecules. Such studies have demonstrated that relatively few components are required to form condensates, at least in vitro (5–7, 12–20). Further, retention of mechanisms that regulate condensate formation and/or of condensate functions indicates that in vitro condensates can retain key features of the more complex cellular structures (5, 6, 18–29).

However, proteomic and sequencing studies indicate that cellular condensates consist of tens to hundreds of different molecules, including proteins and nucleic acids (30–34). Compositional complexity is coupled to more heterogeneous mechanisms of formation than observed for individual molecules. For example, for a given set of environmental conditions, a single protein will phase separate at a specific concentration producing droplets of a defined concentration. However, in multicomponent systems, both the phase separation boundary and the composition of the condensate depend on the specific nature of the interactions between the various components and thus on their relative and absolute concentrations (8, 9, 35, 36). Some components, by virtue of their interactions (valency, interaction partners, binding affinities), play more important roles in defining these parameters than others. Due to cooperativities between molecular interactions in multicomponent systems, even when many/most components are known, it is not currently possible to quantitatively predict the compositions and physical properties of condensates from knowledge of their molecular parts and binary interactions thereof. As a step toward such a quantitative view, recent systematic surveys of cellular condensates to understand the concentrations of components and/or their requirements for condensate formation, have been reported (10, 37). Complex reconstitutions, containing all of the molecule types that are highly enriched in cellular condensates, provide an additional approach to a fuller understanding of the mechanisms that control the formation, properties, and functions of natural condensates.

RNA processing bodies (P bodies) are archetypal biomolecular condensates consisting of RNA and proteins implicated in RNA metabolism, including enzymes with RNA-helicase (Dhh1), -decapping (Dcp2), and -exonucleolytic (Xrn1) activities (38–43). P bodies are thought to serve as sites of enhanced RNA degradation and/or of RNA/protein storage (30, 43–46). Previous biochemical reconstitutions have found that several different combinations of P-body proteins are sufficient to form condensates in vitro including Dcp2, Edc3, Dcp1, and RNA (13, 21, 47); Dhh1, Pat1, and RNA (14, 48); and Dcp1, Dcp2, Lsm1-7, Pat1, and RNA (49). In some instances, RNA reduced the saturation concentration (C_sat_) of P-body proteins (47), whereas in other cases, RNA had no impact (21). Genetic studies indicate that individual P-body proteins can either promote or repress P-body formation, though no single protein is absolutely required (50, 51). Pat1, Edc3, and to a lesser extent Dhh1 promote yeast P-body formation, likely through engaging in multivalent protein–protein and protein–RNA interactions (*SI Appendix*, Table S1 and Fig. S1) (14, 50, 52–54). In contrast, Dcp1, Xrn1, and Lsm1 repress P-body formation, as deletion of any of these components results in larger P-bodies and/or higher partitioning of other components (37, 50, 55). This repression may occur via their functions in RNA degradation, as RNA may contribute to P-body formation (50, 56), although RNA does not appear to be required for the maintenance of mammalian P bodies (57). Dcp2 represses P-body formation in cells growing in the mid-log phase by limiting the available deadenylated mRNAs required for P-body assembly but promotes P-body formation under glucose starvation, or when decapping is blocked by deletion of Dcp1 (*dcp1∆*), conditions that cause accumulation of large pools of deadenylated mRNAs independent of Dcp2 (36). This demonstrates how a single component of a condensate can have multiple inputs into condensate formation that are context dependent. These biochemical and genetic investigations suggest that P bodies are formed through multiple, partially redundant interactions (51, 58).

**Fig. 1.**
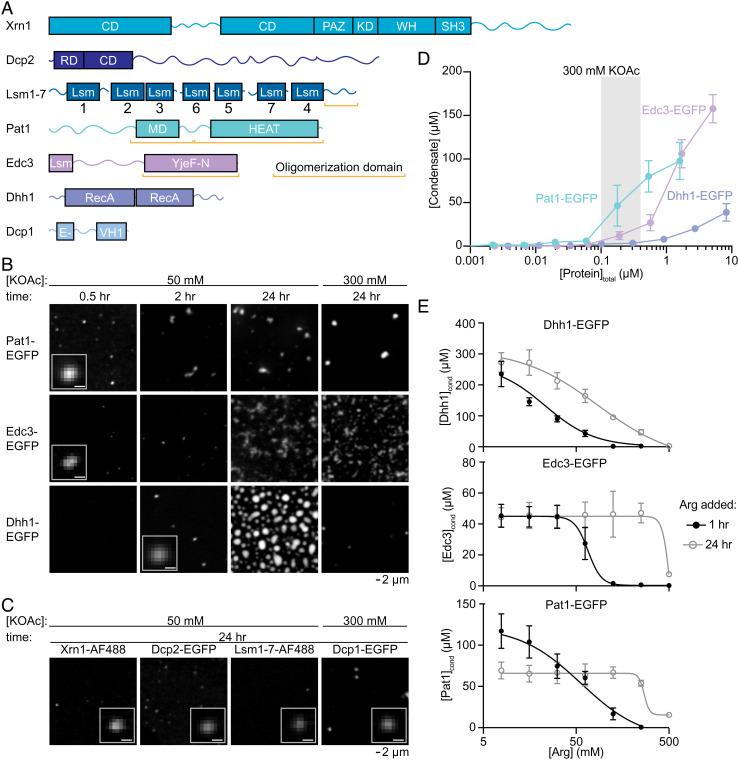
Individual P-body proteins phase separate. (*A*) Domain structure of P-body proteins. Boxes indicate structured domains, lines represent IDRs, and orange brackets indicate previously known oligomerization domains. (*B*) Fluorescent micrographs for individual P-body EGFP-fusion proteins at pH 7 with 50 mM, *Left*, and 300 mM, *Right*, KOAc. (Scale bar for *Inset* is 0.5 μm.) Pat1 is at 0.5 μM and Edc3 and Dhh1 are at 1 μM total concentration. Proteins labeled with EGFP. (*C*) Fluorescent micrographs for Dcp2, Lsm1-7, and Dcp1 at 1 μM and Xrn1 at 0.4 μM total concentration. Dcp1 and Dcp2 are labeled with EGFP, Lsm1-7, and Xrn1 are labeled with Alexa Fluor 488 (AF488). (*D*) Condensate concentration vs. total protein concentration for Pat1, Edc3, and Dhh1 at pH 7 and 300 mM KOAc. Gray rectangle represents estimated cellular protein concentrations. (*E*) Condensate protein concentration versus arginine added for Dhh1 (*Left*), Edc3 (*Middle*), and Pat1 (*Left*). Black closed circles and gray open circles correspond to arginine added 1 h and 24 h after condensate initiation, respectively. See also *SI Appendix*, Figs. S1–S6.

Understanding the thermodynamics of condensate formation, including which interactions strongly contribute to it, requires knowledge about both the identity and the abundance of molecules within a given condensate (59). We recently performed a systematic analysis of *Saccharomyces cerevisiae* P bodies and identified eight proteins (of 31 P-body components examined) that are highly concentrated and have large partition coefficients (PC, concentration ratio of condensate to bulk cytoplasm) in vivo (Fig. 1*A* and *SI Appendix*, Fig. S1) (37). This group included all of the aforementioned proteins that contribute to P-body formation in genetic experiments and Upf1. All other proteins, except Sbp1, exhibited at least twofold lower concentrations in P bodies, and all had at least twofold smaller PC values (compared to the lowest concentration and PC values of the other group) (37). The concentrations and dynamic properties of many of the proteins were qualitatively correlated with the P-body connectivity network, where network centrality was associated with higher concentration and lower dynamics (37).

Here, we sought to understand whether the most concentrated proteins in P bodies could produce condensates with RNA in vitro that have properties similar to their cellular counterparts. We find that five proteins (Pat1, Dhh1, Edc3, Dcp1, and Xrn1) form homotypic condensates at cellular protein and salt concentrations. Molecular dissections indicate that condensate formation by these proteins occurs through interactions of both folded domains and disordered regions. Acidic pH, mimicking the dormancy state during which large yeast P-bodies form (60, 61), promotes condensate formation by enhancing both homotypic phase separation and the affinity of P-body proteins for RNA. Combining all proteins with RNA at their cellular concentrations, and under cellular environmental conditions, produces heterotypic condensates whose PC values and dynamics are quantitively similar to those of cellular P bodies. Thus, the capacity to generate these cellular structures may reside critically in the most concentrated components (consistent with prior genetic analyses). Comparisons of the single-component and eight-component condensates suggest that competing homotypic and heterotypic interactions are integrated within the multicomponent droplets. Our quantitative reconstitution is a valuable system for future examinations of the formation and biochemical function(s) of P bodies and provides a general framework for understanding the formation of multicomponent condensates.

## Results

### Individual P-Body Proteins Phase Separate under Physiologic Conditions.

We examined seven proteins that are highly concentrated in *S. cerevisiae* P bodies: Dcp1, Dcp2, Dhh1, Edc3, Lsm1-7, Pat1, and Xrn1 (Fig. 1*A*) (37). These seven core P-body proteins possess many features associated with condensate formation including multivalent molecular interactions, intrinsically disordered regions (IDRs), and oligomerization domains/regions (Fig. 1*A* and *SI Appendix*, Figs. S1–S3). Full-length Dcp1, Dcp2, Dhh1, Edc3, and Pat1 were expressed individually in bacteria with N-terminal maltose binding protein (MBP) and C-terminal His_6_ tags. Double-affinity purification yielded full-length reagents (*SI Appendix*, Fig. S4*A*). To increase solubility, we retained the MBP fusion throughout the purification and used Tobacco Etch Virus (TEV) protease cleavage of both tags to initiate condensate formation (*SI Appendix*, Fig. S4*B* and *Materials and Methods*). P-body proteins with Enhanced Green Fluorescent Protein (EGFP) inserted immediately before the C-terminal TEV cleavage site were also expressed and purified**.** We used established expression plasmids and purification protocols for the Lsm1-7 complex and Xrn1 (38, 62, 63). Alexa Fluor 488-conjugated versions of Lsm1-7 and Xrn1 were also purified.

We initially examined individual P-body proteins labeled with EGFP or Alexa Fluor 488 (*Materials and Methods*) at 0.4 to 1 µM concentration and pH 7, similar to the cytoplasm of unstressed yeast in log phase growth (61). Each protein formed micron-sized condensates (Fig. 1 *B* and *C*). Pat1, Edc3, and Dhh1 rapidly formed small spherical condensates with partition coefficients (ratio of droplet to bulk concentration) greater than 40 in 50 mM KOAc. Comparatively, Dcp1, Dcp2, Lsm1-7, and Xrn1 slowly formed condensates with lower partition coefficients (less than 5). Physiologically relevant salt concentrations [300 mM KOAc: (64)] eliminated Dcp2, Lsm1-7, and Xrn1 condensates, reduced the size and number of Dhh1 condensates, had minimal impact on Edc3 and Pat1 condensates, and slightly enhanced Dcp1 condensates. EGFP alone does not form condensates (*SI Appendix*, Fig. S4*C*), suggesting that P-body proteins are driving condensate formation. Protein concentrations (0.4 to 1 µM) in these assays were roughly 2- to 10-fold higher than their estimated cellular concentrations (Fig. 1 *B* and *C* and *SI Appendix*, Fig. S5) (65). However, condensates form at pH 7 with physiological protein and salt concentrations for Pat1, Edc3, and Dhh1 (Fig. 1*D*), consistent with these proteins playing important roles in cellular P-body formation (14, 50, 52, 53).

Although all proteins initially formed small spherical homotypic condensates, different proteins transitioned to distinct morphologies over time. Dhh1 condensates grew into larger spherical objects, whereas Pat1 and Edc3 formed aspherical networks of condensates (Fig. 1*B*). One interpretation of this result is that homotypic condensates of Edc3 and Pat1 rapidly mature into structures that cannot round, while Dhh1 condensates remain more dynamic with surface tension that produces a spherical shape (see below). To probe the maturation of condensates, we examined whether they become more resistant to dissolution over time. We screened for molecules that disrupt homotypic condensate formation. Dhh1 condensates were disrupted by salt (NaCl), glycerol, and arginine. In contrast, Edc3 and Pat1 were only disrupted by arginine (*SI Appendix*, Fig. S6*A*). The concentration of arginine required to dissolve homotypic condensates varied by protein, yet arginine more potently disrupted each of the homotypic condensates when aged for 1 h as compared to 24 h (Fig. 1*E* and *SI Appendix*, Fig. S6*B*). These data suggest that in the absence of other factors (e.g., RNA, see Fig. 5 and *Discussion*), homotypic P-body protein condensates convert into a less reversible state.

### Acidic pH Enhances Homotypic Condensate Formation and Protein–RNA Interactions.

**Fig. 2.**
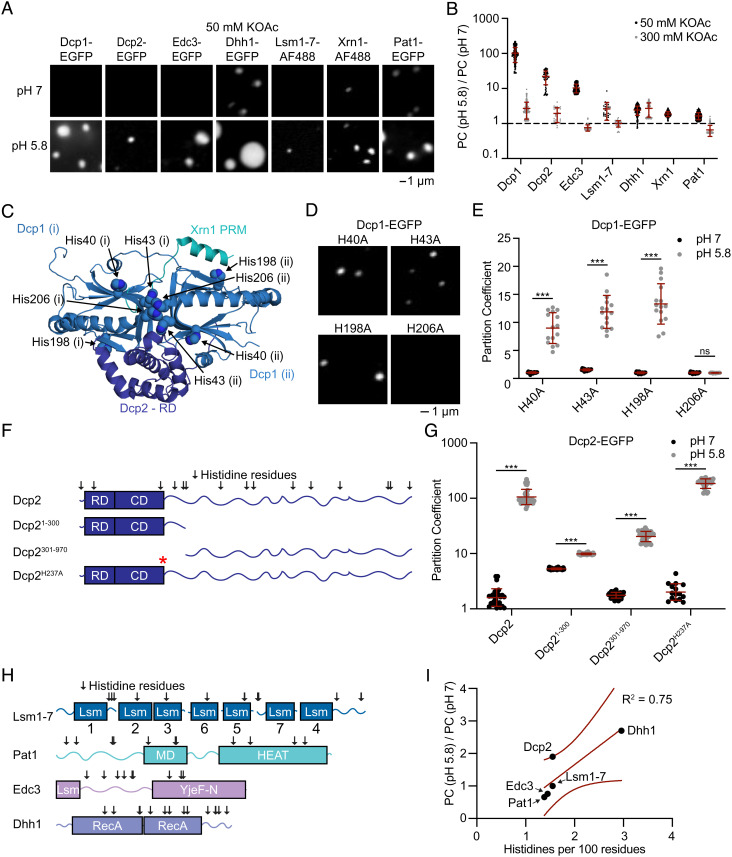
Acidic pH promotes homotypic phase separation. (*A*) Micrographs for individual P-body proteins at pH 7 (*Top*) or 5.8 (*Bottom*) and 50 mM KOAc. (*B*) Ratio of partition coefficients for individual P-body proteins at pH 5.8 and pH 7. (*C*) Crystal structure of the Dcp1 dimer (67) annotated with mutated histidine residues. Interactions with Xrn1 (68) and Dcp2 (69) are modeled. (*i*) and (*ii*) are distinct Dcp1 molecules. (*D*) Micrographs of Dcp1 histidine mutants at pH 5.8 and 300 mM KOAc. (*E*) Partition coefficients of Dcp1 histidine mutants with 300 mM KOAc, pH 7 (black) and pH 5.8 (gray). (*F*) Schematic of Dcp2 truncations and point mutations tested. Arrows above Dcp2 indicate histidine residues. Red asterisk indicates His237. (*G*) Partition coefficients for Dcp2 truncations and mutations with 300 mM KOAc, pH 7 (black) and pH 5.8 (gray). (*H*) Location of histidine residues in Lsm1-7, Pat1, Edc3, and Dhh1. (*I*) Ratio of partition coefficients (pH 5.8/pH 7) with 300 mM KOAc versus the frequency of histidine residues (histidine residues per 100 amino acids) for P-body proteins. See also *SI Appendix*, Figs. S7–S9.

P bodies form in response to cellular stresses that elicit cell cycle arrest and entry into a dormant state (56). This transition changes the cellular environment including acidifying the cytosol (60, 61). We therefore wondered whether P-body proteins might respond to pH changes. Indeed, all P-body proteins exhibited more numerous condensates and higher PCs at pH 5.8 in 50 mM KOAc (Fig. 2*A* and *SI Appendix*, Fig. S7). The magnitude of pH-sensitivity of PC values ranged from 100-fold for Dcp1 to twofold for Pat1 (Fig. 2*B* and *SI Appendix*, Fig. S7 *B* and *C*). Then, 300 mM KOAc reduced the pH-sensitivity of PCs for most proteins, suggesting that ionizable groups may be responsible for sensing pH changes. We hypothesized that histidine residues may be important in pH-sensitive condensate formation because the pH value tested bracket the pK_A_ of the imidazole side chain (66). Analysis of a previously published structure of Dcp1 (67) suggested that H206 might be important for Dcp1 condensate formation as it is centrally located within the homodimer interface for both Dcp1 molecules (Fig. 2*C*). Indeed, mutation of H206 to alanine specifically inhibits oligomerization and homotypic condensate formation by Dcp1 at pH 5.8, whereas mutation of other histidine residues had less impact (Fig. 2 *D* and *E* and *SI Appendix*, Fig. S8). In contrast, truncations and point mutants in Dcp2 (described further below) indicated that elements distributed in both the structured N-terminal domains and in the C-terminal IDR of the protein contribute to pH-sensitive condensate formation (Fig. 2 *F* and *G* and *SI Appendix*, Fig. S9*A*). Histidine frequency correlates with pH-sensitive partitioning of P-body proteins other than Dcp1 (Fig. 2 *H* and *I* and *SI Appendix*, Fig. S9*B*), whereas proteins’ isoelectric points do not correlate with pH-sensitive partitioning (*SI Appendix*, Fig. S9*C*). This suggests that similar to Dcp2, multiple distributed histidine residues likely contribute to pH-sensitive liquid–liquid phase separation (LLPS) behaviors of other P-body proteins as well. Together, the data suggest that the protonation of histidine residues at acidic pH promotes the oligomerization and homotypic condensate formation of P-body proteins.

**Fig. 3.**
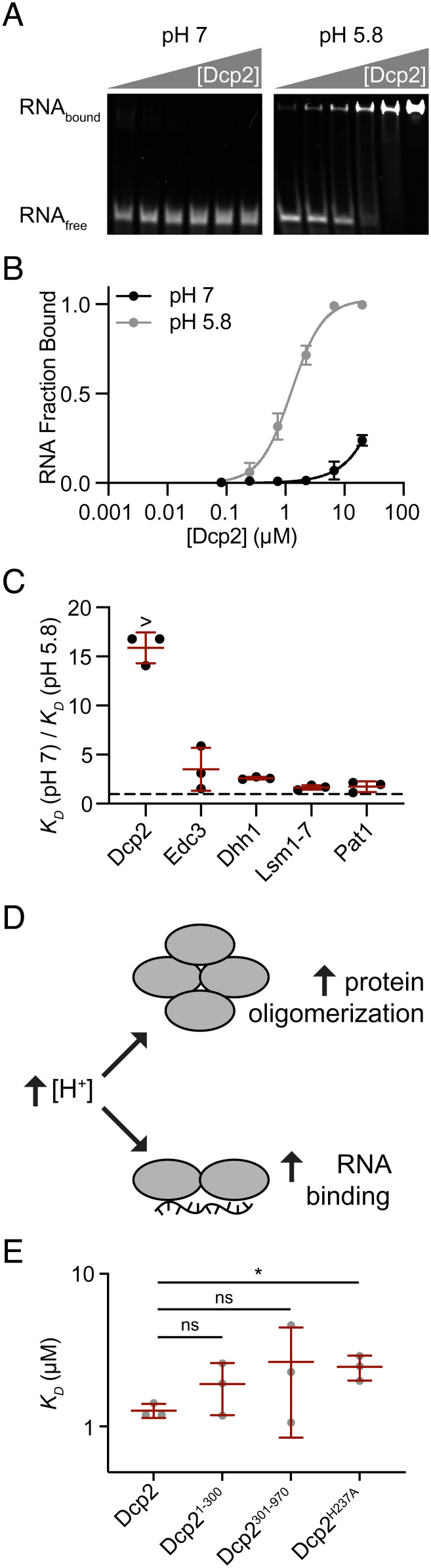
Acidic pH promotes P-body proteins binding to RNA. (*A*) EMSA gels for Dcp2 binding to RPL41A RNA with 300 mM KOAc, pH 7 (*Left*), and 5.8 (*Right*). (*B*) Quantitation of Dcp2 binding to RPL41A RNA at pH 7 (black) and pH 5.8 (gray). (*C*) Ratio of equilibrium dissociation constants, *K_D_*, for individual P-body proteins at pH 7 and 5.8. Ratios for Dcp2 are lower limits as RNA binding at pH 7 was too weak to accurately quantify. (*D*) Acidic pH promotes protein oligomerization and homotypic LLPS (*Top*) and tighter binding to RNA (*Bottom*). (*E*) *K_D_* values for Dcp2 truncations and point mutants binding to RPL41A RNA (pH 5.8, 300 mM KOAc). See also *SI Appendix*, Tables S2 and S3 and Figs. S10 and S11.

Having established an importance for pH in homotypic interactions, we queried whether pH might also regulate protein–RNA interactions. Dimerization can enhance RNA–protein interactions through avidity effects (70). Therefore, we reasoned that pH-enhanced oligomerization of P-body proteins might increase their affinity for RNA. We used Electrophoretic Mobility Shift Assays (EMSAs) to measure the equilibrium dissociation constants (*K_D_*) for binding of each protein to RNA at pH 7 and pH 5.8 (Fig. 3*A* and *SI Appendix*, Fig. S10). We used a 60-nucleotide RNA portion of RPL41A mRNA, a known resident of yeast P bodies (*SI Appendix*, Fig. S11) (71). Acidic pH increased the RNA-binding affinity of all proteins, except for Dcp1 which did not appreciably bind to RNA at either pH (*SI Appendix*, Table S2 and Fig. S10 and Fig. 3 *B* and *C*). Dcp1 not binding to RNA is in agreement with the lack of direct contact between Dcp1 and RNA in a previous Dcp1–Dcp2–RNA structure (72). The Dcp2–RNA interaction was greater than 16-fold tighter at pH 5.8 versus pH 7.0, whereas the other proteins showed more modest pH-dependent enhancements (two- to five-fold). Hill coefficients for all individual proteins were higher at pH 5.8, indicating that RNA binding is more cooperative under acidic conditions (*SI Appendix*, Table S2). The enhanced cooperativity of RNA-binding at pH 5.8 may be a result of the higher oligomerization state of P-body proteins in these conditions. These data indicate that the RNA-binding of P-body proteins is enhanced in acidic pH (Fig. 3*D*).

We next examined the location of pH-sensitive RNA-binding elements within Dcp2 since it exhibited the largest enhancement in RNA-binding affinity. A basic patch on the surface of the regulatory and catalytic domains (Dcp2^1-300^) is thought to be important for RNA binding (Fig. 2*F* and *SI Appendix*, Fig. S9*A*) (73). Within this patch, we mutated histidine 237 to alanine (Dcp2^H237A^), resulting in a modest twofold decrease in the affinity of Dcp2 for RNA at pH 5.8 (*SI Appendix*, Table S3 and Fig. 3*E*). This suggests that additional pH-sensitive RNA-binding elements are present within Dcp2. Indeed, we found that both the structured domains (Dcp2^1-300^) and the IDR (Dcp2^301-970^) each bind to RNA with similar affinity as full-length Dcp2 (*SI Appendix*, Table S3 and Figs. 2*F* and 3*E*). Furthermore, the RNA binding of both regions is also pH sensitive (*SI Appendix*, Table S3). The similar RNA-binding affinities of full-length Dcp2 and each of these regions indicates that the RNA-binding of Dcp2 may be autoinhibited, as reported for Dcp2 RNA-decapping activity (74). Thus, similar to the condensate-forming elements of Dcp2, the pH-sensitive RNA-binding elements are also distributed in both the N-terminal structured domains and the C-terminal IDR.

### Structured Domains and IDRs Contribute to Homotypic LLPS of P-Body Proteins.

**Fig. 4.**
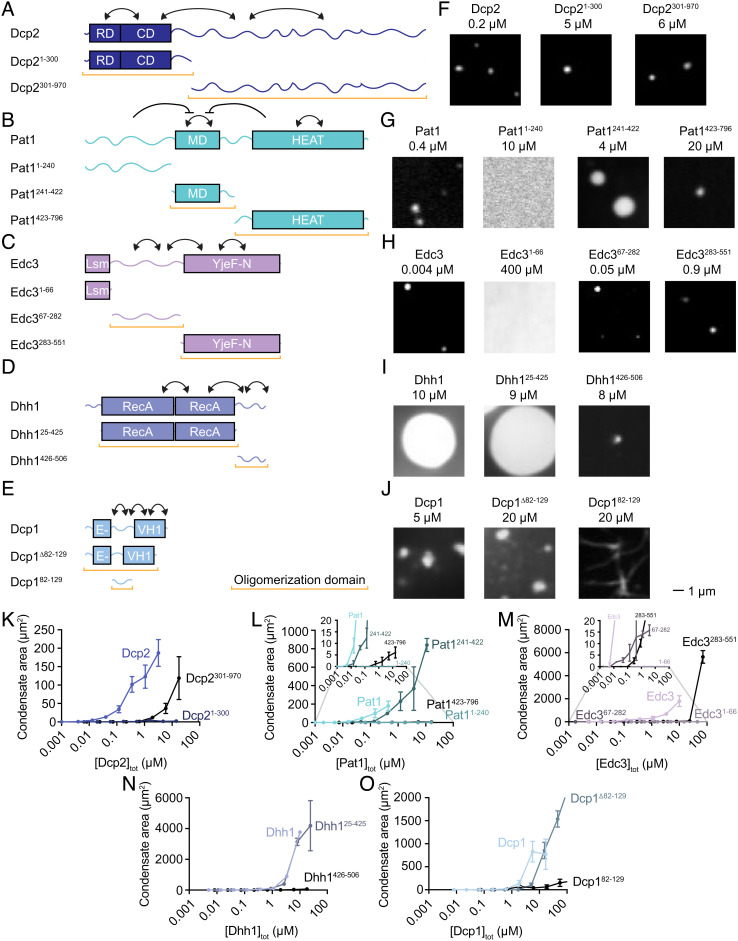
Structured domains and IDRs contribute to homotypic condensate formation. (*A*–*E*) Schematic of full-length and truncated P-body proteins. Arrows indicate inferred relationships between protein domains/IDRs as suggested from data in *K*–*O*. (*F*–*J*) Micrographs for a single concentration of each full-length and truncated P-body protein. Condensates were formed with 50 mM KOAc and pH 5.8. All truncations labeled with EGFP. (*K*–*O*) Plot of condensate area versus total protein concentration for each full-length and truncated P-body protein. *Insets* shown for clarity of datapoints with small values. See also *SI Appendix*, Figs. S12–S17.

**Fig. 5.**
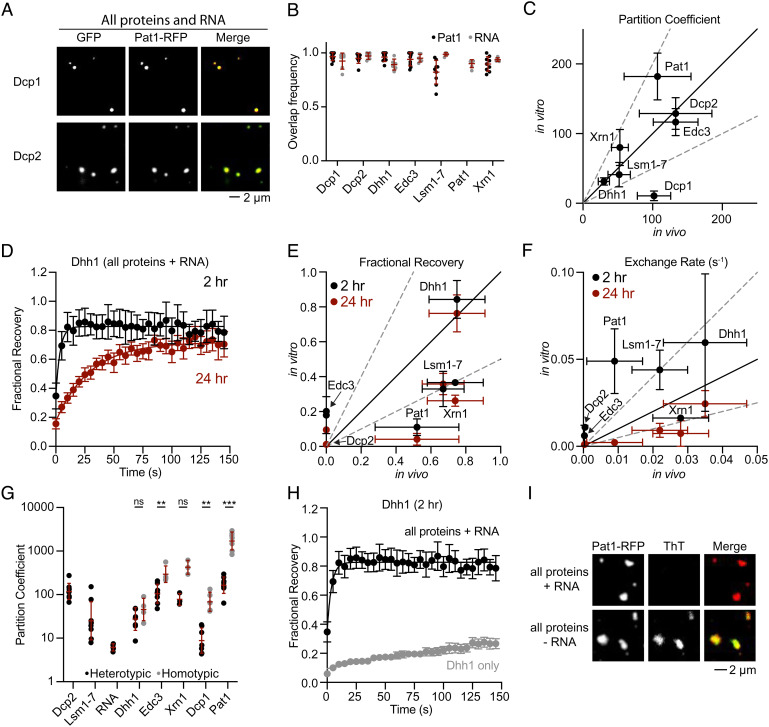
Quantitative reconstitution of P-bodies. (*A*) Micrographs examining overlap between Pat1 and Dcp1 (*Top*) or Dcp2 (*Bottom*). All experiments in Fig. 5 are at pH 5.8 and 300 mM KOAc. (*B*) Fraction overlap between different P-body proteins with Pat1 (black) and RPL41A RNA (gray). (*C*) Comparison of partition coefficients for P-body proteins in vitro (this study) and in vivo (37). Solid black line indicates equivalent values and dashed gray lines indicate twofold differences. (*D*) FRAP recovery curves for Dhh1-EGFP in condensates with all P-body proteins and RPL41A RNA. Condensates were formed for 2 h (black) and 24 h (red). (*E*) Comparison of fractional recovery values for each P-body protein with in vivo values (37). (*F*) Comparison of exchange rate values for each P-body protein with in vivo values (37). (*G*) Comparison of partition coefficients for P-body molecules in heterotypic (black) and homotypic (gray) condensates. (*H*) FRAP recovery curves for Dhh1 in heterotypic condensates (black) and homotypic condensates (gray). (*I*) Overlap between Pat1-RFP and Thioflavin T (ThT) fluorescence in condensates with (*Top* *Row*) and without (*Bottom* *Row*) RPL41A RNA. See also *SI Appendix*, Table S4 and Figs. S5 and S18–S31.

We next queried which regions of individual P-body proteins are responsible for homotypic condensate formation. We created truncations that separated structured domains from IDRs, using truncation boundaries from previous studies where available (Fig. 4 *A*–*E*) (40, 52, 54). We used condensate-promoting buffer conditions of acidic pH (5.8) and low salt (50 mM KOAc) because we hypothesized that truncations may have reduced condensate-forming capacity relative to full-length proteins. Interestingly, every protein tested contained multiple regions that are sufficient to form condensates (Fig. 4 *F*–*J* and *SI Appendix*, Figs. S12–S16). Only the N-terminal IDR of Pat1 and the structured Lsm domain of Edc3 did not form condensates at any tested concentration. These data suggest that the oligomerization of many additional P-body protein domains/regions may contribute to P-body formation (compare Figs. 1*A* and 4 *A*–*E*). Most P-body truncations formed spherical structures, with the exception of the disordered linker of Dcp1 (Dcp^182-129^) whose formations resembled interconnected fibrils (Fig. 4*J* and *SI Appendix*, Fig. S16). By comparing each full-length protein with its constituent regions, we can infer how the condensate-forming behavior of different regions contributes to that of each full-length protein (Fig. 4 *A*–*E* and *SI Appendix*, Figs. S12–S16). Pat1 has a similar C_sat_ as the middle domain, Pat1^241-422^, yet Pat1^241-422^ has a higher condensed phase concentration (Fig. 4*L* and *SI Appendix*, Fig. S13*B*). Pat1^423-796^ also self-assembles, but only at the highest concentrations tested (Fig. 4 *L*, *Inset* and *SI Appendix*, Fig. S13). Together, these data suggest that Pat1^241-422^ contributes most strongly to phase separation whereas Pat1^1-240^ and/or Pat1^423-796^ modulate the degree of concentration within the condensate. For Dcp2 and Edc3, the full-length proteins exhibit lower C_sat_ and lower condensed phase concentrations compared to each of their individual regions (Fig. 4 *K* and *M* and *SI Appendix*, Figs. S12 and S14). Edc3^1-66^ did not phase separate at any concentration tested (Fig. 4 *H* and *M*, *Inset* and *SI Appendix*, Fig. S14). Individually, Edc3^67-282^ and Edc3^283-551^ both phase separate, suggesting that these two regions contribute to phase separation of the full-length protein (Fig. 4 *H* and *M*, *Inset* and *SI Appendix*, Fig. S14). Dhh1 and Dcp1 have similar condensed phase concentrations as their structured domains alone, but adding the IDR drives the C_sat_ lower for Dcp1 (Fig. 4 *N* and *O* and *SI Appendix*, Figs. S15 and S16). These data suggest that in Dcp1, Dcp2, and Edc3, multiple regions synergize to lower their C_sat_ (Fig. 4 *A* and *C*–*E*). The region that contributes most strongly to condensate formation is a structured domain for Pat1, Dhh1, and Dcp1, whereas structured domains and IDRs are both major contributors to Dcp2 and Edc3 condensate formation. There is reasonable agreement between P-body protein regions that exhibit LLPS in vitro (Fig. 4) and contribute to P-body formation in vivo (see *SI Appendix*, Fig. S17 for further details). However, while investigating the LLPS of isolated protein regions informs on the importance of homotypic interactions, this approach does not always correlate with condensate formation in cells. For example, regions whose contributions to P-body formation are mediated via heterotypic interactions with other components will be missed by this approach (Fig. 5, *SI Appendix*, Fig. S17, and *Discussion*). Together, the data suggest that multiple regions act together to drive homotypic condensate formation of full-length P-body proteins and that both structured domains and IDRs are important contributors to this process.

### Proteins Have Similar Partitioning and Dynamics in Reconstituted and Cellular P Bodies.

Having characterized homotypic condensates of individual P-body proteins, we next examined heterotypic condensates combining all P-body proteins and RNA. All proteins and RNA were mixed together and incubated for 2 h at 30 °C before cleaving protein tags with TEV to initiate condensate formation. Initiating condensate formation earlier resulted in heterogeneous and multiphase condensates exhibiting complex architectures not observed in cellular P bodies (*SI Appendix*, Fig. S18). Condensates were incubated for 24 h before imaging unless stated otherwise. We used Pat1-mCherry and RNA-AlexaFluor647 as markers and examined colocalization with GFP-tagged or AlexaFluor488-conjugated versions of other P-body proteins (*Materials and Methods*).

Initially, we examined the full collection of molecules under acidic conditions (pH 5.8), as the previous inventory of P-body components in wild-type yeast was determined during glucose starvation, where intracellular pH is approximately 5.8 (37, 61). In acidic conditions, Pat1 frequently overlaps with all other P-body proteins (>90%), and all P-body proteins frequently overlap with RPL41A RNA (>90%: Fig. 5 *A* and *B* and *SI Appendix*, Fig. S19). These results indicate that at pH 5.8 condensates contain all eight components. EGFP, EML4-ALK [a fusion oncoprotein that forms cellular condensates (75)], and a model protein containing three repeats of the sumo-interacting motif (SIM-3R) do not partition into heterotypic condensates (*SI Appendix*, Fig. S20). The P-body protein Not1, which interacts with Dhh1 (14), is recruited into heterotypic condensates (*SI Appendix*, Fig. S20). These results suggest that the formation of, and recruitment into, condensates is dependent on specific molecular interactions. Furthermore, all condensates appeared to have homogeneous fluorescence (at the resolution limit of our confocal microscope) regardless of which components were labeled. Thus, under conditions favoring protein–protein and protein–RNA interactions, all eight P-body components together form condensates.

LLPS is sensitive to parameters such as pH, temperature, and protein concentrations (76). To examine which experimental conditions generate in vitro P bodies that most closely mimic their in vivo counterparts, we measured partition coefficients for each P-body protein while varying pH, temperature, and total protein concentrations. We compared protein partition coefficients with previously measured values in *S. cerevisiae* (37). Using the estimated cellular protein concentrations (*SI Appendix*, Fig. S5), at pH 5.8, and incubating at 30 °C resulted in the closest match to cellular P bodies (*SI Appendix*, Table S4 and Fig. S21 and Fig. 5*C*). Indeed, under these conditions, the partition coefficients for all proteins were within twofold of their in vivo values, with the exception of Dcp1. Changing to neutral pH 7, incubating the reactions at 4 °C, or using equal protein concentrations (150 nM) for every protein each resulted in greater deviation from cellular values (*SI Appendix*, Fig. S21). Each perturbation affected a unique subset of proteins. Incubating the reactions at 4 °C affected the partitioning of all proteins except for Dhh1. Incubation at pH 7 resulted in a distinct composition of condensates with RPL41A RNA, Dcp1, and Xrn1 less frequently overlapping with the other P-body proteins (*SI Appendix*, Fig. S22). We conclude that the in vitro experimental conditions of pH 5.8, 30 °C, and cellular protein concentrations most accurately recapitulate the cellular stoichiometry of P bodies.

We next investigated the role of RNA in our reconstitution. The in vitro P bodies described above were formed in the presence of RPL41A RNA, a 60-nucleotide RNA with minimal secondary structure (*SI Appendix*, Fig. S11). Removing RPL41A RNA from the reconstitution had little impact on condensate formation and protein partitioning (*SI Appendix*, Fig. S23 *A* and *B*). We tested additional RNAs including a 10-nucleotide single-stranded RNA [RNA10, (77)], full-length Mating Factor A (MFA2) mRNA which is 348 nucleotides long and localizes to cellular P bodies (56), and yeast total RNA. All RNAs were more enriched in condensates at pH 5.8 than at pH 7.0 (*SI Appendix*, Fig. S23 *C* and *D*), consistent with higher affinities between several proteins and RNA under acidic conditions (Fig. 3). Enrichment of defined RNA species in reconstituted P bodies correlates with their length (*SI Appendix*, Fig. S23E). MFA2 mRNA increased the size of in vitro condensates, although proteins partition into condensates similarly with and without this molecule (*SI Appendix*, Fig. S23 *F*–*H*). Natively folded MFA2 mRNA is required for the increase in condensate volume; heat-denatured MFA2 mRNA does not produce this effect (*Materials and Methods* and *SI Appendix*, Fig. S23 *F* and *G*). Adding a 5′ cap analog to MFA2 mRNA does not enhance P-body formation nor change protein and mRNA partitioning (*Materials and Methods* and *SI Appendix*, Fig. S23 *I* and *J*), consistent with Dcp2 binding to capped and uncapped RNA with similar affinities (78, 79). Thus, MFA2 mRNA increases the size of in vitro P bodies but is not required for their formation and does not impact protein partitioning.

Genetic studies have shown that Pat1 is an important contributor to P-body formation in cells (50, 53). To examine whether Pat1 is similarly important to our reconstitution, we held all other P-body components at their cellular concentrations but excluded Pat1. The absence of Pat1 strongly impaired condensate formation and reduced the partitioning of all other molecules into the few remaining condensates (*SI Appendix*, Fig. S24). These results confirm the importance of Pat1 for P-body formation and suggest that our reconstitution is dependent on similar molecular interactions as cellular P bodies.

To compare the dynamics of the reconstituted P bodies with their cellular counterparts, we monitored fluorescent recovery after photobleaching (FRAP) for each P-body protein in the context of the full complement of components. ATP is required for Dhh1 to have similar dynamics in vitro as in cells, likely due to the role of the nucleotide hydrolytic cycle in the binding and release of RNA by the protein (*SI Appendix*, Fig. S25) (37). We monitored the recovery of each protein at an early timepoint (2 h) and at a late timepoint (24 h) (Fig. 5*D* and *SI Appendix*, Fig. S26). At the later timepoint, most proteins demonstrated reduced fractional recovery and all proteins exhibited slower exchange rates (Fig. 5 *E* and *F*). Importantly, with the lone exception of Pat1, P-body proteins exhibited fractional recoveries and exchange rates comparable (within a factor of 2 to 4) to those observed in cells (37). The fractional recovery of Pat1, however, was lower in our in vitro reconstitution than in cells. Collectively, these data indicate that our in vitro reconstitution with seven proteins and RNA is sufficient to recapitulate the partitioning and dynamics observed in cellular P bodies for most proteins.

### Homotypic and Heterotypic Condensates Have Different Physical Properties.

Having established experimental conditions (pH 5.8, 300 mM KOAc, 30 °C, cellular concentrations of proteins) that produce native-like P bodies, we next queried whether homotypic condensates of individual proteins could form under these conditions (homotypic condensates in Fig. 2 were formed with higher protein concentrations, lower salt concentrations, and at room temperature). Individually, Dcp2, Lsm1-7, and RNA did not form homotypic condensates, indicating that their recruitment into these compartments is heterotypic in nature (Fig. 5*G* and *SI Appendix*, Fig. S27). In contrast, Dhh1, Edc3, Xrn1, Dcp1, and Pat1 each formed homotypic condensates, raising the question of whether there are any differences between homotypic condensates made up of a single P-body protein and heterotypic condensates that include all of the P-body proteins and RNA. Each protein that phase-separated individually exhibited a higher partition coefficient in homotypic condensates than in heterotypic condensates (2- to 10-fold), though this difference was only statistically significant for Edc3, Dcp1, and Pat1 (Fig. 5*G*). These results suggest that heterotypic interactions between all molecules promote a single multicomponent condensate, instead of distinct homotypic condensates. Heterotypic interactions recruit individual components that do not create condensates on their own (Dcp2, Lsm1-7, and RNA), and some heterotypic interactions temper homotypic interactions that drive Dhh1, Edc3, Xrn1, Dcp1, and Pat1 into condensates, likely through competitive binding. These results are conceptually consistent with recent descriptions of multicomponent LLPS through the framework of polyphasic linkage (*Discussion*) (36, 80, 81).

We next used FRAP experiments to determine whether homotypic and heterotypic condensates have different material properties. We examined the individual P-body proteins that form homotypic condensates under cellular conditions (Fig. 5*G*). Dhh1 is clearly less dynamic in homotypic condensates compared to heterotypic condensates at 2 h (Fig. 5*H*). Dcp1, Edc3, and Pat1 were slightly less dynamic in homotypic condensates, though the relative differences for these proteins are small since they also show low recovery in heterotypic condensates (*SI Appendix*, Fig. S28). We conclude that proteins have reduced dynamics in homotypic condensates compared to heterotypic condensates.

### RNA Delays the Maturation and Promotes the Reversibility of In Vitro P Bodies.

We used additional approaches to further dissect differences in material states between heterotypic and homotypic condensates. First, we monitored incorporation of Thioflavin T (ThT) into condensates over time. ThT is a stain for amyloid fibers, and it has been observed in other systems that in vitro condensates increasingly stain with ThT as they mature (12, 15, 82). However, we only rarely observe ThT staining of the complete condensates containing all proteins and RNA, even at longer incubations (24 h: Fig. 5*I* and *SI Appendix*, Fig. S29). Conversely, the vast majority of condensates with all P-body proteins, but without RNA, incorporated ThT at 24 h. Thus, RNA prevents maturation of reconstituted P-bodies to a state that binds ThT (likely preventing the formation of amyloid fibers). Pat1, Edc3, and Dhh1 homotypic condensates behaved similarly at 24 h; the individual protein condensates frequently stained positive for ThT in the absence of RNA, but much less frequently in the presence of RNA (*SI Appendix*, Fig. S29). Dcp1 was the lone outlier, whose homotypic condensates stained with ThT with or without RNA, consistent with the protein lacking measurable binding to RNA (*SI Appendix*, Fig. S10). These results suggest that P-body protein condensates begin to form amyloid fibers over time and that the presence of RNA inhibits or slows this transition. Consistent with these findings, the presence of RNA promotes condensate dissolution via proteolytic degradation (*SI Appendix*, Fig. S30) and arginine salt (*SI Appendix*, Fig. S31). These data also support the idea that RNA enhances the reversibility of condensates.

## Discussion

### Reconstitution of P Bodies.

**Fig. 6.**
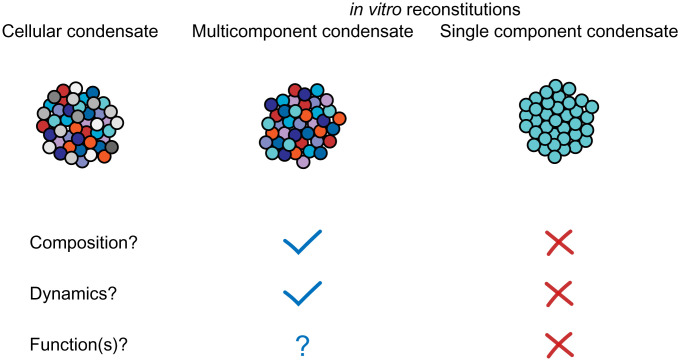
Multicomponent condensates recapitulate the composition and dynamics of highly concentrated components. Cellular condensates consist of highly concentrated molecules (colored circles) and less concentrated molecules (gray circles). Here, we found that reconstituting P bodies in vitro with all highly concentrated proteins resulted in condensates with individual components exhibiting partitioning and dynamics values in quantitative agreement with cellular P bodies (37). Reconstitutions using single components, or arbitrarily reduced complexity, often form condensates in vitro, but these condensates are distinct from cellular condensates in composition and/or material properties (59). This level of reconstitution—including three enzymes and many enzyme regulators—will allow for examination of multiple relevant biochemical activities and the interplay between these activities (*Discussion*).

In this study, we reconstituted RNA-processing bodies using seven proteins that are highly concentrated in cellular P bodies (37) and RNA. When mixed at their cellular concentrations, and under experimental conditions that mimic the cellular environment, these molecules assemble together to form condensates that contain all seven proteins and RNA. Moreover, the condensates are quantitatively consistent with cellular P bodies in terms of protein partitioning and dynamics and show a similar dependence on Pat1 (Fig. 5) (37). These results suggest that the interactions between the molecules are sufficient to encode these cellular properties (Fig. 6). While many cellular inputs could modulate the behaviors of P bodies, including additional less-enriched components, posttranslational modifications, and chemical or mechanical energy, they are not necessary to produce a compartment that is native-like in composition and dynamics. Below we discuss how our reconstitution compares to previous cellular and theoretical experiments and propose future directions for using the system to further probe multicomponent condensate formation and function.

### Integration of Homotypic and Heterotypic Interactions for P-Body Assembly.

Using conditions that mimic the cellular environment, we form condensates that contain all seven P-body proteins and RNA. However, under the same conditions, five proteins (Dhh1, Edc3, Xrn1, Edc3, and Pat1) are capable of forming homotypic condensates on their own, and these proteins have higher partitioning in homotypic compared to heterotypic condensates. These data suggest that there is a balance between homotypic and heterotypic interactions that contribute to P-body formation. Recent theoretical descriptions have combined a polyphasic linkage formalism (83) with coarse-grained linear polymer modeling to investigate which molecular features regulate the formation of multicomponent condensates (36, 80, 81). In this framework, interactions between stickers in multivalent scaffold molecules drive condensate formation whereas spacers modulate condensate formation. Ligands/clients (low-valent, nonscaffold molecules that are enriched in condensates but do not promote their formation) can modulate condensate formation by binding to stickers or spacers, with distinct expectations for the change in molecule partitioning based on the nature of the ligand and its binding site on the scaffold. In our system, Dhh1, Edc3, Xrn1, Dcp1, and Pat1 all self-associate, as each form homotypic condensates under cellular conditions (Fig. 5). Multiple stickers, consisting both of structured domains and IDRs, are present within these proteins (Fig. 4 and *SI Appendix*, Fig. S1). Interactions between different molecules (37, 51) drive the complete collection to form a single condensate including all molecule types. Based on the lower partitioning of many of the proteins in heterotypic condensates, there are likely some heterotypic interactions that disrupt sticker–sticker interactions. For example, Pat1 is an important scaffold-like protein for P-body formation (50, 53), and Pat1 interacts with itself and with all of the other highly concentrated P-body proteins and RNA (54, 84–86). In contrast, since Lsm1-7 only interacts with RNA and Pat1 (54, 87), Lsm1-7 binding to Pat1 may compete with other Pat1-mediated interactions and thus cap the overall valency of the system. Cellular data potentially supports this limiting function of Lsm1-7 as genetic deletion of Lsm1 enhances P-body formation in yeast cells (50). However, this strain also exhibits an RNA-decapping defect and accumulates deadenylated mRNAs which may also contribute to P-body formation (88, 89). Other heterotypic interactions will likely have minimal effect on scaffold partitioning, either by binding to spacers within the scaffold molecules or by forming a new set of interactions that is comparable in valency to those disrupted in the scaffold molecule. The Dhh1–Pat1 interaction (54, 85) appears to be in this latter class as deletion of Dhh1 has minimal impact on P-body formation and the partitioning of other P-body proteins in yeast cells (14, 50). A general and important point here is that the complexity of interactions among highly concentrated P-body components is not likely to be recapitulated in simplified systems examining only a few components at a time. Thus, the formation of homotypic condensates will not always be relevant in the context of cellular multicomponent condensates where a combination of homotypic and heterotypic interactions impart thermodynamic and material properties to the system. Additional experiments are needed to further interrogate which heterotypic interactions strongly modulate P-body formation and composition.

Our results suggest that cytoplasmic acidification contributes to P-body formation. Prior in vivo studies indicated that cytoplasmic acidification restricted the mobility of mRNAs (60, 61). This reduced mobility may be due to the formation of P bodies, as we found that acidic pH promotes homotypic protein–protein interactions and heterotypic protein–RNA interactions through protonation of a key histidine residue on Dcp1, and likely multiple histidine residues dispersed throughout the other P-body proteins (Figs. 2 and 3). Importantly, acidic pH is also required to generate molecular stoichiometry in our reconstitution consistent with cellular P bodies measured in glucose starvation conditions, where the cytoplasm is acidic (Fig. 5) (37, 61). It is possible that the absence of macroscopic P bodies during cellular proliferation may be due in part to the more basic cytoplasmic pH under these conditions, but additional cellular mechanisms likely contribute to repressing P-body formation. However, no additional regulatory mechanisms are apparently required for the formation of P bodies with appropriate molecular stoichiometry and material properties (Figs. 5 and 6).

### Reconstitution Mimics the Redundant Nature of Cellular Assemblies.

Our in vitro results are consistent with previous genetic experiments indicating that no single protein is absolutely required for P-body formation in vivo, although some are more important than others (50–53). Our results are also consistent with previous reconstitutions that used a more limited set of P-body proteins, in that several distinct minimal collections of P-body proteins are able to form condensates in vitro (14, 21, 47–49). These results suggest that there are multiple, somewhat redundant, protein scaffolds that can act collectively to assemble many other P-body components (51). Excluding Pat1 restricts, but does not completely eliminate, the formation of condensates. Why does the loss of Pat1 dampen condensate formation when remaining individual proteins (Dhh1, Edc3, Xrn1, and Dcp1) robustly form homotypic condensates on their own? Our current interpretation of these results is that heterotypic interactions restrict the homotypic interactions of the remaining scaffold-like proteins when Pat1 is removed. This interpretation suggests that there is epistasis in P-body formation; that is, the contribution of any given molecule to condensate formation is dependent on which other molecules are present (9, 12, 35, 36, 55). If epistasis is a general feature of condensate formation, then reconstitutions that approach the molecular complexity of cellular condensates will more accurately reveal the contributions that each molecule makes to condensate formation. This reconstitution best mimics cellular P bodies when using physiological protein concentrations (Fig. 5). Using different absolute or relative concentrations of components, and/or a different collection of molecules, will likely influence the epistatic interactions between components. Thus, an important future direction will be to understand quantitatively how the constituent molecules influence the formation of P bodies (i.e., the threshold concentrations for appearance of the compartments) and their composition.

The role of RNA in the formation and maintenance of P bodies is not fully understood. In biochemical reconstitutions, one report described a strong effect of RNA on the threshold concentrations of Dcp1/Dcp2/Edc3 for phase separation (47). Another, however, observed that RNA does not change the partitioning of Dcp1/Dcp2/Edc3 enrichment in phase-separated droplets, except when Edc3 is in large (16-fold) excess over the other proteins, suggesting that RNA does not strongly promote phase separation (21). Here, we have observed that RNA does not change the partitioning of proteins into P bodies and is not required for their formation at cellular concentrations (*SI Appendix*, Fig. S23). MFA2 RNA, the longest (348 nucleotides) and most structured RNA we examined (*SI Appendix*, Fig. S11), modestly increased condensate size (*SI Appendix*, Fig. S23). It is possible that long RNAs (1,000 + nucleotides) that are highly enriched in P-bodies (30–32) would have more substantial impact on P-body formation and protein partitioning. Similar ambiguity has been reported in more physiological systems. In cell lysates, digestion of RNA dissolves yeast P bodies (56), and in cells, P bodies form in proportion to the pool of deadenylated mRNAs (43, 55). Yet, it was recently demonstrated that acute RNA degradation by RNAse L does not disrupt P bodies in intact human cells (57). These apparent contradictions could be explained by either some residual RNA fragments contributing to P-body persistence in mammalian cells after RNase L activation, or that RNA may be important for the initiation of P bodies in vivo, but not for their maintenance. Thus, long timescale genetic perturbations may prevent P-body formation, while acute depletion of mRNA does not affect existing P bodies. Additional studies of the time dependence of RNA perturbations in vivo and in vitro may help resolve this issue.

Here, we found that RNA is important for promoting the reversibility of P bodies and delaying their maturation into an irreversible, nondynamic, amyloid-like state (Fig. 5). The RNA recognition motifs of Poly(A)-binding protein (Pab1) undergo partial unfolding in condensates (90). Similar partial unfolding might be occurring here for P-body proteins in the absence of RNA. By stabilizing folded domains, RNA could prevent this effect and delay maturation. In general, the ability of RNA to prevent protein maturation in condensates (91) may be related to the ability of RNA to chaperone aggregation-prone proteins (92). Many P-body protein regions that interact with RNA also mediate homotypic interactions (12, 47, 52–54, 70). For example, the central IDR of Edc3 binds to RNA, interacts with the C-terminal YjeF-N domain, and promotes LLPS (47, 70). Thus, one mechanism by which RNA promotes a reversible state of P bodies may be by restricting homotypic protein interactions. Consistent with our findings, P-bodies are relatively liquid-like in yeast and only progress to a solid state when individual proteins are artificially overexpressed (82). This suggests that the stoichiometry of resident protein and RNA molecules regulates the material properties of P bodies. In this way, stoichiometry could also regulate P-body function, as observed for reconstituted membrane-associated signaling condensates, where stoichiometry controlled membrane dwell time and consequently activity (20, 22).

### Potential Implications of Multiple Scaffold-Like Molecules.

One feature enabled by a multiscaffold system is the colocalization and coordination of multiple enzymatic activities, as well as regulators of those activities. It remains unclear whether P bodies function as microreactors to enhance RNA degradation rates (21, 43), or as storage compartments to protect RNA and proteins from degradation during periods of cellular stress (44, 46, 47). It is also possible that yeast and mammalian P bodies may be functionally different. P bodies may activate RNA degradation as they concentrate RNA-helicase (Dhh1), RNA-decapping (Dcp2), and RNA-exonucleolytic degradation (Xrn1) activities within distinct polypeptides (38–41). Furthermore, Dcp1, Dhh1, Edc3, and Pat1 are known to stimulate the activity of Dcp2 (41, 54). Thus, forming a heterotypic condensate that includes all of these proteins could in theory serve as a concentrated microreactor to increase RNA degradation. Recent work demonstrated that Dcp2-mediated RNA decapping is enhanced within in vitro condensates containing Dcp1, Dcp2, and Edc3, but is inhibited in condensates that only had Dcp1 and Dcp2 (21). This example illustrates how composition can modulate enzymatic activities within condensates and highlights the importance of efforts to mimic cellular complexity when investigating the biochemical activities of condensates. In addition to individual biochemical activities being increased within P bodies, the transfer of substrate RNA between different types of enzymes could also be enhanced, particularly if certain molecules are spatially organized within the condensates (93, 94), potentially resulting in a significant increase in the overall rate of RNA degradation. This reconstitution, which includes all highly enriched P-body proteins and many activators, is suitable for future examination of enzymatic activities and pathway flux within P bodies.

Combined with genetic experiments, our in vivo (37) and in vitro examinations of P bodies provide a route to systematically determine which components play important roles in forming condensates and specifying their physical properties. The ability to form homotypic condensates in vitro is often considered evidence that a molecule plays an important scaffolding role in vivo. However, given the prevalence of proteins that can undergo phase separation in vitro (95), the redundant scaffolding observed in P bodies may be a common feature of many cellular condensates. This view is consistent with recent network-based models for condensate formation, where multiple macromolecules can contribute to different degrees, based on their positions in the molecular interaction network and degree of connectivity to other components (8, 10, 37). In future biochemical studies, it will be important to examine candidate scaffolds in experimental contexts that closely mimic cellular conditions in order to understand how multiple molecule types can be integrated to form multicomponent condensates. Using quantitative, multicomponent reconstitutions will enable a better understanding of the formation mechanisms, and ultimately biochemical and cellular functions, of diverse biomolecular condensates.

### Materials and Methods.

Proteins were expressed in *Escherichia coli* and *S. cerevisiae* and purified using affinity, ion exchange, and size exclusion chromatography. Micrographs were obtained with a Nikon Eclipse Ti microscope base with a Yokogawa CSU-X1 spinning disk confocal scanner unit, 100 X 1.49 NA objective, and Andor EM-CCD camera. The following *t* tests were performed: two-tailed paired in Figs. 2 *E* and *G* and 5*G* and *SI Appendix*, Figs. S23 and S31; one-tailed paired in *SI Appendix*, Figs. S18*H* and S29; two-tailed heteroscedastic in Fig. 3*E*. See Extended Methods for further details.

## Supplementary Material

Appendix 01 (PDF)

## Data Availability

Microscopy images data have been deposited in Zenodo (96).
