# Supplementary material for: Quantitative reconstitution of yeast RNA processing bodies
Source: Proc Natl Acad Sci U S A. 2023 Mar 27;120(14):e2214064120. doi: 10.1073/pnas.2214064120 (PMC10083542; doi:10.1073/pnas.2214064120)
Supplement: Supplementary file 1 — Appendix 01 (PDF) [file pnas.2214064120.sapp.pdf]

Supplemental Information for

Quantitative reconstitution of yeast RNA processing bodies

Simon L Currie[1], Wenmin Xing[1], Denise Muhlrad[2], Carolyn J Decker[2], Roy Parker[2], Michael K Rosen[1]

[1]Department of Biophysics, Howard Hughes Medical Institute, UT Southwestern Medical Center, Dallas TX, 75390
[2]Department of Biochemistry, Howard Hughes Medical Institute, University of Colorado, Boulder CO, 80309

Corresponding author: michael.rosen@utsouthwestern.edu (M.K.R.)

This PDF file includes:
Extended Methods
Tables S1 to S5
Figures S1 to S36
Supplemental References

EXTENDED METHODS

Cloning of expression plasmids

We examined seven proteins that are highly concentrated in *S. cerevisiae* P bodies: Dcp1, Dcp2, Dhh1, Edc3, Lsm1-7, Pat1, and Xrn1 (Fig. 1A) (1). We did not include Upf1 because it does not affect the targeting of most normal mRNAs to P bodies, but is more of an accessory factor affecting only a subset of mRNAs undergoing aberrant translational termination and nonsense-mediated decay (NMD) (2). Genes for Dcp1, Dcp2, Dhh1, Edc3, and Pat1 were cloned into a modified pMAL plasmid (New England Biolabs) using standard methods and NdeI and BamHI restriction sites. In order to use this strategy, silent mutations were first inserted into the coding sequences to eliminate restriction sites in Dcp2 (one NdeI site), Edc3 (one BamHI site), and Pat1 (two NdeI sites), using standard site directed mutagenesis. The modified pMAL plasmid (pMTTH) contains TEV-cleavable N-terminal MBP and C-terminal His_6 tags. These genes were also cloned into a modified MTTH vector with monomeric EGFP cloned in after the P-body gene and before the second TEV cleavage site/His_6 tag.

Expression plasmids for Lsm1-7 and Xrn1 were generous gifts from Yigong Shi and Lionel Benard, respectively.

Protein expression and purification

Lsm1-7 and Xrn1 were expressed and purified as described previously (3-5). The general protocol for expressing and purifying the other P-body proteins is described below, with protein-specific information provided thereafter. Plasmids were transfected into *Escherichia Coli* BL21 (DE3) cells and grown overnight at 37 °C on Luria Broth (LB)/Ampicillin agar plates. Individual colonies were

resuspended in a 50 mL culture of LB/Ampicillin and grown overnight at 37 °C. Cells were collected by centrifugation (3,400 x *g*, 10 minutes), resuspended in LB, and added to six, one-liter cultures of LB/Ampicillin in four-liter unbaffled flasks. Cultures were grown at 37 °C until an OD_{600} of ~ 0.5 at which point the temperature was decreased to 18 °C. Cultures were induced with 1 mM IPTG at an OD_{600} of ~ 1.0 and grown overnight at 18 °C. Cells were collected by centrifugation (4,700 x *g*, 40 minutes) and the pellet was resuspended in 25 mM Tris pH 8, 10% (V:V) glycerol, 500 mM NaCl, 10 mM imidazole, and 5 mM β-mercaptoethanol (BME). Suspensions were transferred to 50 mL conical tubes and stored at -80 °C for future use.

Cell suspensions were thawed in cold water and lysed using a cell homogenizer (10,000 psi, 3 passes on ice). Lysate was centrifuged (45,000 x *g*, 30 minutes) and the cleared supernatant was added to ~ 15 mL of Ni^{2+} agarose resin (Biorad), and incubated for 30 minutes with circular rotation in 50 mL conical tubes. Proteins were isolated using gravity columns and the following buffers. Ni^{2+} wash 1: 25 mM Tris pH 8, 10% glycerol, 2.5 M NaCl, 10 mM imidazole, 5 mM BME; Ni^{2+} wash 2: 25 mM Tris pH 8, 10% glycerol, 500 mM NaCl, 10 mM imidazole, 5 mM BME; Ni^{2+} elution: 25 mM Tris pH 8, 10% glycerol, 500 mM NaCl, 500 mM imidazole, 5 mM BME. The eluate was added to ~ 30 mL of amylose resin (New England Biolabs) and incubated for 30 minutes with circular rotation in 50 mL conical tubes. Proteins were isolated using gravity columns and the following buffers. Amylose wash 1: 25 mM Tris pH 8, 10% glycerol, 150 mM NaCl, 5 mM BME; amylose elution: 25 mM Tris pH 8, 10% glycerol, 150 mM NaCl, 5 mM BME, and 50 mM maltose.

The amylose eluate was diluted threefold into 25 mM Tris pH 8, 0 mM NaCl, and 5 mM BME for a final concentration of 50 mM NaCl, and filtered through a 0.45 µm Whatman filter (GE Healthcare). Filtrate was loaded onto a Q sepharose ion-exchange column and eluted using a 50

– 500 mM NaCl gradient over ten column volumes, taking fractions every 2.5 mL. Fractions containing the desired protein were loaded onto an SD200 26/600 size exclusion column equilibrated with 10 mM MES pH 7, 5% glycerol, 300 mM KOAc, and 5 mM BME. Fractions containing the desired protein were concentrated by ultrafiltration (Amicon centricon) with 3k (Lsm1-7) and 30k (all other proteins) molecular weight cutoffs. Single-use aliquots were flash frozen in liquid nitrogen and stored at -80 °C for future use.

Cleaving the MBP-fusion tag before ion-exchange and size-exclusion columns resulted in a partial loss of protein for Dhh1 and a total loss of protein for Edc3 and Pat1. Thus, to simplify procedures and maintain consistency, the MBP-tag was retained on all proteins during purification, and condensate formation was initiated by TEV cleavage.

Protein-specific deviations from the general protocol are as follows. Only two liters of culture were used for Dcp1 and Dhh1. Dcp2 was expressed in terrific buffer (TB) instead of LB due to low expression of the full-length protein. 10 mM ATP was included in the wash buffers for the Ni^{2+} column for Dhh1 in order to disrupt interactions with RNA. Without ATP, Dhh1 eluted as a series of peaks from the ion-exchange column presumably due to an inhomogeneous distribution of bound RNA molecules. 1M urea was included in the ion-exchange buffers for Pat1 to prevent aberrant oligomerization and/or precipitation. The size exclusion buffer for Pat1 included 1 M KOAc to similarly eliminate aberrant oligomerization and/or precipitation.

EGFP-fusion proteins for Dcp1, Dcp2, Dhh1, Edc3, and Pat1, and mCherry-fusion protein for Pat1 were purified the same as described above. Lsm1-7 and Xrn1 were labeled with maleimide AlexaFluor488 (Thermo Fisher Scientific). Lsm1-7 and Xrn1 were labeled overnight with a 5:1 AlexaFluor488:protein ratio, after last ion-exchange column and before size exclusion

column. Free dye was purified away using a 5 mL Desalting HP column (Cytiva), before the labeled protein was purified using size exclusion.

See Table S5 for protein sequences.

RNA reagents

RPL41A and RNA10 RNA (Fig. S11) were purchased from Integrated DNA Technologies, resuspended in 10 mM Tris-HCl, 1 mM EDTA, pH 7.0 buffer, and single-use aliquots were flash frozen in liquid nitrogen and stored at -80 °C for future use. Total yeast RNA was purchased from Sigma-Aldrich and solubilized and stored as described above. MFA2 RNA was generated using *in vitro* transcription. Template DNA was generated by PCR out of the pRP802 plasmid (Parker lab) using the following oligos: TAATACGACTCACTATAGCGAGC and TTTTTCATGAAAAAATCTGTTAAA-GTGATAACTAC. Note that RPL41A and MFA2 RNAs are oligoadenylated as such RNAs are bound by Lsm1-7 and Pat1 with tighter affinity than polyadenylated RNAs (6). MFA2 RNA was transcribed from this DNA template using the Invitrogen Ambion MEGAscript T7 Transcription Kit by following the standard protocol with 15% Cy5-UTP included. The reaction was allowed to proceed overnight, then treated with the Megaclear Transcription Clean-Up Kit (Thermo Scientific). MFA2 RNA with 5’ cap analog [m7G(5’)ppp(5’)G: Thermo Fisher Scientific] was transcribed as above but with a 8:1 cap analog:GTP ratio. A sample of RNA was run on a 5% polyacrylamide/8 M urea gel to confirm correct size and specificity. Extent of Cy5 labeling was determined using a NanoDrop spectrophotometer. MFA2 RNA samples were flash frozen in liquid nitrogen and stored at -80 °C for future use.

Protein structure and disorder predictions

Structured and disordered regions were determined based on the following criteria (Fig. 1A and S1). Known three-dimensional structures were curated from the literature (7-13). Secondary structure and protein disorder were predicted using PSIPRED and Disprot VSL2, respectively (14, 15). Pat1 middle domain (MD: $Pat1^{241-422}$) was the only region in these P-body proteins that had predicted secondary structure without a previously determined three-dimensional structure (Fig. S2 and S3). We predicted the structure of $Pat1^{241-422}$ using AlphaFold (Fig. S2F)(16, 17). This model confidently predicts multiple α-helices within $Pat1^{241-422}$, consistent with disorder prediction (Fig. S3). However, we note that while the AlphaFold model confidently predicts the presence of α-helices within $Pat1^{241-422}$, their orientation relative to one another and relative to the rest of Pat1 is unclear (see Fig. S2 for further details). The Dcp1 model (Fig. 2C) was made by overlapping structures for the Dcp1 dimer (18) with Dcp1-Xrn1 (9) and Dcp1-Dcp2 (12) interactions using the main chain of Dcp1 for alignment. The Dcp2 homology model (Fig. S9A) was generated by mutating *S. cerevisiae*-specific residues onto the *Schizosaccharomyces pombe* structure (19). All protein structures were visualized using PyMol.

Microscopy

Microscopy experiments were carried out in 384-well glass bottom microwell plates (Brooks Life Science Systems: MGB101-1-2-LG-L). Prior to use the plates were cleaned with 5% Hellmanex III, then 1M NaOH, and then passivated with mPEG silane, with extensive milliQ water washes between each step. On the day of use individual wells were blocked with 2% bovine serum albumin, then rinsed thoroughly with reaction buffer. Single-use aliquots of proteins were quickly

thawed in cold water, centrifuged at 16,000 x *g* for 10 min at 4 °C, then stored on ice. Protein concentrations were quantified using UV-Vis spectroscopy (absorbance at 280 nm) and rechecked before each experiment. For most microscopy experiments single-use RNA aliquots were thawed on ice and heated to 70 °C for 90 s immediately before use. For small RNAs that lack significant secondary structure (RNA10, RPL41A), heat-denatured and native RNA had the same impact on condensate formation. However, MFA2, the longest RNA we tested with extensive secondary structure, promotes P body formation by increasing the volume fraction of the dense phase even though protein partitioning is not changed. Thus, we recommend not heat denaturing long mRNAs with extensive secondary structure for future experiments. For complex reactions, the components were added to the well in the following order to promote heterotypic condensate formation and minimize homotypic condensate formation: buffer, RNA, Dcp2, Lsm1-7, Xrn1, Dhh1, Pat1, Edc3, and Dcp1. Pipetting into plate wells was carried out at room temperature as quickly as possible, then plates were incubated at 30 °C for 2 hours. TEV was added at a 1:50 molar ratio, relative to total protein and RNA concentrations, to initiate condensate formation. We note that different procedures, e.g. involving TEV cleavage before mixing and/or insufficient preincubation of P-body components, yielded heterogeneous condensates and poor reproducibility. Reactions were incubated and imaged at 30 °C unless stated otherwise.

Condensate images were captured on a Nikon Eclipse Ti microscope base with a Yokogawa CSU-X1 spinning disk confocal scanner unit, 100 X 1.49 NA objective, and Andor EM-CCD camera. Fluorescence Recovery After Photobleaching (FRAP) was performed with a TIRF/iLAS2 FRAP Module (Biovision) and Rapp UGA-40 Phototargeter. Z-stacks were collected

with the exception of FRAP experiments as the rapid collection of subsequent timepoints did not allow for the collection of a Z-stack at each time point.

Micrographs were analyzed using FIJI (20). For Z-stacks the Z plane with the highest intensity was selected for analysis. A threshold was set at three-fold above the background signal for each channel. Concentrations were determined from fluorescence intensities using standard curves for RFP (Pat1), Alexa Fluor 488 (Lsm1-7 and Xrn1), and GFP (Dcp1, Dcp2, Dhh1, Edc3, and Pat1) (Fig. S32-34). Small condensates, that are close in size (xy area) to the point spread function (PSF) of a microscope exhibit diluted intensities (1). We empirically determined that the intensity of condensates smaller than 2.5 µm in diameter exhibit a linear dependence on condensate size, whereas the intensities of condensates larger than 2.5 µm in diameter were independent of condensate size (Fig. S35). Thus condensates larger than 2.5 µm in diameter were selected for further analysis of condensate concentration. We used a fluorescence intensity cut-off of threefold above background for each fluorophore, and assigned the center of mass for condensates in each of the microscope channels. Condensates with centers of mass within 1 µm were considered to be overlapping to account for slight discrepancies in the designation of the center of mass and for stage drift during imaging.

RNA is not degraded by Xrn1, or any potentially contaminating host proteins, during the course of microscopy experiments (Fig. S36). Xrn1 is not active in these experiments for two reasons. 1) The defined RNA species used in these experiments are not 5' monophosphorylated, which is the preferred substrate for Xrn1 (13). 2) Even in experiments when the preferred 5' monophosphorylated are used (not in this article), Xrn1 is not active in the absence of Mg^{2+} (13). Our experiments in this article lack Mg^{2+}. In further experiments we have formed condensates

with P-body proteins and monophosphorylated RNA. RNA is stable within these condensates for hours and is not degraded by Xrn1 until Mg^{2+} is added to the reaction.

50 µM ThT was added to samples to image ThT staining of condensates. For imaging condensates with all P-body proteins, with or without RNA, and for Pat1 homotypic condensates, Pat1-RFP was used as a marker for the condensates. Condensates were considered to stain positive for ThT if the centers of mass from the RFP and ThT channels were within 1 µm of each other (Fig. 5I and S29). ThT staining cannot be used when GFP is the marker for a condensate due to the overlap between GFP and ThT fluorescence spectra. Instead, for Dcp1, Dhh1, and Edc3 homotypic condensates we estimated the percentage of ThT positive condensates as follows. We performed parallel experiments with either GFP-tagged proteins only to count the number of condensates, or with unlabelled proteins and ThT to count the number of ThT positive condensates. We estimated the percentage of ThT positive condensates by dividing the number of ThT positive condensates by the number of total condensates (Fig. S29).

For the proteolysis experiments 0.8 µg trypsin was added to each reaction. The condensed phase concentration for Pat1-RFP and Dhh1-GFP were monitored before and after trypsin addition. Relative condensed phase protein concentrations based on pre-trypsin levels are displayed (Fig. S30).

Electrophoretic Mobility Shift Assays

Reactions included 10 nM Alexa Fluor647-labeled RPL41A RNA. P-body proteins were titrated over >100-fold range, with exact values depending on expression/purification yield and affinity for RNA (Fig. S10), in 300 mM KOAc, 10 mM MES pH 7 or pH 5.8, and 5 mM BME. Reactions were

incubated on ice for 2 hr, then resolved on a 6% native PAGE gel. 0.5x MBE buffer (MES, pH 7 or 5.8; Borate; EDTA) and gels were equilibrated and run at 4 °C. Gels were imaged using Bio-Rad ChemiDoc MP. Bands were detected using Bio-Rad Image Lab software (v. 6.1) and curves were fit using the Specific Binding with Hill Slope equation on Graphpad Prism (v. 9.0.0 for Mac).

Native Gels

Reactions with Dcp1 were incubated at room temperature at the indicated pH (5.8 or 7) without TEV cleavage for 2 hours. 2x sample buffer (62.5 mM MES pH 7, 25% glycerol, 1% bromophenol blue) was then added and samples were loaded onto a native polyacrylamide (37.5:1 acrylamide:bis-acrylamide) gel with 4% stacking and 6% separating portions. Gels used 25 mM MES pH 7 as the buffer. Running buffer with 25 mM MES pH 7 and 192 mM Glycine was used and gels were pre run for 30 minutes before loading samples.

Quantification and Statistical Analyses

Data points in Fig. 1, 3, and 5, and accompanying supplemental figures, correspond to mean and standard deviation from at least three replicate experiments. Data points in Fig. 2, 4, and accompanying supplemental figures, correspond to individual condensate values from at least two experiments. All statistical tests were performed using Microsoft Excel. A two-tailed paired t test was performed on the data in Fig. 2E, 2G, 5G, S23, and S31. A one-tailed paired t test was performed on Fig. S18H and S29. A two-tailed heteroscedastic t test was performed on the data in Fig. 3E. *, **, and *** symbols correspond to p values less than 0.05, 0.01, and 0.001, respectively.

Table S1.
Effect of gene deletion on P-body formation

Gene deletion	Effect on other proteins in P bodies[#] Increased	Decreased
pat1Δ	-	Dcp1, Dcp2, Dhh1, Edc3, Lsm1, Xrn1
edc3Δ	-	Dcp1, Dcp2, Dhh1, Lsm1, Pat1, Xrn1
dhh1Δ	-	Dcp2
dcp2Δ[$]	Dhh1, Edc3, Lsm1, Pat1, Xrn1	Dcp1
lsm1Δ	Dcp1, Dcp2, Dhh1, Edc3, Xrn1	Pat1[%]
xrn1Δ	Dcp1, Dcp2, Dhh1, Edc3, Lsm1, Pat1	-
dcp1Δ	Dcp2, Dhh1, Edc3, Lsm1, Pat1, Xrn1	-

[#] References: (21-23)
[$] Dcp2 deletion increases P-body formation during mid-log phase but decreases P-body formation under stress conditions, suggesting it has competing scaffolding and RNA-degradation activities that are integrated to either form or dissolve P bodies.
[%] Lsm1 deletion reduces Pat1 accumulation in P bodies by enhancing nuclear retention of Pat1.

Table S2.
Curve fitting for individual P-body proteins binding to RNA (Figs. 3B and S10)

Protein	pH	K_D (μM)	K_D (pH 7) / K_D (pH 5.8)	Hill Coefficient[a]	B_{max}[b]	n
Dcp1	7	> 20	-	-	-	3
	5.8	> 20		-	-	3
Dcp2	7	> 20	> 16	-	-	3
	5.8	1.3 ± 0.1		1.7 ± 0.4	1.0 ± 0.1	3
Dhh1	7	0.7 ± 0.1	2.6 ± 0.3	2.2 ± 0.1	1.0 ± 0.1	3
	5.8	0.3 ± 0.1		2.7 ± 0.6	1.0 ± 0.1	3
Edc3	7	2.1 ± 0.8	3 ± 1	1.7 ± 0.1	1.3 ± 0.3	3
	5.8	0.7 ± 0.2		2.9 ± 0.9	1.0 ± 0.1	3
Lsm1-7	7	0.5 ± 0.1	1.7 ± 0.6	3 ± 1	1.0 ± 0.1	3
	5.8	0.3 ± 0.1		4.2 ± 0.6	1.0 ± 0.1	3
Pat1	7	0.7 ± 0.2	1.8 ± 0.6	1.5 ± 0.8	1.0 ± 0.1	3
	5.8	0.4 ± 0.1		2.5 ± 0.7	1.0 ± 0.1	3

[a] Hill coefficient was not determined (-) for conditions that did not reach saturation.
[b] B_{max} was not determined (-) for conditions that did not reach saturation.

Table S3.
Curve fitting for Dcp2 truncations and point mutants binding to RNA (Fig. 3E).

Protein	pH	K_D (μM)	Fold difference[a]	Hill coefficient[b]	B_{max}[c]
Dcp2$^{1-300}$	7	>20	-	-	-
	5.8	1.7 ± 0.3	1.3 ± 0.3	2.2 ± 0.6	1.0 ± 0.1
Dcp2$^{301-970}$	7	> 20	-	-	-
	5.8	1.9 ± 0.5	1.5 ± 0.4	1.3 ± 0.4	1.0 ± 0.1
Dcp2^{H237A}	7	>20	-	-	-
	5.8	2.8 ± 0.8	2.2 ± 0.6	3	0.9 ± 0.1

[a] Fold difference relative to Dcp2 (Table S2).

[b] Hill coefficient was not determined (-) for conditions that did not reach saturation.

[c] B_{max} was not determined (-) for conditions that did not reach saturation.

Table S4.
Partition coefficients for P-body proteins in heterotypic condensates (Fig 5C).

Protein	Partition Coefficient	Fold difference[a]	n
Dcp1	11 ± 7	0.10 ± 0.07	8
Dcp2	130 ± 20	1.0 ± 0.2	8
Dhh1	31 ± 5	1.0 ± 0.2	8
Edc3	120 ± 20	0.9 ± 0.2	8
Lsm1-7	40 ± 20	0.8 ± 0.3	8
Pat1	180 ± 30	1.7 ± 0.4	6
Xrn1	80 ± 30	1.5 ± 0.6	4

[a] Fold difference relative to *in vivo* values (1).

Table S5.
Detailed information on recombinant proteins used in this study.

Protein	Sequence
Dcp1	MTGAATAAENSATQLEFYRKALNFNVIGRYDPKIKQLLFHTPHASLYKWDFKKDEWNKLEYQ<br>GVLAIYLRDVSQNTNLLPVSPQEVDIFDSQNGSNNIQVNSGSDNSNRNSSGNGNSYKSNDSL<br>TYNCGKTLSGKDIYNYGLIILNRINPDNFSMGIVPNSVVNKRKVFNAEEDTLNPLECVGVEVK<br>DELVIIKNLKHEVYGIWIHTVSDRQNIYELIKYLLEYEPRDSCA
Dcp1^{H40A}	MTGAATAAENSATQLEFYRKALNFNVIGRYDPKIKQLLFATPHASLYKWDFKKDEWNKLEYQ<br>GVLAIYLRDVSQNTNLLPVSPQEVDIFDSQNGSNNIQVNSGSDNSNRNSSGNGNSYKSNDSL<br>TYNCGKTLSGKDIYNYGLIILNRINPDNFSMGIVPNSVVNKRKVFNAEEDTLNPLECVGVEVK<br>DELVIIKNLKHEVYGIWIHTVSDRQNIYELIKYLLEYEPRDSCA
Dcp1^{H43A}	MTGAATAAENSATQLEFYRKALNFNVIGRYDPKIKQLLFHTPAASLYKWDFKKDEWNKLEYQ<br>GVLAIYLRDVSQNTNLLPVSPQEVDIFDSQNGSNNIQVNSGSDNSNRNSSGNGNSYKSNDSL<br>TYNCGKTLSGKDIYNYGLIILNRINPDNFSMGIVPNSVVNKRKVFNAEEDTLNPLECVGVEVK<br>DELVIIKNLKHEVYGIWIHTVSDRQNIYELIKYLLEYEPRDSCA
Dcp1^{H198A}	MTGAATAAENSATQLEFYRKALNFNVIGRYDPKIKQLLFHTPHASLYKWDFKKDEWNKLEYQ<br>GVLAIYLRDVSQNTNLLPVSPQEVDIFDSQNGSNNIQVNSGSDNSNRNSSGNGNSYKSNDSL<br>TYNCGKTLSGKDIYNYGLIILNRINPDNFSMGIVPNSVVNKRKVFNAEEDTLNPLECVGVEVK<br>DELVIIKNLKAEVYGIWIHTVSDRQNIYELIKYLLEYEPRDSCA
Dcp1^{H206A}	MTGAATAAENSATQLEFYRKALNFNVIGRYDPKIKQLLFHTPHASLYKWDFKKDEWNKLEYQ<br>GVLAIYLRDVSQNTNLLPVSPQEVDIFDSQNGSNNIQVNSGSDNSNRNSSGNGNSYKSNDSL<br>TYNCGKTLSGKDIYNYGLIILNRINPDNFSMGIVPNSVVNKRKVFNAEEDTLNPLECVGVEVK<br>DELVIIKNLKHEVYGIWIATVSDRQNIYELIKYLLEYEPRDSCA
Dcp1$^{\Delta 82\text{-}129}$	MTGAATAAENSATQLEFYRKALNFNVIGRYDPKIKQLLFHTPHASLYKWDFKKDEWNKLEYQ<br>GVLAIYLRDVSQNTNLLPVKTLSGKDIYNYGLIILNRINPDNFSMGIVPNSVVNKRKVFNAEED<br>TLNPLECVGVEVKDELVIIKNLKHEVYGIWIHTVSDRQNIYELIKYLLEYEPRDSCA
Dcp1$^{82\text{-}129}$	SPQEVDIFDSQNGSNNIQVNNGSDNSNRNSSGNGNSYKSNDSLTYNCG
Dcp2	MSLPLRHALENVTSVDRILEDLLVRFIINCPNEDLSSVERELFHFEEASWFYTDFIKLMNPTLPS<br>LKIKSFAQLIIKLCPLVWKWDIRVDEALQQFSKYKKSIPVRGAAIFNENLSKILLVQGTESDSWS<br>FPRGKISKDENDIDCCIREVKEEIGFDLTDYIDDNQFIERNIQGKNYKIFLISGVSEVFNFKPQVR<br>NEIDKIEWFDFKKISKTMYKSNIKYYLINSMMRPLSMWLRHQRQIKNEDQLKSYAEEQLKLLL<br>GITKEEQIDPGRELLNMLHTAVQANSNNNAVSNGQVPSSQELQHLKEHSGEHNQQKDQQS<br>SFSSQQQPSIFPSLSEPFANNKNVIPPTMPMANVFMSNPQLFATMNGQPFAPFPFMLPLTN<br>NSNSANPIPTPVPPNFNAPPNPMAFGVPNMHNLSGPAVSQPFSLPPAPLPRDSGYSSSSPG<br>QLLDILNSKKPDSNVQSSKKPKLKILQRGTDLNSIKQNNNDETAHSNSQALLDLLKKPTSSQKI<br>HASKPDTSFLPNDSVSGIQDAEYEDFESSSDEEVETARDERNSLNVDIGVNVMPSEKDSRRS<br>QKEKPRNDASKTNLNASAESNSVEWGPGKSSPSTQSKQNSSVGMQNKYRQEIHIGDSDAYE<br>VFESSSDEEDGKKLEELEQTQDNSKLISQDILKENNFQDGEVPHRDMPTESNKSINETVGLSS<br>TTNTVKKVPKVKILKRGETFASLANDKKAFDSSSNVSSSKDLLQMLRNPISSTVSSNQQSPKSQ<br>HLSGDEEIMMMLKRNSVSKPQNSEENASTSSINDANASELLGMLKQKEKDITAPKQPYNVD<br>SYSQKNSAKGLLNILKKNDSTGYPRTEGGPSSEMSTSMKRNDATNNQELDKNSTELLNYLKP<br>KPLNDGYENISNKDSSHELLNILHGNKNSSAFNNNVYATDGYSLASDNNENSSNKLLNMLQN<br>RSSAINEPNFDVRSNGTSGSNELLSILHRK

Dcp2$^{1-300}$	MSLPLRHALENVTSVDRILEDLLVRFIINCPNEDLSSVERELFHFEEASWFYTDFIKLMNPTLPS<br>LKIKSFAQLIIKLCPLVWKWDIRVDEALQQFSKYKKSIPVRGAAIFNENLSKILLVQGTESDSWS<br>FPRGKISKDENDIDCCIREVKEEIGFDLTDYIDDNQFIERNIQGKNYKIFLISGVSEVFNFKPQVR<br>NEIDKIEWFDFKKISKTMYKSNIKYYLINSMMRPLSMWLRHQRQIKNEDQLKSYAEEQLKLLL<br>GITKEEQIDPGRELLNMLHTAVQANSNNNAVSNGQVPSSQE
Dcp2$^{301-970}$	LQHLKEQSGEHNQQKDQQSSFSSQQQPSIFPSLSEPFANNKNVIPPTMPMANVFMSNPQL<br>FATMNGQPFAPFPFMLPLTNNSNSANPIPTPVPPNFNAPPNPMAFGVPNMHNLSGPAVS<br>QPFSLPPAPLPRDSGYSSSSPGQLLDILNSKKPDSNVQSSKKPKLKILQRGTDLNSIKQNNND<br>ETAHSNSQALLDLLKKPTSSQKIHASKPDTSFLPNDSVSGIQDAEYEDFESSSDEEVETARDER<br>NSLNVDIGVNVMPSEKDSRRSQKEKPRNDASKTNLNASAESNSVEWGPGKSSPSTQSKQN<br>SSVGMQNKYRQEIHIGDSDAYEVFESSSDEEDGKKLEELEQTQDNSKLISQDILKENNFQDGE<br>VPHRDMPTESNKSINETVGLSSTTNTVKKVPKVKILKRGETFASLANDKKAFDSSSNVSSSKDL<br>LQMLRNPISSTVSSNQQSPKSQHLSGDEEIMMMLKRNSVSKPQNSEENASTSSINDANASEL<br>LGMLKQKEKDITAPKQPYNVDSYSQKNSAKGLLNILKKNDSTGYPRTEGGPSSEMSTSMKRN<br>DATNNQELDKNSTELLNYLKPKPLNDGYENISNKDSSHELLNILHGNKNSSAFNNNVYATDG<br>YSLASDNNENSSNKLLNMLQNRSSAINEPNFDVRSNGTSGSNELLSILHRK
Dcp2^{H237A}	MSLPLRHALENVTSVDRILEDLLVRFIINCPNEDLSSVERELFHFEEASWFYTDFIKLMNPTLPS<br>LKIKSFAQLIIKLCPLVWKWDIRVDEALQQFSKYKKSIPVRGAAIFNENLSKILLVQGTESDSWS<br>FPRGKISKDENDIDCCIREVKEEIGFDLTDYIDDNQFIERNIQGKNYKIFLISGVSEVFNFKPQVR<br>NEIDKIEWFDFKKISKTMYKSNIKYYLINSMMRPLSMWLRAQRQIKNEDQLKSYAEEQLKLLL<br>GITKEEQIDPGRELLNMLHTAVQANSNNNAVSNGQVPSSQELQHLKEQSGEHNQQKDQQS<br>SFSSQQQPSIFPSLSEPFANNKNVIPPTMPMANVFMSNPQLFATMNGQPFAPFPFMLPLTN<br>NSNSANPIPTPVPPNFNAPPNPMAFGVPNMHNLSGPAVSQPFSLPPAPLPRDSGYSSSSPG<br>QLLDILNSKKPDSNVQSSKKPKLKILQRGTDLNSIKQNNNDETAHSNSQALLDLLKKPTSSQKI<br>HASKPDTSFLPNDSVSGIQDAEYEDFESSSDEEVETARDERNSLNVDIGVNVMPSEKDSRRS<br>QKEKPRNDASKTNLNASAESNSVEWGPGKSSPSTQSKQNSSVGMQNKYRQEIHIGDSDAYE<br>VFESSSDEEDGKKLEELEQTQDNSKLISQDILKENNFQDGEVPHRDMPTESNKSINETVGLSS<br>TTNTVKKVPKVKILKRGETFASLANDKKAFDSSSNVSSSKDLLQMLRNPISSTVSSNQQSPKSQ<br>HLSGDEEIMMMLKRNSVSKPQNSEENASTSSINDANASELLGMLKQKEKDITAPKQPYNVD<br>SYSQKNSAKGLLNILKKNDSTGYPRTEGGPSSEMSTSMKRNDATNNQELDKNSTELLNYLKP<br>KPLNDGYENISNKDSSHELLNILHGNKNSSAFNNNVYATDGYSLASDNNENSSNKLLNMLQN<br>RSSAINEPNFDVRSNGTSGSNELLSILHRK
Dcp2^{H237R}	MSLPLRHALENVTSVDRILEDLLVRFIINCPNEDLSSVERELFHFEEASWFYTDFIKLMNPTLPS<br>LKIKSFAQLIIKLCPLVWKWDIRVDEALQQFSKYKKSIPVRGAAIFNENLSKILLVQGTESDSWS<br>FPRGKISKDENDIDCCIREVKEEIGFDLTDYIDDNQFIERNIQGKNYKIFLISGVSEVFNFKPQVR<br>NEIDKIEWFDFKKISKTMYKSNIKYYLINSMMRPLSMWLRRQRQIKNEDQLKSYAEEQLKLLL<br>GITKEEQIDPGRELLNMLHTAVQANSNNNAVSNGQVPSSQELQHLKEQSGEHNQQKDQQS<br>SFSSQQQPSIFPSLSEPFANNKNVIPPTMPMANVFMSNPQLFATMNGQPFAPFPFMLPLTN<br>NSNSANPIPTPVPPNFNAPPNPMAFGVPNMHNLSGPAVSQPFSLPPAPLPRDSGYSSSSPG<br>QLLDILNSKKPDSNVQSSKKPKLKILQRGTDLNSIKQNNNDETAHSNSQALLDLLKKPTSSQKI<br>HASKPDTSFLPNDSVSGIQDAEYEDFESSSDEEVETARDERNSLNVDIGVNVMPSEKDSRRS<br>QKEKPRNDASKTNLNASAESNSVEWGPGKSSPSTQSKQNSSVGMQNKYRQEIHIGDSDAYE<br>VFESSSDEEDGKKLEELEQTQDNSKLISQDILKENNFQDGEVPHRDMPTESNKSINETVGLSS<br>TTNTVKKVPKVKILKRGETFASLANDKKAFDSSSNVSSSKDLLQMLRNPISSTVSSNQQSPKSQ

	HLSGDEEIMMMLKRNSVSKPQNSEENASTSSINDANASELLGMLKQKEKDITAPKQPYNVDSYSQKNSAKGLLNILKKNDSTGYPRTEGGPSSEMSTSMKRNDATNNQELDKNSTELLNYLKPKPLNDGYENISNKDSSHELLNILHGNKNSSAFNNNVYATDGYSLASDNNENSSNKLLNMLQNRSSAINEPNFDVRSNGTSGSNELLSILHRK
Dhh1	MGSINNNFNTNNNSNTDLDRDWKTALNIPKKDTRPQTDDVLNTRGNTFEDFYLKRELLMGIFEAGFEKPSPIQEEAIPVAITGRDILARAKNGTGKTAAFVIPTLEKVKPKLNKIRALIMVPTRELALQTSQVVRTLGKHCGISCMVTTGGTNLRDDILRLNETVHILVGTPGRVLDLASRRVADLSDCSLFIMDEADKMLSRDFKTIIEQILSFLPPTHQSLLFSATFPLTVKEFVVKHLHKPYDINVMEELTLKGITQYYAFVEERQELHCLNTLFSKLQINQAIIFCNSTNRVELLAKKITDLGYSCYYSHARMKQQERNKVFHEFRQGKVRTLVCSDLLTRGIDIQAVNVVINFDFPKTAETYLHRIGRSGRFGHLGLAINLINWNDRFNLYKIEQELGTEIAAIPATIDKSLYVAENDETVPVPFPIEQQSYHQQAIPQQQLPSQQQFAIPPQQHHPQFMVPPSHQQQQAYPPPQMPSQQGYPPQQEHFMAMPPGQSQPQY
Dhh1$^{25-425}$	ALNIPKKDTRPQTDDVLNTKGNTFEDFYLKRELLMGIFEAGFEKPSPIQEEAIPVAITGRDILARAKNGTGKTAAFVIPTLEKVKPKLNKIRALIMVPTRELALQTSQVVRTLGKHCGISCMVTTGGTNLRDDILRLNETVHILVGTPGRVLDLASRKVADLSDCSLFIMDEADKMLSRDFKTIIEQILSFLPPTHQSLLFSATFPLTVKEFVVKHLHKPYDINLMEELTLKGITQYYAFVEERQKLHCLNTLFSKLQINQAIIFCNSTNRVELLAKKITDLGYSCYYSHARMKQQERNKVFHEFRQGKVRTLVCSDLLTRGIDIQAVNVVINFDFPKTAETYLHRIGRSGRFGHLGLAINLINWNDRFNLYKIEQELGTEIAAIPATIDKSLYVAENDET
Dhh1$^{426-506}$	VPVPFPIEQQSYHQQAIPQQQLPSQQQFAIPPQQHHPQFMVPPSHQQQQAYPPPQMPSQQGY
Edc3	MSQFVGFGVQVELKDGKLIQGKIAKATSKGLTLNDVQFGDGGKSQAFKVRASRLKDLKVLTVASQSGKRKQQRQQQQQNDYNQNRGEHIDWQDDDVSKIKQREDFDFQRNLGMFNKKDVFAQLKQNDDILPENRLRGHNRKQTQLQQNNYQNDELVIPDAKKDSWNKISSRNEQSTHQSQPQQDAQDDLVLEDDEHEYDVDDIDDPKYLPITQSLNITHLIHSATNSPSINDKTRGTVINDKDQVLAKLGQMIISQSRSNSTSLPAANKQTTIRSKNTKQNIPMATPVQLLEMESITSEFFSINSAGLLENFAVNASFFLKQKLGGRARLRLQNSNPEPLVVILASDSNRSGAKALALGRHLCQTGHIRVITLFTCSQNELQDSMVKKQTDIYKKCGGKIVNSVSSLESAMETLNSPVEIVIDAMQGYDCTLSDLAGTSEVIESRIKSMISWCNKQRGSTKVWSLDIPNGFDAGSGMPDIFFSDRIEATGIICSGWPLIAINNLIANLPSLEDAVLIDIGIPQGAYSQRTSLRKFQNCDLFVTDGSLLLDL
Edc3$^{1-66}$	MSQFVGFGVQVELKDGKLIQGKIAKATSKGLTLNDVQFGDGGKSQAFKVRASRLKDLKVLTVASQS
Edc3$^{67-282}$	GKRKQQRQQQQQNDYNQNRGEHIDWQDDDVSKIKQQEDFDFQRNLGMFNKKDVFAQLKQNDDILPENRLRGHNRKQTQLQQNNYQNDELVIPDAKKDSWNKISSRNEQSTHQSQPQQDAQDDLVLEDDEHEYDVDDIDDPKYLPITQSLNITHLIHSATNSPSINDKTKGTVINDKDQVLAKLGQMIISQSRSNSTSLPAANKQTTIRSKNTKQNI
Edc3$^{283-551}$	PMATPVQLLEMESITSEFFSINSAGLLENFAVNASFFLKQKLGGRARLRLQNSNPEPLVVILASDSNRSGAKALALGRHLCQTGHIRVITLFTCSQNELQDSMVKKQTDIYKKCGGKIVNSVSSLESAMETLNSPVEIVIDAMQGYDCTLSDLAGTSEVIESRIKSMISWCNKQRGSTKVWSLDIPNGFDAGSGMPDIFFSDRIEATGIICSGWPLIAINNLIANLPSLEDAVLIDIGIPQGAYSQRTSLRKFQNCDLFVTDGSLLLDL
Lsm1-7	Lsm1:

	MSANSKDRNQSNQDAKRQQQNFPKKISEGEADLYLDQYNFTTTAAIVSSVDRKIFVLLRDGR MLFGVLRTFDQYANLILQDCVERIYFSEENKYAEEDRGIFMIRGENVVMLGEVDIDKEDQPLE AMERIPFKEAWLTKQKNDEKRFKEETHKGKKMARHGIVYDFHKSDMY<br>Lsm2:<br>MLFFSFFKTLVDQEVVVELKNDIEIKGTLQSVDQFLNLKLDNISCTDEKKYPHLGSVRNIFIRGS TVRYVYLNKNMVDTNLLQDATRREVMTERK<br>Lsm3:<br>METPLDLLKLNLDERVYIKLRGARTLVGTLQAFDSHCNIVLSDAVETIYQLNNEELSESERRCE MVFIRGDTVTLISTPSEDDDGAVEI<br>Lsm4:<br>MLPLYLLTNAKGQQMQIELKNGEIIQGILTNVDNWMNLTLSNVTEYSEESAINSEDNAESSK A<br>VKLNEIYIRGTFIKFIKLQDNIIDKVKQQINSNNNSNSNGPGHKRYYNNRDSNNNRGNYNRR N<br>NNNGNSNRRPYSQNRQYNNSNSSNINNSINSINSNNQNMNNGLGGSVQHHFNSSSPQKV EF<br>Lsm5:<br>MSLPEILPLEVIDKTINQKVLIVLQSNREFEGTLVGFDDFVNVILEDAVEWLIDPEDESRNEKV MQHHGRMLLSGNNIAILVPGGKKTPTEAL<br>Lsm6:<br>MSGKASTEGSVTTEFLSDIIGKTVNVKLASGLLYSGRLESIDGFMNVALSSATEHYESNNNKLL NKFNSDVFLRGTQVMYISEQKI<br>Lsm7:<br>MHQQHSKSENKPQQQRKKFEGPKREAILDLAKYKDSKIRVKLMGGKLVIGVLKGYDQLMNL VLDDTVEYMSNPDDENNTELISKNARKLGLTVIRGTILVSLSSAEGSDVLYMQK
Pat1	MSFFGLENSGNARDGPLDFEESYKGYGEHELEENDYLNDETFGDNVQVGTDFDFGNPHSSG SSGNAIGGNGVGATARSYVAATAEGISGPRTDGTAAAGPLDLKPMESLWSTAPPPAMAPSP QSTMAPAPAPQQMAPLQPILSMQDLERQQRQMQQQFMNFHAMGHPQGLPQGPPQQ QFPMQPASGQPGPSQFAPPPPPPGVNVNMNHMPMGPVQVPVQASPSPIGMSNTPSPGP VVGATNMPLQSGRRSKRDVSPEEQRRLQIRHARVEKILKYSGIMTPRDKDFITRYQLSHIVTE DPYNEDFYFQVYKIIQRGGITSESNKGLIARAYLEHSGHRLGGRYKRTDIALQRMQSQVEKAV TVAKERPSKLKDQQAAAGNSSQDNKQANTVLGKISSTLNSKNPRRQLQIPRQQPSDPDALK DVTDSLTNVDLASSGSSSTGSSAAAVASKQRRRSSYAFNNGNGATNLNKSGGKKFILELIETV YEEILDLEANLRNGQQTDSTAMWEALHIDDSSYDVNPFISMLSFDKGIKIMPRIFNFLDKQQK LKILQKIFNELSHLQIIILSSYKTTPKPTLTQLKKVDLFQMIILKIIVSFLSNNSNFIEIMGLLLQLIRN NNVSFLTTSKIGLNLITILISRAALIKQDSSRSNILSSPEISTWNEIYDKLFTSLESKIQLIFPPREYN DHIMRLQNDKFMDEAYIWQFLASLALSGKLNHQRIIIDEVRDEIFATINEAETLQKKEKELSVL PQRSQELDTELKSIIYNKEKLYQDLNLFLNVMGLVYRDGEISELK
Pat1$^{1-240}$	MSFFGLENSGNARDGPLDFEESYKGYGEHELEENDYLNDETFGDNVQVGTDFDFGNPHSSG SSGNAIGGNGVGATARSYVAATAEGISGPRTDGTAAAGPLDLKPMESLWSTAPPPAMAPSP QSTMAPAPAPQQMAPLQPILSMQDLERQQRQMQQQFMNFHAMGHPQGLPQGPPQQ QFPMQPASGQPGPSQFAPPPPPPGVNVNMNQMPMGPVQVPVQASPSPIGMSNTPSPG PVVGAT

Pat1$^{241-422}$	KMPLQSGRRSKRDLSPEEQRRLQIRHAKVEKILKYSGLMTPRDKDFITRYQLSQIVTEDPYNED<br>FYFQVYKIIQRGGITSESNKGLIARAYLEHSGHRLGGRYKRTDIALQRMQSQVEKAVTVAKER<br>PSKLKDQQAAAGNSSQDNKQANTVLGKISSTLNSKNPRRQLQIPRQQPSDPDALK
Pat1$^{423-796}$	DVTDSLTNVDLASSGSSSTGSSAAAVASKQRRRSSYAFNNGNGATNLNKSGGKKFILELIETV<br>YEEILDLEANLRNGQQTDSTAMWEALHIDDSSYDVNPFISMLSFDKGIKIMPRIFNFLDKQQK<br>LKILQKIFNELSHLQIIILSSYKTTPKPTLTQLKKVDLFQMIILKIIVSFLSNNSNFIEIMGLLLQLIRN<br>NNVSFLTTSKIGLNLITILISRAALIKQDSSRSNILSSPEISTWNEIYDKLFTSLESKIQLIFPPREYN<br>DHIMRLQNDKFMDEAYIWQFLASLALSGKLNHQRIIIDEVRDEIFATINEAETLQKKEKELSVL<br>PQRSQELDTELKSIIYNKEKLYQDLNLFLNVMGLVYRDGEISELK
Xrn1	MGIPKFFRYISERWPMILQLIEGTQIPEFDNLYLDMNSILHNCTHGNDDDVTKRLTEEEVFAKI<br>CTYIDHLFQTIKPKKIFYMAIDGVAPRAKMNQQRARRFRTAMDAEKALKKAIENGDEIPKGE<br>PFDSNSITPGTEFMAKLTKNLQYFIHDKISNDSKWREVQIIFSGHEVPGEGEHKIMNFIRHLKS<br>QKDFNQNTRHCIYGLDADLIMLGLSTHGPHFALLREEVTFGRRNSEKKSLEHQNFYLLHLSLL<br>REYMELEFKEIADEMQFEYNFERILDDFILVMFVIGNDFLPNLPDLHLNKGAFPVLLQTFKEAL<br>LHTDGYINEHGKINLKRLGVWLNYLSQFELLNFEKDDIDVEWFNKQLENISLEGERKRQRVGK<br>KLLVKQQKKLIGSIKPWLMEQLQEKLSPDLPDEEIPTLELPKDLDMKDHLEFLKEFAFDLGLFIT<br>HSKSKGSYSLKMDLDSINPDETEEEFQNRVNSIRKTIKKYQNAIIVEDKEELETEKTIYNERFER<br>WKHEYYHDKLKFTTDSEEKVRDLAKDYVEGLQWVLYYYYRGCPSWSWYYPHHYAPRISDLA<br>KGLDQDIEFDLSKPFTPFQQLMAVLPERSKNLIPPAFRPLMYDEQSPIHDFYPAEVQLDKNGK<br>TADWEAVVLISFVDEKRLIEAMQPYLRKLSPEEKTRNQFGKDLIYSFNPQVDNLYKSPLGGIFS<br>DIEHNHCVEKEYITIPLDSSEIRYGLLPNAKLGAEMLAGFPTLLSLPFTSSLEYNETMVFQQPSK<br>QQSMVLQITDIYKTNNVTLEDFSKRHLNKVIYTRWPYLRESKLVSLTDGKTIYEYQESNDKKKF<br>GFITKPAETQDKKLFNSLKNSMLRMYAKQKAVKIGPMEAIATVFPVTGLVRDSDGGYIKTFSP<br>TPDYYPLQLVVESVVNEDERYKERGPIPIEEEFPLNSKVIFLGDYAYGGETTIDGYSSDRRLKITV<br>EKKFLDSEPTIGKERLQMDHQAVKYYPSYIVSKNMHLHPLFLSKITSKFMITDATGKHINVGIP<br>VKFEARHQKVLGYARRNPRGWEYSNLTLNLLKEYRQTFPDFFFRLSKVGNDIPVLEDLFPDTS<br>TKDAMNLLDGIKQWLKYVSSKFIAVSLESDSLTKTSIAAVEDHIMKYAANIEGHERKQLAKVP<br>REAVLNPRSSFALLRSQKFDLGDRVVYIQDSGKVPIFSKGTVVGYTTLSSSLSIQVLFDHEIVAG<br>NNFGGRLRTNRGLGLDASFLLNITNRQFIYHSKASKKALEKKKQSNNRNNNTKTAHKTPSKQ<br>QSEEKLRKERAHDLLNFIKKDTNEKNSESVDNKSMGSQKDSKPAKKVLLKRPAQKSSENVQV<br>DLANFEKAPLDNPTVAGSIFNAVANQYSDGIGSNLNIPTPPHPMNVVGGPIPGANDVADVG<br>LPYNIPPGFMTHPNGLHPLHPHQMPYPNMNGMSIPPPAPHGFGQPISFPPPPPMTNVSDQ<br>GSRIVVNEKESQDLKKFINGKQHSNGSTIGGETKNSRKGEIKPSSGTNSTECQSPKSQSNAAD<br>RDNKKDEST
Not1$^{754-1000}$	MAPKERSRPVQEMIPLKFFAVDEVSCQINQEGAPKDVVEKVLFVLNNVTLANLNNKVDELKK<br>SLTPNYFSWFSTYLVTQRAKTEPNYHDLYSKVIVAMGSGLLHQFMVNVTLRQLFVLLSTKDE<br>QAIDKKHLKNLASWLGCITLALNKPIKHKNIAFREMLIEAYKENRLEIVVPFVTKILQRASESKIF<br>KPPNPWTVGILKLLIELNEKANWKLSLTFEVEVLLKSFNLTTKSLKPSNFINTPEVIETLSG
EML4-ALK V3	MDGFAGSLDDSISAASTSDVQDRLSALESRVQQQEDEITVLKAALADVLRRLAISEDHVASVK<br>KSVSSKGQPSPRAVIPMSCITNGSGANRKPSHTSAVSIAGKETLSSAAKSGTEKKKEKPQGQR<br>EKKEESHSNDQSPQIRASPSPQPSSQPLQIHRQTPESKNATPTKSIKRPSPAEKSHNSWENSD<br>DSRNKLSKIPSTPKLIPKVTKTADKHKDVIINQVYRRKHQELQAMQMELQSPEYKLSKLRTSTI<br>MTDYNPNYCFAGKTSSISDLKEVPRKNITLIRGLGHGAFGEVYEGQVSGMPNDPSPLQVAVK

	TLPEVCSEQDELDFLMEALIISKFNHQNIVRCIGVSLQSLPRFILLELMAGGDLKSFLRETRPRPS QPSSLAMLDLLHVARDIACGCQYLEENHFIHRDIAARNCLLTCPGPGRVAKIGDFGMARDIYR ASYYRKGGCAMLPVKWMPPEAFMEGIFTSKTDTWSFGVLLWEIFSLGYMPYPSKSNQEVLE FVTSGGRMDPPKNCPGPVYRIMTQCWQHQPEDRPNFAIILERIEYCTQDPDVINTALPIEYG PLVEEEEKVPVRPKDPEGVPPLLVSQQAKREEERSPAAPPPLPTTSSGKAAKKPTAAEVSVRV PRGPAVEGGHVNMAFSQSNPPSELHRVHGSRNKPTSLWNPTYGSWFTEKPTKKNNPIAKK EPHERGNLGLEGSCTVPPNVATGRLPGASLLLEPSSLTANMKEVPLFRLRHFPCGNVNYGYQ QQGLPLEAATAPGAGHYEDTILKSKNSMNQPGP
SIM-3R	KVDVIDLTIESSSDEEEDPPAKRGGSGGSGGSGGSKVDVIDLTIESSSDEEEDPPAKRGGSGGS GGSGGSKVDVIDLTIESSSDEEEDPPAKR

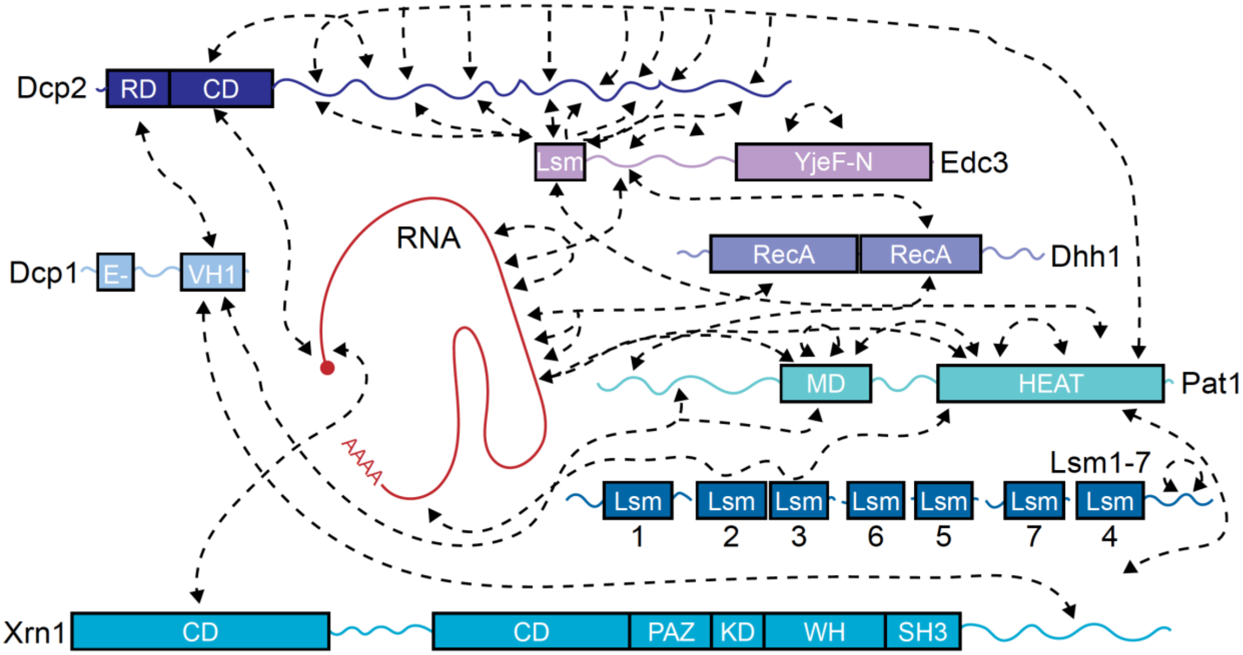

Figure S1. Multivalent interactions between P-body proteins and RNA.
Schematic of P-body proteins and RNA as in Figure 1A. Boxes indicate structured domains, and squiggly lines denote intrinsically disordered regions (IDRs). Domain abbreviations follow previously established nomenclature: Dcp1, Ena/VASP Homology 1 (EVH1)(18); Dcp2, Regulatory Domain (RD) and Catalytic Domain (CD)(19); Dhh1, RecA-like domain (RecA)(24); Edc3, Like Sm (Lsm)(25), YjeF N terminal protein domain (YjeF-N)(7); Lsm1-7, Like Sm (Lsm)(8); Pat1, Middle Domain (MD), Huntington-EF3-PP2A-TOR1 like domain super helix (HEAT)(8); Xrn1, Catalytic Domain (CD), Piwi-Argonaute-Zwille domain (PAZ), Kyrpides-Ouzounis-Woese domain (KD), Winged Helix(WH), SRC Homology domain 3 like domain (SH3) (13). Gray dotted lines denote interactions. References for interactions are: Dcp1 (9, 19, 26), Dcp2 (10, 19, 25-27), Dhh1 (11, 24, 26), Edc3 (7, 11, 25, 27, 28), Lsm1-7 (8, 26, 29), Pat1 (8, 10, 26, 30), and Xrn1 (9, 13). Multivalent interactions between P-body proteins and RNA involve both structured domains and IDRs. Evidence suggests Dhh1 oligomerizes (21, 31, 32), though it is not clear which domain(s) mediate oligomerization so this interaction was not depicted with arrows.

See also Fig. 1A, and S2-S3.

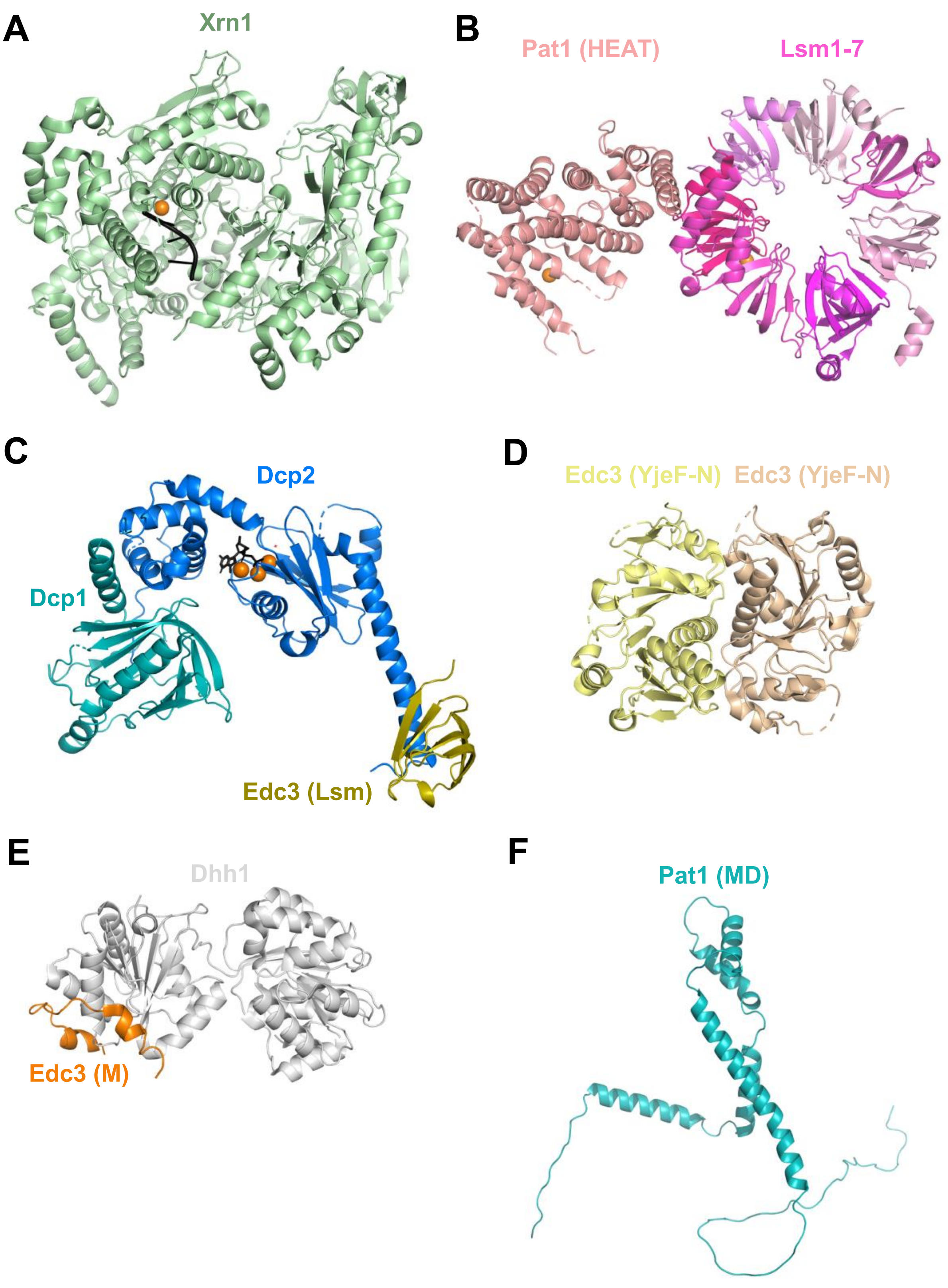
A
Xrn1
B
Pat1 (HEAT)
Lsm1-7
C
Dcp2
Dcp1
Edc3 (Lsm)
D
Edc3 (YjeF-N)
Edc3 (YjeF-N)
E
Dhh1
Edc3 (M)
F
Pat1 (MD)

Figure S2. Structured domains within P-body proteins.
Structures for:
(A) Xrn1 CD, PAZ, KD, WH, and SH3 domains (13).
(B) Pat1 HEAT domain and Lsm1-7 (8).
(C) Dcp1, Dcp2 RD and CD domains, and Edc3 Lsm domain (12).
(D)Edc3 YjeF-N domain dimer (7).
(E) Dhh1 RecA domains and Edc3 M domain (11).
(F) Structural model for the middle domain (MD) of Pat1 ($Pat1^{241\text{-}422}$) was generated using AlphaFold (16, 17). Secondary structure and disorder predictions are in agreement that Pat1 MD contains structured elements (Figure S3). However, these α-helices likely lack a defined and stable stereospecific three-dimensional structure due to: the lack of extensive tertiary contacts within the MD domain or to the HEAT domain, the flexible linkers between the α-helices, and consequently the low confidence of this model for the regions outside of the α-helices. See the following website for further details: https://alphafold.ebi.ac.uk/entry/P25644.

Metal ions are colored orange in Xrn1 (Mg^{2+}), Dcp2 (Mg^{2+}), and Pat1/Lsm1-7 (Co^{2+}) structures, and RNA (oligo)nucleotides are colored black in Xrn1 and Dcp2 structures. The structured domains of P-body proteins are interaction surfaces that increase the valency within the P-body networks. Note that Pat1 HEAT domain also dimerizes (10), Pat1 N domain binds to Dhh1 in a similar position as Edc3 M domain (11), and Xrn1 C domain binds to Dcp1 (9); these structures have been omitted for brevity.

See also Fig. 1A, S3, and Methods.

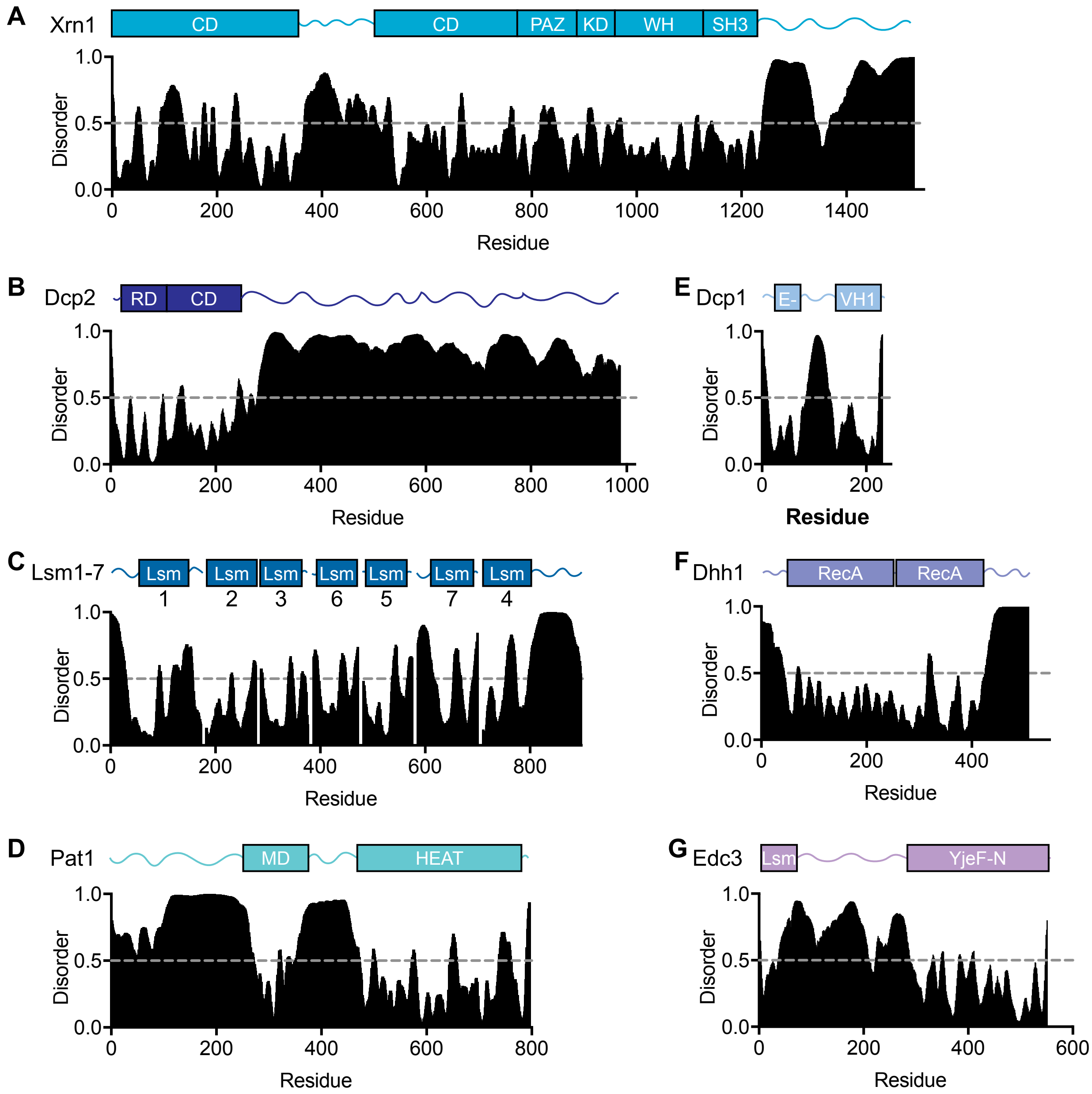

Figure S3. Intrinsically disordered regions within P-body proteins.
Disorder score was calculated for P-body proteins using Disprot VSL2 (15). Higher scores suggest disordered regions and lower scores suggest structured domains.

See also Fig. 1A and S2.

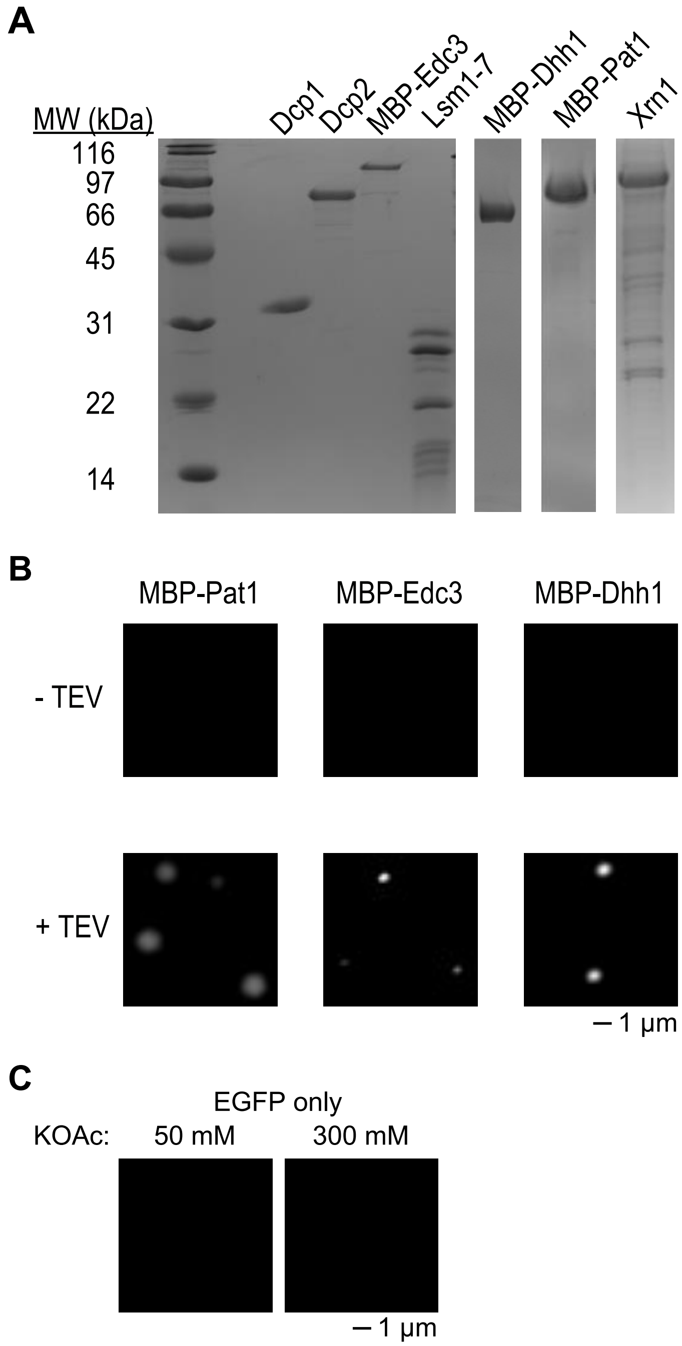

Figure S4. Purified P-body proteins and TEV cleavage initiation of condensate formation.
(A) SDS PAGE gel of purified P-body proteins used in this study. Proteins were purified using a four step purification process: Ni^{2+} affinity, amylose affinity, ion exchange, and size exclusion columns. Dhh1, Edc3, and Pat1 required the MBP tag for purification to prevent loss of protein. See methods for details.
(B) Using the purified proteins, TEV cleavage of the MBP tag was used to initiate phase separation. TEV was added at a 50:1 P-body protein:TEV ratio as higher amounts of TEV

perturbed condensate formation and maturation. Data shown here for EGFP-fusions for all proteins.
(C) EGFP (1 μM) does not form condensates at 50 mM nor 300 mM KOAc, pH 7. This suggests that condensates are formed via P-body protein interactions rather than by EGFP.

See also Fig. 1B-1C.

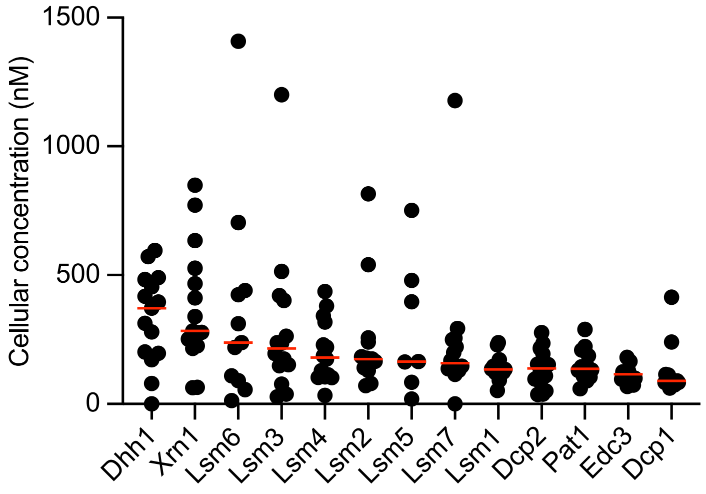

Figure S5. Estimated cellular concentrations of P-body proteins.
The absolute number of proteins in a Saccharomyces cerevisiae cell were obtained from Ho *et al.* (33). Briefly, Ho et al. combined mass spectrometry and fluorescent microscopy studies with quantitative proteome wide data. We converted the number of proteins per cell to concentration using the following assumptions: (i) cell volume of 62 μm^3; (ii) P-body proteins were not restricted from any organelle as the cytoplasm makes up the majority of the cell volume, and most P-body proteins also reside in the nucleus which is the largest compartment after the cytoplasm (34). Each dot is from a single quantitative report and the red line indicates the median. The median cellular concentration estimates for P-body proteins range from 370 to 90 µM.

See also Fig. 1D.

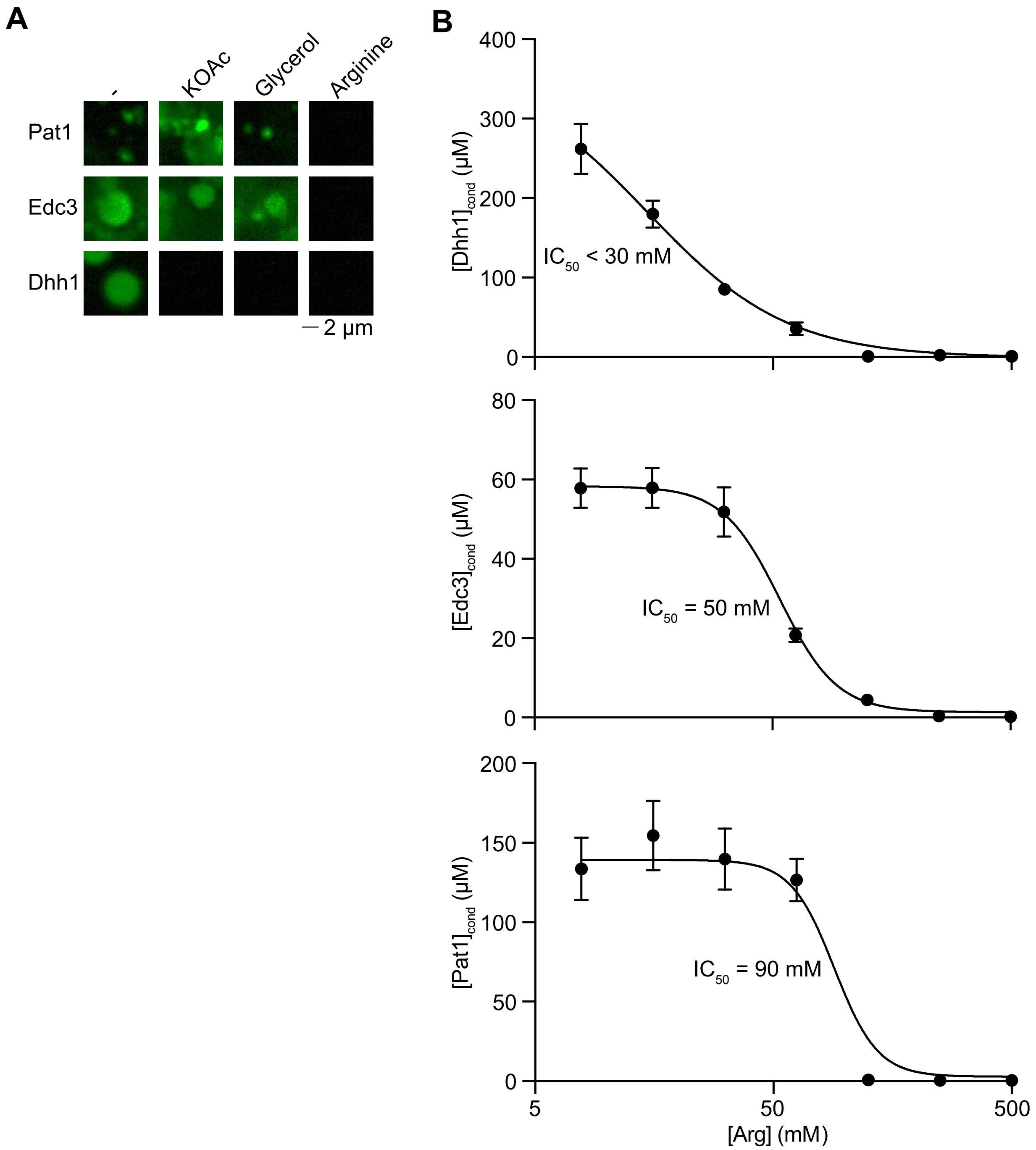

Figure S6. Disruption of homotypic condensates.

(A) Representative micrographs for Pat1-EGFP (top), Edc3-EGFP (middle), and Dhh1-EGFP (bottom). All reactions were conducted at pH 7 with the following added: 50 mM KOAc buffer (1st column), 300 mM KOAc (2nd column), 10% glycerol (3rd column), and 100 mM arginine (4th column). All homotypic condensates were prevented by arginine, and all added molecules prevented Dhh1 condensates.

(B) Titration of arginine against Dhh1 (top), Edc3 (middle), and Pat1 (bottom) homotypic condensates. Arginine was added before condensate formation was initiated.

See also Fig. 1E.

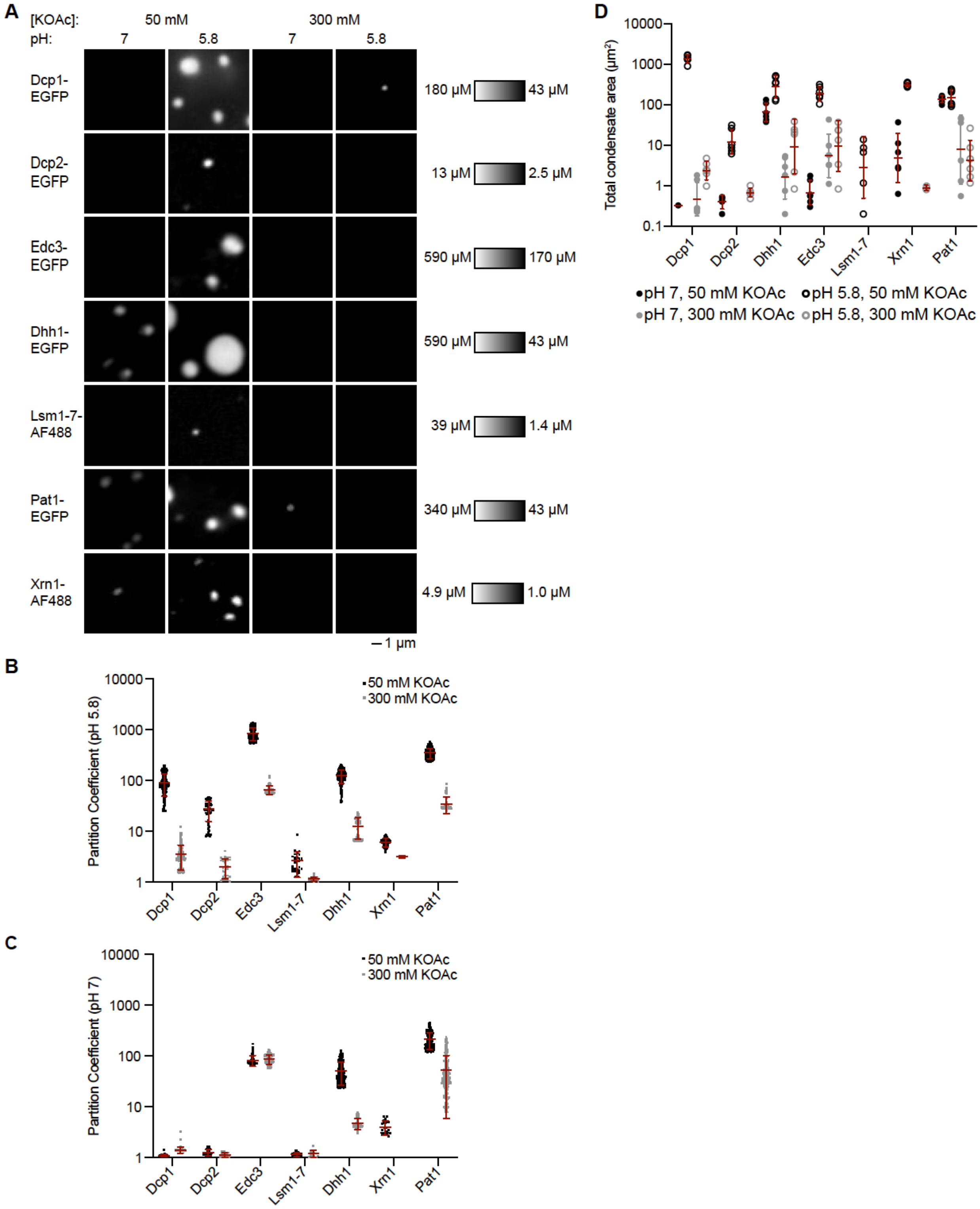

A
[KOAc]:
50 mM
300 mM
pH:
7
5.8
7
5.8
Dcp1-EGFP
Dcp2-EGFP
Edc3-EGFP
Dhh1-EGFP
Lsm1-7-AF488
Pat1-EGFP
Xrn1-AF488
180 μM
43 μM
13 μM
2.5 μM
590 μM
170 μM
590 μM
43 μM
39 μM
1.4 μM
340 μM
43 μM
4.9 μM
1.0 μM
1 μm
B
Partition Coefficient (pH 5.8)
50 mM KOAc
300 mM KOAc
Dcp1
Dcp2
Edc3
Lsm1-7
Dhh1
Xrn1
Pat1
C
Partition Coefficient (pH 7)
50 mM KOAc
300 mM KOAc
D
Total condensate area (μm²)
pH 7, 50 mM KOAc
pH 5.8, 50 mM KOAc
pH 7, 300 mM KOAc
pH 5.8, 300 mM KOAc

Figure S7. Partition coefficients for individual P-body proteins at pH 7 and 5.8, and 50 and 300 mM KOAc.
(A) Representative fluorescent micrographs for individual P-body proteins in all buffer conditions. Contrast is consistent for individual proteins across rows and is indicated on the right.
(B) Partition coefficients for individual P-body proteins at pH 5.8 for 50 mM KOAc (black) and 300 mM KOAc (gray). Proteins labeled with the same fluorophore as indicated in (A).
(C) Partition coefficients for individual P-body proteins at pH 7 with 50 mM KOAc (black) and 300 mM KOAc (gray). Proteins labeled with the same fluorophore as indicated in (A).
(D) Total condensate area for individual P-body proteins in all conditions. Individual data points represent the sum from one micrograph. Three micrographs were examined from each of two individual experiments for a total of six micrographs. Red bars indicate mean and standard deviation. Proteins labeled with the same fluorophore as indicated in (A).

All individual P-body proteins exhibit enhanced partitioning into the condensed phase at pH 5.8, with the magnitude of the effect being most dramatic for Dcp1, Dcp2, and Edc3. Interestingly, pH 5.8 sensitizes Pat1 and especially Edc3 to electrostatic disruption with 300 mM KOAc.

See also Fig. 2A-B.

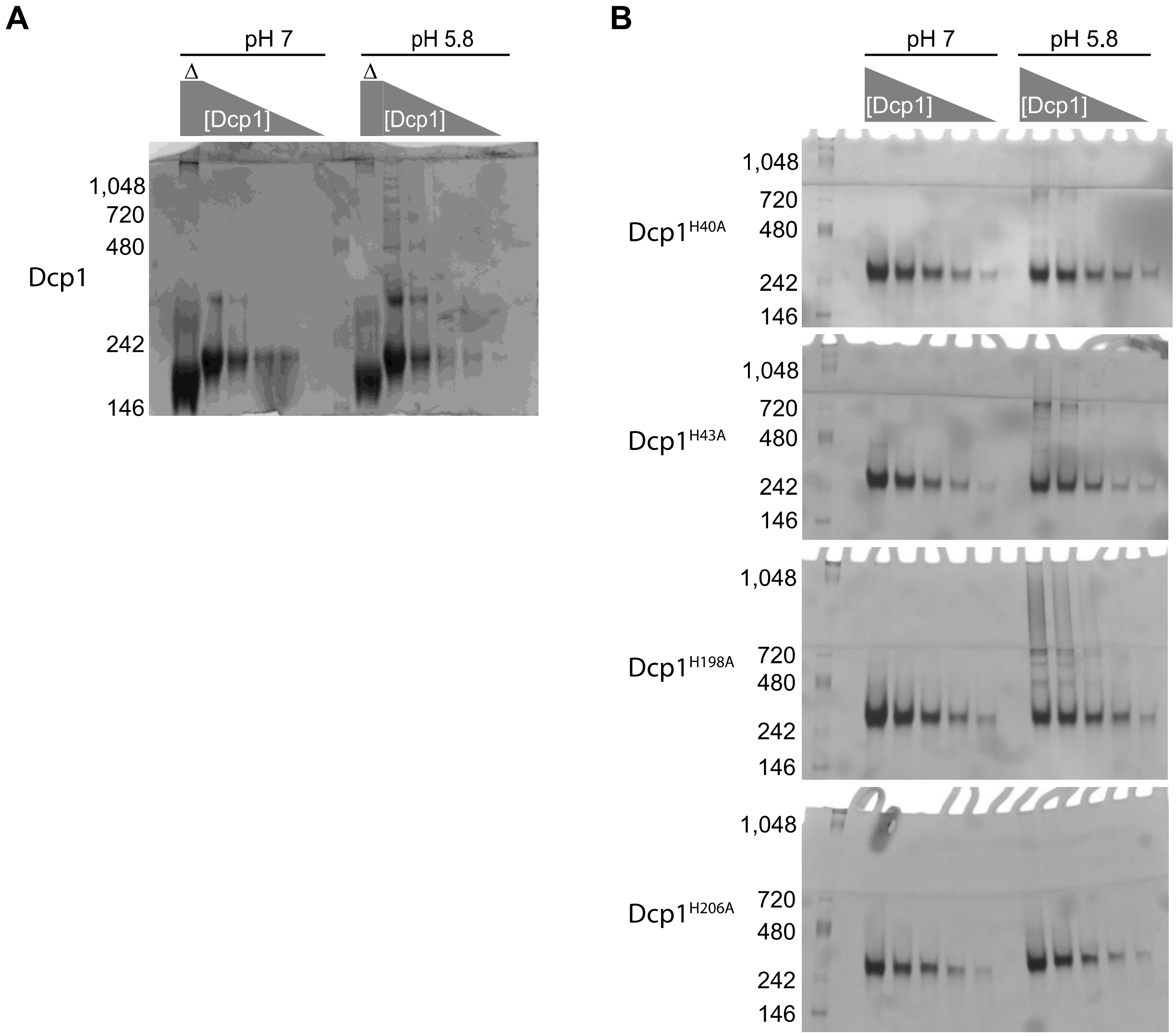

Figure S8. H206A mutation disrupts Dcp1 oligomerization.
(A) Native gel examining the oligomerization of Dcp1 at pH 7 (left) and pH 5.8 (right). Dcp1 concentration was diluted in twofold increments from 10 to 0.6 µM. The first lane has 10 µM Dcp1, and was incubated with SDS (Laemmli's loading buffer) at 100 °C for 5 minutes before loading.
(B) Native gels examining oligomerization of Dcp1 histidine mutants. For histidine mutants the concentration range was from 5 to 0.3 µM.

Mutation of histidine 206 to alanine results in the most dramatic inhibition of Dcp1 LLPS and oligomerization at pH 5.8. The histidine 206 residues interact with each other at the dimer interface suggesting that this contact is important for Dcp1 LLPS and oligomerization. H40A and H43A mutants have a slight disruption in both assays, whereas mutation of histidine 198 has no impact. These data suggest that histidine 206 is particularly important for the pH-sensitivity of Dcp1 oligomerization and homotypic LLPS.

See also Fig. 2C-E .

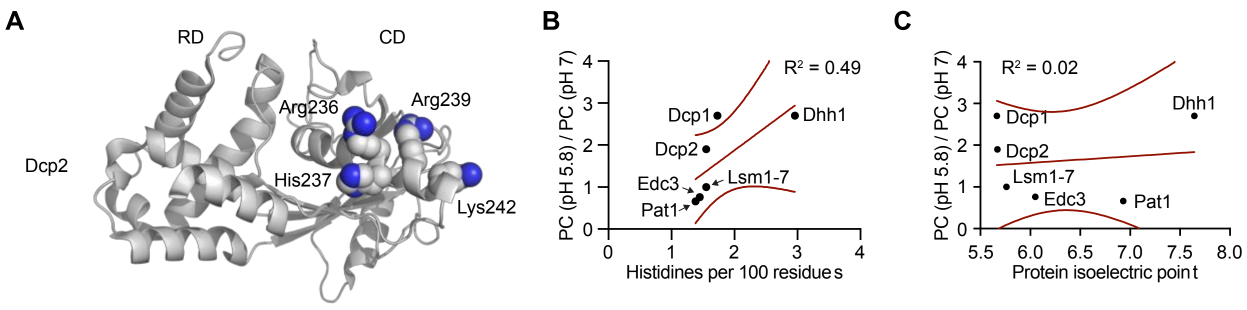

Figure S9. Dcp2 structured domains and histidine density in P-body proteins.
(A) Homology model of Dcp2 structured regulatory and catalytic domains with residues within previously identified RNA-binding region highlighted (19).
(B) Ratio of partition coefficients (pH 5.8 / pH 7 in 300 mM KOAc) versus the frequency of histidine residues (number of histidine residues per 100 amino acids) for all P-body proteins that were tested. Solid line indicates linear fit and dotted lines indicate 95% confidence interval.
(C) Ratio of partition coefficients (pH 5.8 / pH 7 in 300 mM KOAc) versus the protein isoelectric point for all P-body proteins that were tested. Solid line indicates linear fit and dotted lines indicate 95% confidence interval.

There is a reasonable correlation between histidine density and pH-sensitive condensate formation (R^2 = 0.49) that is enhanced if Dcp1 is not considered (Figure 2I: R^2 = 0.75). In the outlier, Dcp1, a single dominant histidine residue (H206) controls pH-sensitive condensate formation. In contrast, the protein isoelectric point does not correlate with pH-sensitive condensate formation (R2 = 0.02). These data suggest that multiple pH-sensitive histidine residues contribute to homotypic condensate formation and are distributed throughout Dcp2 and the other P-body proteins.

See also Fig. 2C-I.

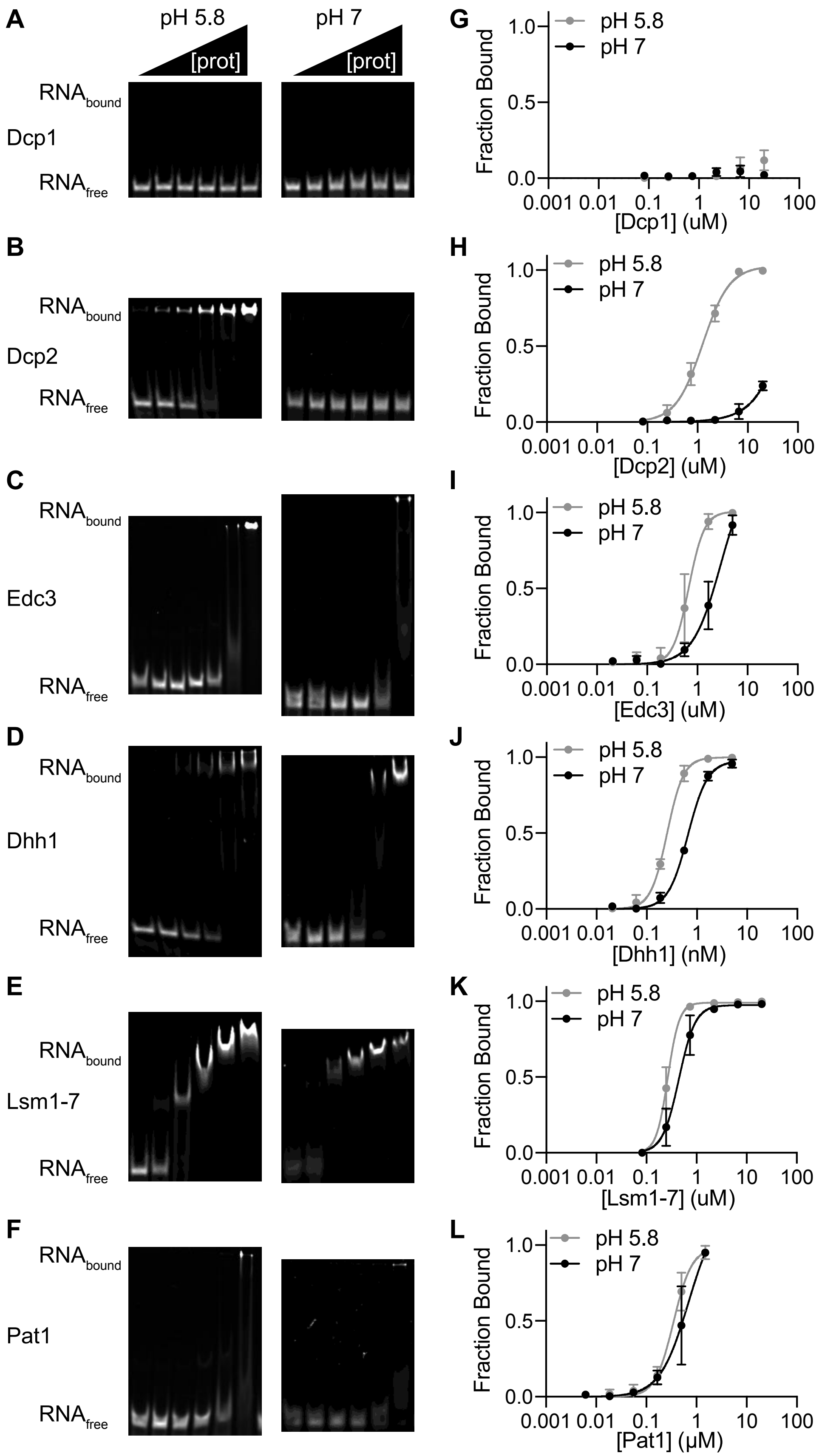
A
pH 5.8
pH 7
[prot]
[prot]
RNA bound
Dcp1
RNA free
B
RNA bound
Dcp2
RNA free
C
RNA bound
Edc3
RNA free
D
RNA bound
Dhh1
RNA free
E
RNA bound
Lsm1-7
RNA free
F
RNA bound
Pat1
RNA free
G
Fraction Bound
pH 5.8
pH 7
[Dcp1] (uM)
H
[Dcp2] (uM)
I
[Edc3] (uM)
J
[Dhh1] (nM)
K
[Lsm1-7] (uM)
L
[Pat1] (μM)
0.0
0.5
1.0
0.001 0.01 0.1 1 10 100

Figure S10. Acidic pH promotes P-body protein binding to RNA.
(A-F) EMSAs for P-body proteins binding to RPL41A RNA at pH 5.8 (left) and pH 7 (right). Gels are representative examples of three replicate experiments.
(G-L) Quantitation of K_D values for P-body proteins binding to RNA. Data points and error bars correspond to the mean and standard deviation from three replicate experiments.

See also Table S2; Fig. 3A-C and S11.

RPL41A

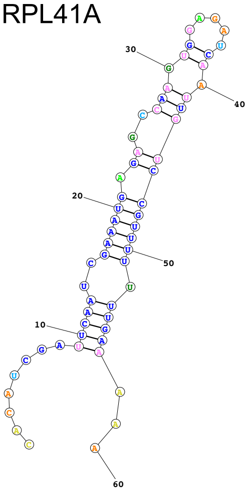

RNA10

UGGCAUUUCG

Total RNA
~ 80% rRNA
~ 15% tRNA
~ 5% mRNA

MFA2

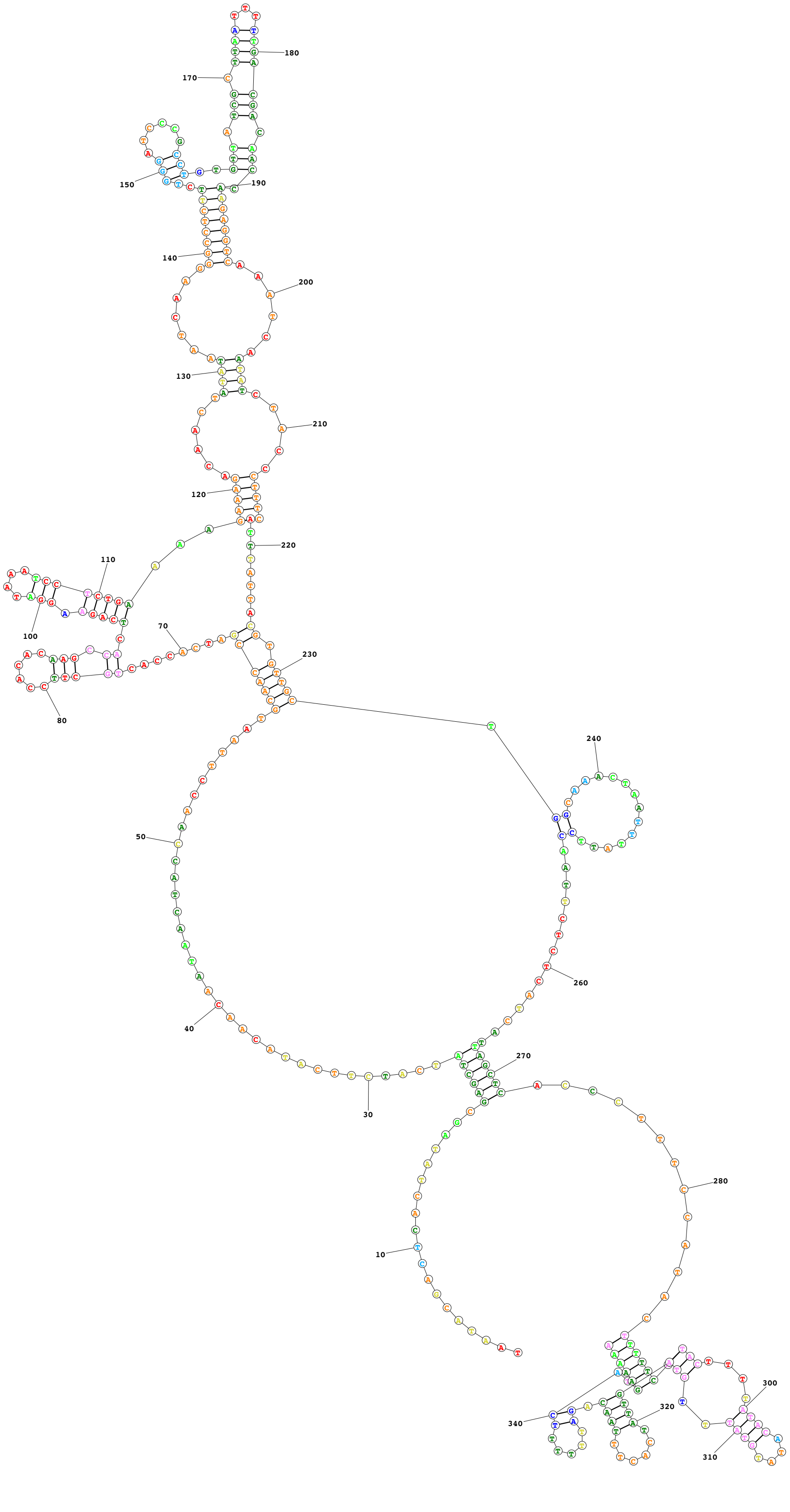

Figure S11. Types of RNA used in this study.

Portions of RPL41A covering the 5' UTR and translational start site, and the 3'UTR and start of the polyA tail were combined in this 60 nucleotide RNA. RNA10 is a small single-stranded RNA previously used in RNA helicase studies (35). Total RNA contains more diverse RNA types and features (length, secondary structure, etc.). Yeast Mating Factor A (MFA2) is a full-length mRNA (348 nucleotides) that localizes to cellular P bodies (36). Predicted secondary structures for RPL41A and MFA2 from the RNAstructure website is shown (37).

See also Fig. 3A-C, 5B, S10, S19, S22, and S23.

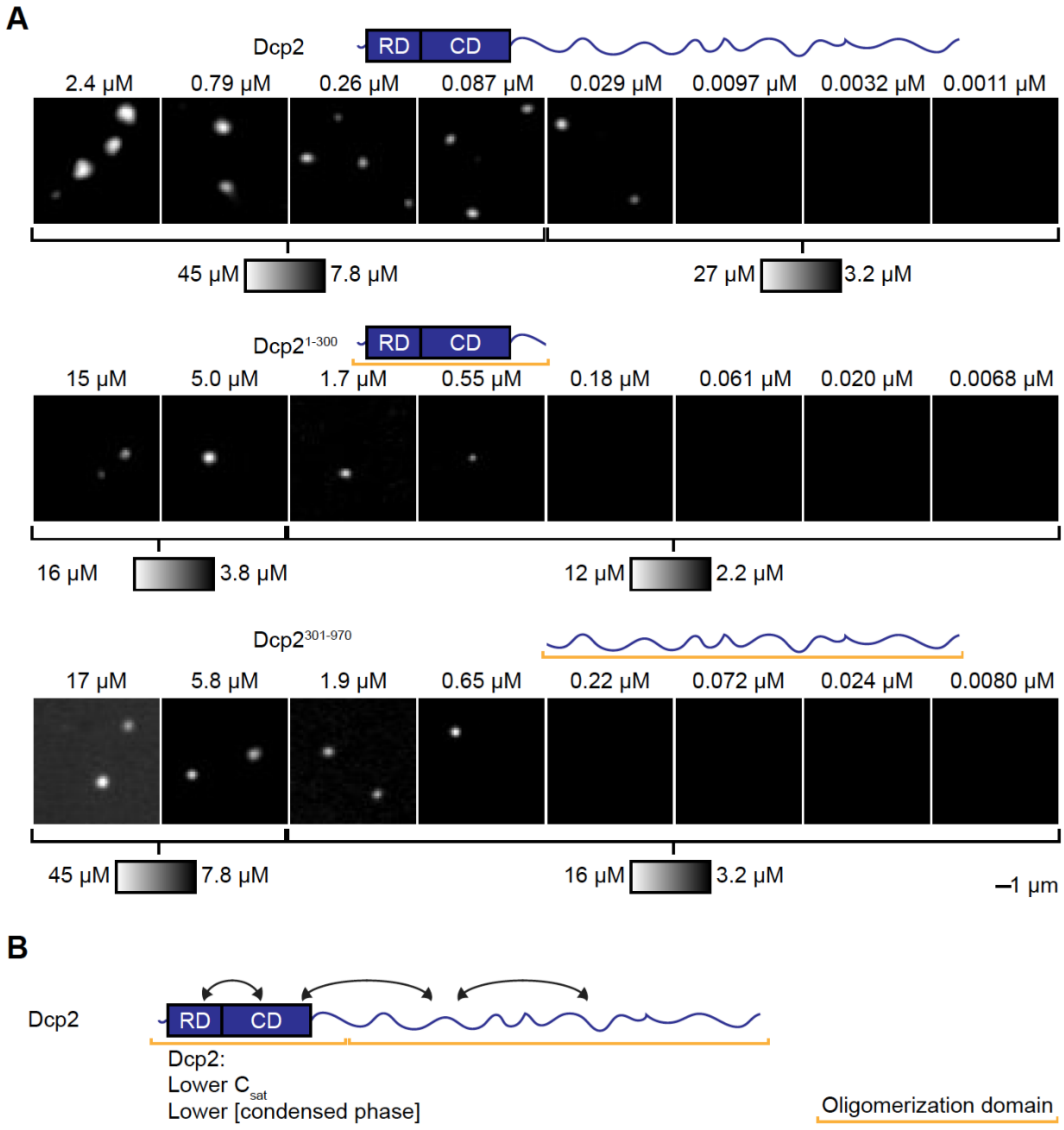

Figure S12. Titration of Dcp2 truncations.

(A) Representative micrographs for titration of Dcp2 and truncations. Total concentration of protein and contrast are labeled above and below the micrographs, respectively. All are EGFP fusion proteins.

(B) Cartoon schematic representing: Dcp2 has a lower saturation concentration (C_{sat}) than either truncation, suggesting that the structured N-terminal domains and disordered C-terminal regions

synergize to drive C_{sat} lower in full-length Dcp2. The condensate concentration for $Dcp2^{301\text{-}970}$ is higher, albeit at higher total protein concentrations.

See also Fig. 4A and 4K.

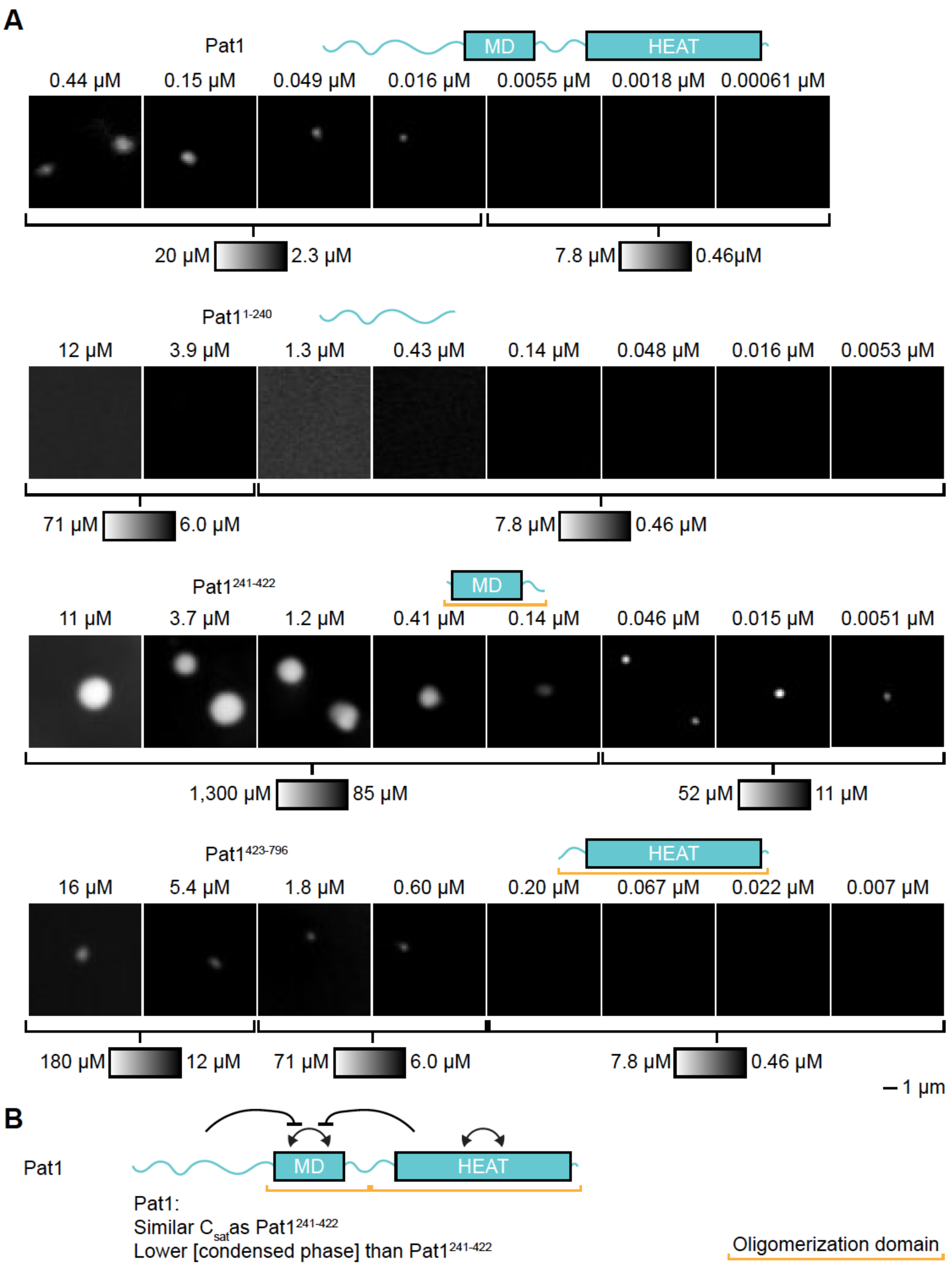
A
Pat1
MD
HEAT
0.44 μM
0.15 μM
0.049 μM
0.016 μM
0.0055 μM
0.0018 μM
0.00061 μM
20 μM
2.3 μM
7.8 μM
0.46μM
Pat1 1-240
12 μM
3.9 μM
1.3 μM
0.43 μM
0.14 μM
0.048 μM
0.016 μM
0.0053 μM
71 μM
6.0 μM
7.8 μM
0.46 μM
Pat1 241-422
MD
11 μM
3.7 μM
1.2 μM
0.41 μM
0.14 μM
0.046 μM
0.015 μM
0.0051 μM
1,300 μM
85 μM
52 μM
11 μM
Pat1 423-796
HEAT
16 μM
5.4 μM
1.8 μM
0.60 μM
0.20 μM
0.067 μM
0.022 μM
0.007 μM
180 μM
12 μM
71 μM
6.0 μM
7.8 μM
0.46 μM
1 μm
B
Pat1
MD
HEAT
Pat1:
Similar C sat as Pat1 241-422
Lower [condensed phase] than Pat1 241-422
Oligomerization domain

Figure S13. Titration of Pat1 truncations.

(A) Representative micrographs for titration of Pat1 and truncations. Total concentration of protein and contrast are labeled above and below the micrographs, respectively. All are EGFP fusion proteins.

(B) Cartoon schematic representing: Pat1 has a similar saturation concentration (C_{sat}) as Pat1$^{241-422}$, and Pat1$^{241-422}$ has a higher condensate protein concentration at comparable total protein concentrations. These data suggest that either the Pat1$^{1-240}$ and/or Pat1$^{423-796}$ interact with Pat1$^{241-422}$ to modulate LLPS in Pat1.

See also Fig. 4B and 4L.

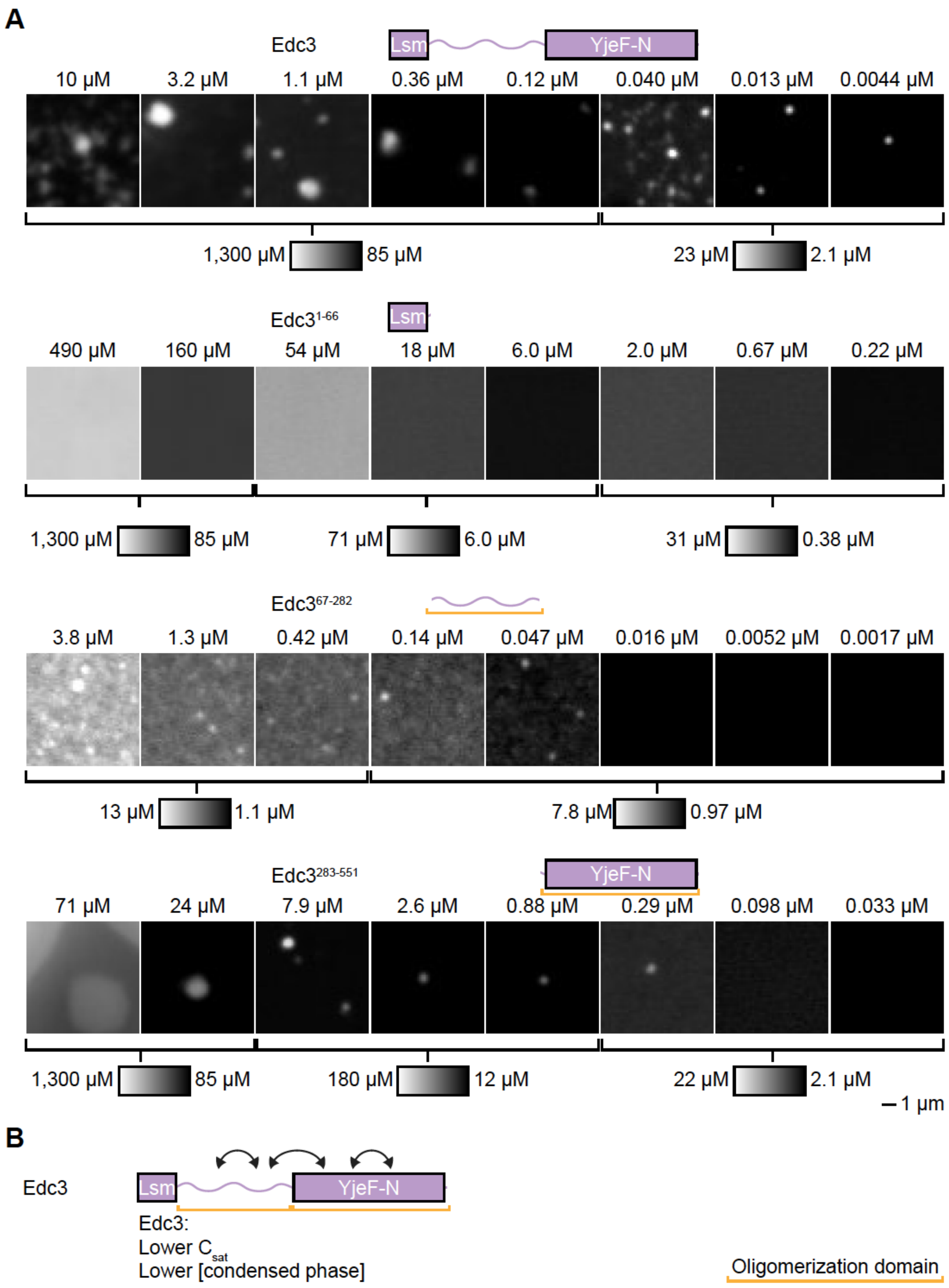
A
Edc3
Lsm
YjeF-N
10 µM
3.2 µM
1.1 µM
0.36 µM
0.12 µM
0.040 µM
0.013 µM
0.0044 µM
1,300 µM
85 µM
23 µM
2.1 µM
Edc3 1-66
Lsm
490 µM
160 µM
54 µM
18 µM
6.0 µM
2.0 µM
0.67 µM
0.22 µM
1,300 µM
85 µM
71 µM
6.0 µM
31 µM
0.38 µM
Edc3 67-282
3.8 µM
1.3 µM
0.42 µM
0.14 µM
0.047 µM
0.016 µM
0.0052 µM
0.0017 µM
13 µM
1.1 µM
7.8 µM
0.97 µM
Edc3 283-551
YjeF-N
71 µM
24 µM
7.9 µM
2.6 µM
0.88 µM
0.29 µM
0.098 µM
0.033 µM
1,300 µM
85 µM
180 µM
12 µM
22 µM
2.1 µM
1 µm
B
Edc3
Lsm
YjeF-N
Edc3:
Lower C sat
Lower [condensed phase]
Oligomerization domain

Figure S14. Titration of Edc3 truncations.

(A) Representative micrographs for titration of Edc3 and truncations. Total concentration of protein and contrast are labeled above and below the micrographs, respectively. All are EGFP fusion proteins.

(B) Cartoon schematic representing: Edc3 has a lower saturation concentration (C_{sat}) than any of the truncations, suggesting that the disordered middle region and the structured YjeF-N domain synergize to drive C_{sat} lower in full-length Edc3. The condensate protein concentration for $Edc3^{283-551}$ is higher, albeit at higher total protein concentrations.

See also Fig. 4C and 4M.

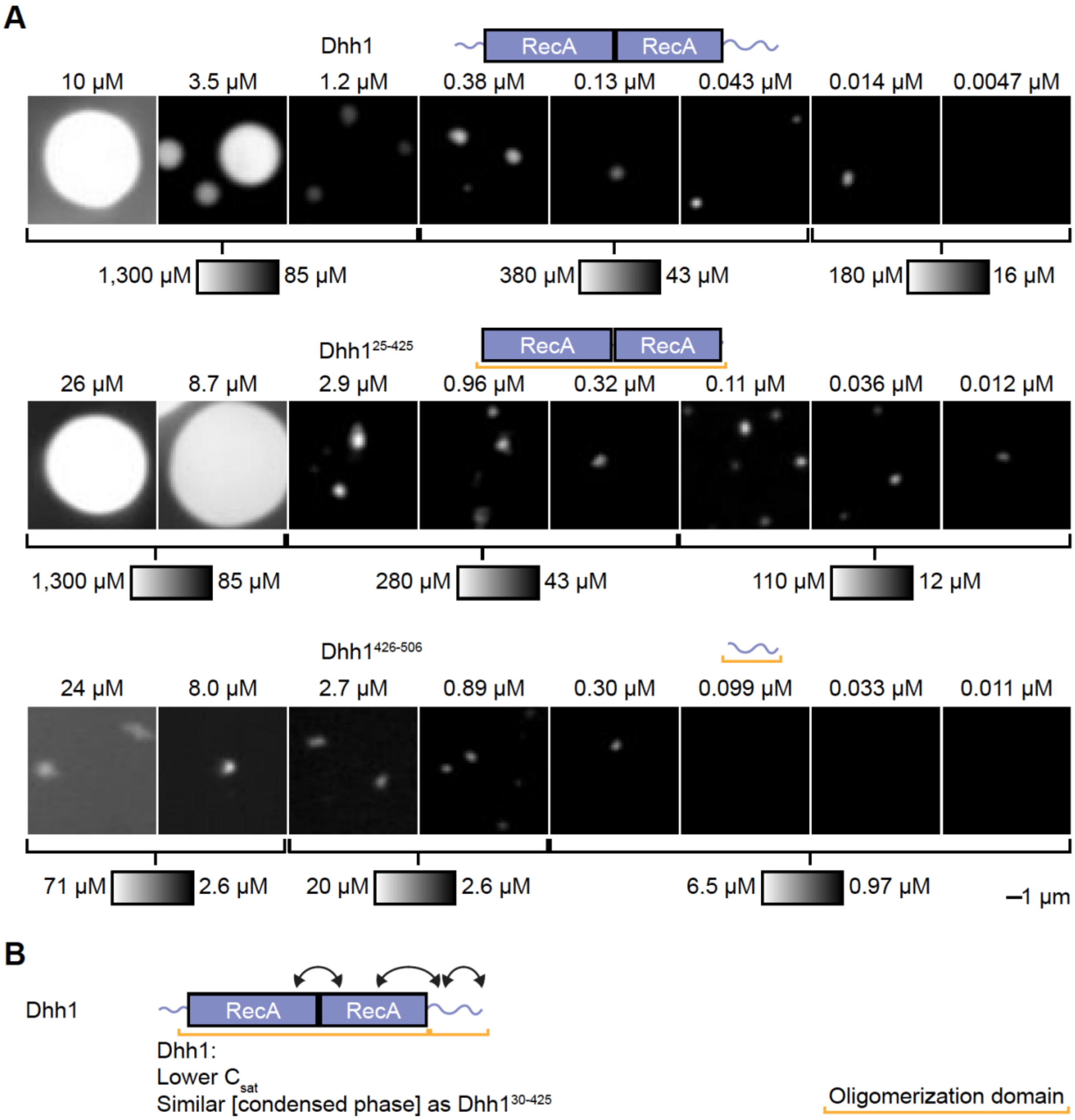

Figure S15. Titration of Dhh1 truncations.

(A) Representative micrographs for titration of Dhh1 and truncations. Total concentration of protein and contrast are labeled above and below the micrographs, respectively. All are EGFP fusion proteins.

(B) Cartoon schematic representing: Dhh1 has a lower saturation concentration (C_{sat}) than either of the truncations, suggesting that the structured tandem RecA domains and the disordered C-terminal region synergize to drive C_{sat} lower in full-length Dhh1. The condensate protein concentration for Dhh1$^{30-425}$ is similar to Dhh1 at comparable total protein concentrations.

See also Fig. 4D and 4N.

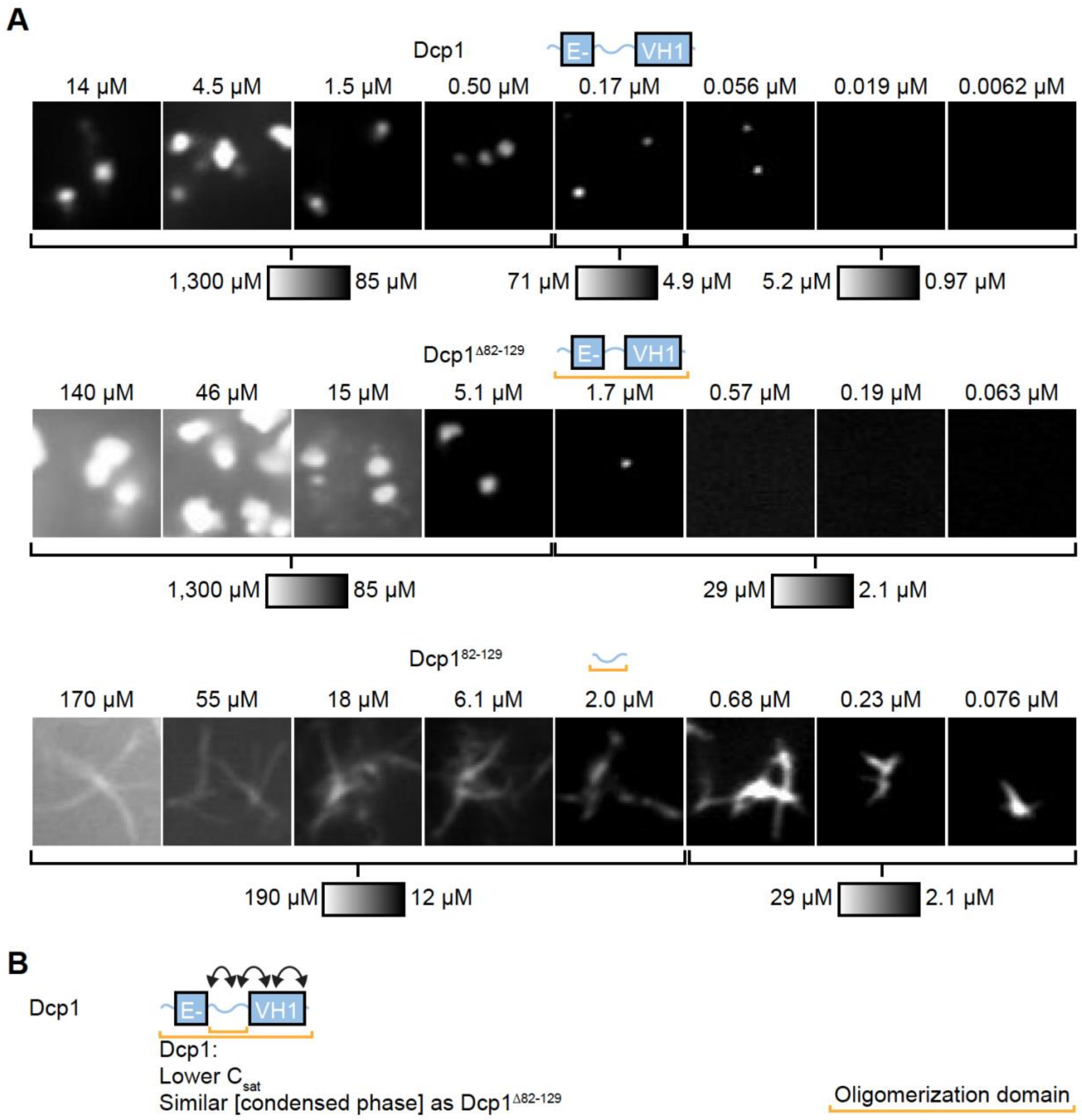

Figure S16. Titration of Dcp1 truncations.
(A) Representative micrographs for titration of Dcp1 and truncations. Total concentration of protein and contrast are labeled above and below the micrographs, respectively. All are EGFP fusion proteins.
(B) Cartoon schematic representing: Dcp1 has a lower saturation concentration (C_{sat}) than either of the truncations, suggesting that the structured EVH1 domain and the disordered middle linker synergize to drive C_{sat} lower in full-length Dcp1. The condensate protein concentration for Dcp1$^{\Delta 82\text{-}129}$ is similar to Dcp1, albeit at slightly higher total protein concentrations.

See also Fig. 4E and 4O.

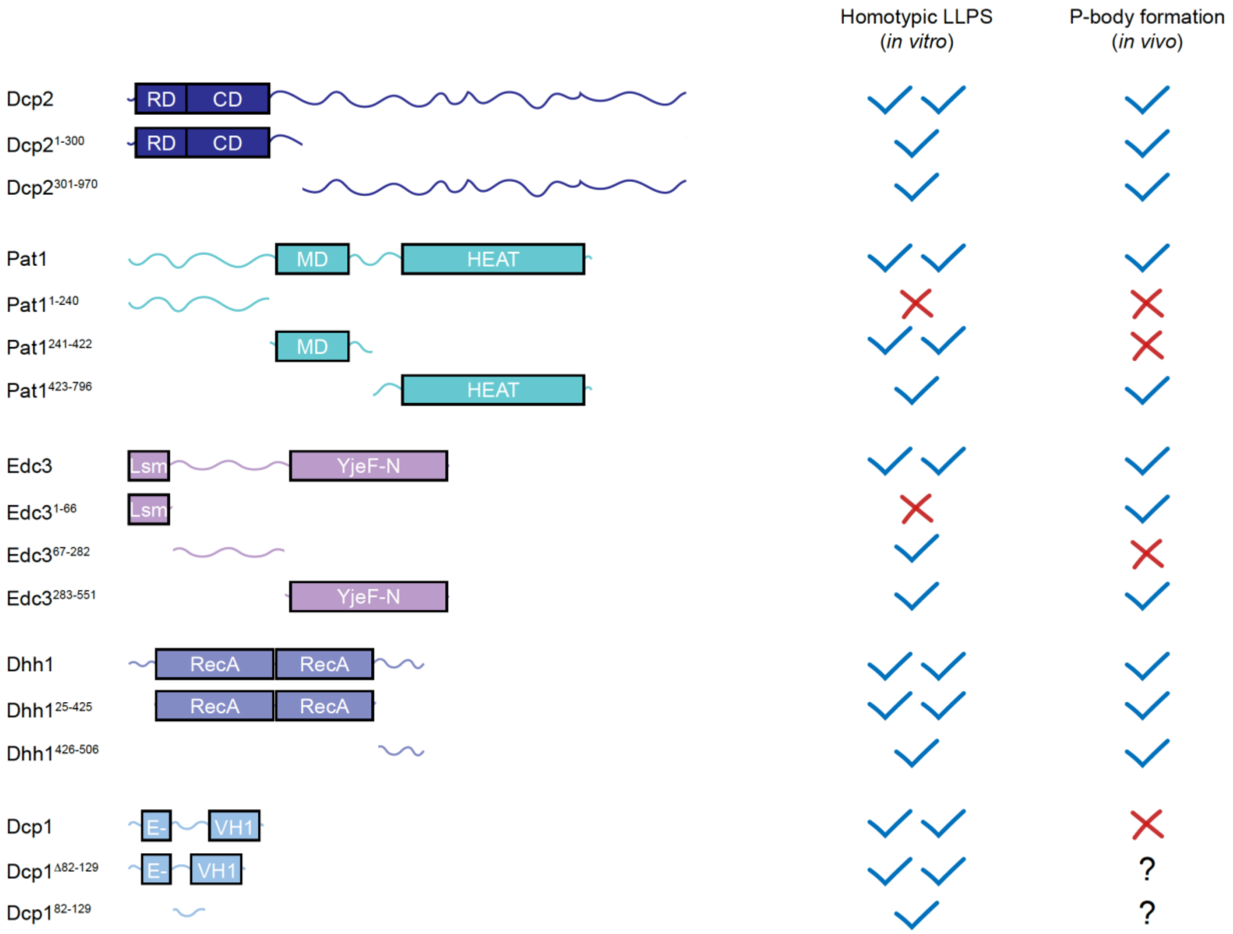

Figure S17. Comparison between P-body protein regions forming homotypic condensates *in vitro* and contributing to P-body formation *in vivo*.
The ability of P-body protein regions to exhibit homotypic LLPS *in vitro* (Fig. 4 and S12-16) and contribute to P-body formation *in vivo* (1, 22, 23, 30, 38) are scored in a qualitative fashion. Scoring for homotypic LLPS refers to experimental conditions and data from Fig. 4.

There is reasonable agreement between P-body protein regions that exhibit LLPS *in vitro* (Fig. 4), and contribute to P-body formation *in vivo* (Fig. S17). Our *in vitro* results are consistent with a synergy between multiple regions within Dcp2, and within Dhh1, contributing to P-body formation in cells (1, 38). Furthermore, our results are consistent with the importance of Edc3$^{283-551}$ and Pat1$^{423-796}$ contributing to P-body formation (23, 30). However, there are discrepancies for some P-body protein regions (Fig. S17). Edc3$^{1-66}$ does not form homotypic condensates, yet is important for P-body formation *in vivo* (23). This discrepancy is likely due to heterotypic interactions between Edc3$^{1-66}$ and helical leucine motifs in Dcp2 that contribute to P-body formation (1, 27). Dcp1 forms homotypic condensates *in vitro*, but represses P-body formation in cells by activating the RNA-decapping of Dcp2 (22), an activity not present in homotypic Dcp1 condensates. Edc3$^{67-282}$ and Pat1$^{241-422}$ form homotypic condensates *in vitro*, but are not important for P-body formation *in vivo* (23, 30). We do not currently understand these latter discrepancies, however there are some inherent limitations to our *in vitro* - *in vivo* comparisons

for P-body protein regions (Fig. S17). Since the threshold concentration for Edc3$^{67\text{-}282}$ is at least tenfold higher than Edc3 *in vitro* (Fig. S14), one possibility is that Edc3$^{67\text{-}282}$ may contribute to P body formation, but only at higher expression levels than were tested *in vivo* (23). Furthermore, our *in vitro* assays are quantitative whereas some of the previous cellular data is more qualitative in scoring for P-body formation based on one or two molecular markers (23, 30). Thus, protein regions with weaker contributions to phase separation, such as Edc3$^{67\text{-}282}$, may be missed by qualitative examinations in cells. While investigating the LLPS of isolated protein regions informs on the importance of homotypic interactions, this approach does not always correlate with condensate formation in cells. For example, regions whose contributions to P-body formation are mediated via heterotypic interactions with other components will be missed by this approach (see Fig. 5 and Discussion).

See also Fig. 4, S12-16, and Discussion.

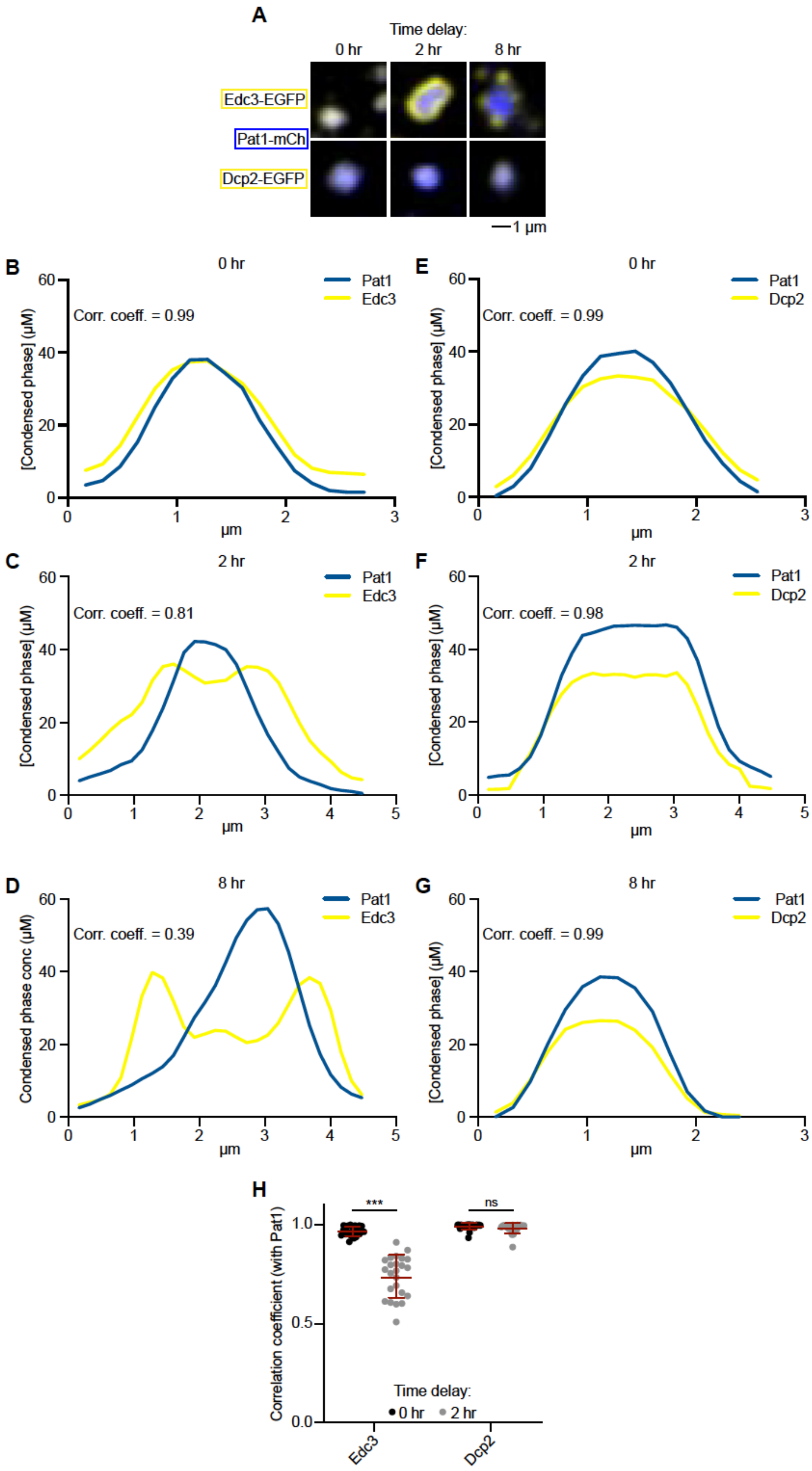
A
Time delay:
0 hr
2 hr
8 hr
Edc3-EGFP
Pat1-mCh
Dcp2-EGFP
1 µm
B
0 hr
Pat1
Edc3
Corr. coeff. = 0.99
[Condensed phase] (µM)
µm
C
2 hr
Pat1
Edc3
Corr. coeff. = 0.81
[Condensed phase] (µM)
µm
D
8 hr
Pat1
Edc3
Corr. coeff. = 0.39
Condensed phase conc (µM)
µm
E
0 hr
Pat1
Dcp2
Corr. coeff. = 0.99
[Condensed phase] (µM)
µm
F
2 hr
Pat1
Dcp2
Corr. coeff. = 0.98
[Condensed phase] (µM)
µm
G
8 hr
Pat1
Dcp2
Corr. coeff. = 0.99
[Condensed phase] (µM)
µm
H
***
ns
Correlation coefficient (with Pat1)
Time delay:
0 hr
2 hr
Edc3
Dcp2

Figure S18. Line profiles for overlap between Pat1 and Dcp2 or Edc3.
(A) Representative micrographs for overlap between Edc3-EGFP or Dcp2-EGFP with Pat1-mCh when Edc3 or Dcp2 are added at the time of condensate initiation (0 hr), and two or eight hours after condensate formation is initiated.
(B) Reactions were set up with all P-body proteins and RNA except Edc3. Edc3 was added back in as condensate formation was initiated (0 hr). Line profiles for Pat1 (blue) and Edc3 (yellow). Correlation coefficient between the two line profiles is displayed.
(C) As in (A) but Edc3 added 2 hours after condensate formation was initiated.
(D) As in (A) but Edc3 added 8 hours after condensate formation was initiated.
(E) As in (B) except Dcp2 was set aside instead of Edc3. Dcp2 was added back as condensate formation was initiated (0 hr).
(F) As in (D) but Dcp2 was added 2 hours after condensate formation was initiated.
(G) As in (D) but Dcp2 was added 8 hours after condensate formation was initiated.
(H) Quantification of the Pearson correlation coefficients of Edc3 and Dcp2 with Pat1 when added at condensate initiation (black) or after condensates have been formed for 2 hours (gray). Thirty condensates were measured from two replicate experiments.

During the course of our experiments, we observed that sufficient preincubation (1.5 hours) of all molecules together was required before initiating the reactions with TEV cleavage in order for different proteins to colocalize with one another. This suggests that a balance between heterotypic and homotypic interactions leads to P-body formation (and also that the *in vitro* system is slow to equilibrate). To further investigate this idea we intentionally investigated time as a variable in this set of experiments. We incubated different subsets of the P-body reconstitution separately for different amounts of time after TEV cleavage was initiated, before mixing the solutions together. We found that Dcp2 is readily recruited into the condensates containing the rest of the P-body proteins and RNA, at each timepoint tested. In contrast, Edc3 forms condensates that either coat (2 hr) or dock on the condensates with the rest of the P-body proteins and RNA. Images were taken 24 hr after TEV cleavage was initiated – and therefore 16-24 hr after the solutions were mixed together. Thus, these images likely represent the systems at equilibrium. One interpretation of these data is that homotypic condensates mature over time and fail to coalesce with heterotypic condensates. Indeed, alternative lines of investigation (Fig. 5 and S28-S31) also suggest that homotypic condensates mature more rapidly than heterotypic condensates. However, we note that additional plausible interpretations for this data exist – such as potential differences in surface tension between homotypic and heterotypic condensates (39).

A

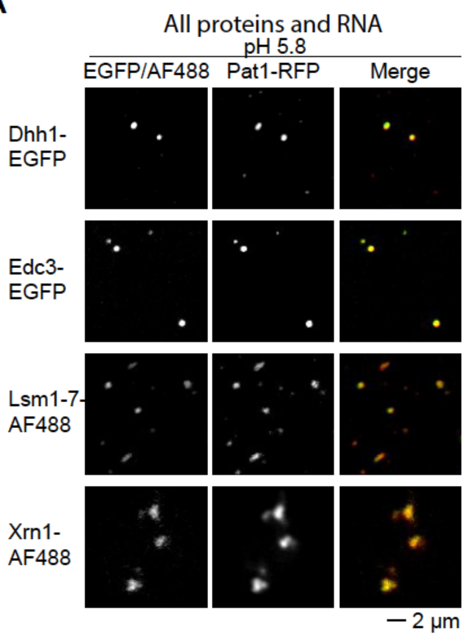
All proteins and RNA
pH 5.8
EGFP/AF488
Pat1-RFP
Merge
Dhh1-EGFP
Edc3-EGFP
Lsm1-7-AF488
Xrn1-AF488
2 µm

B

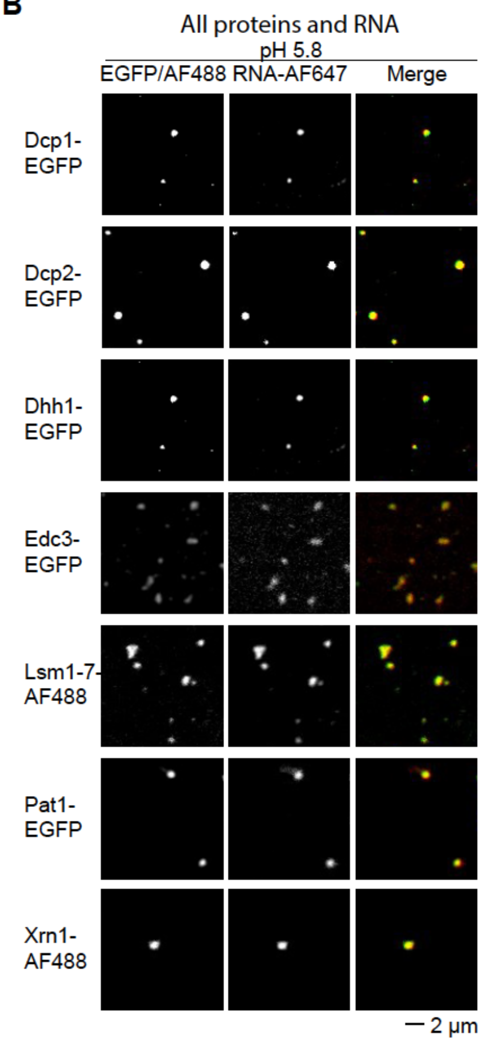
All proteins and RNA
pH 5.8
EGFP/AF488
RNA-AF647
Merge
Dcp1-EGFP
Dcp2-EGFP
Dhh1-EGFP
Edc3-EGFP
Lsm1-7-AF488
Pat1-EGFP
Xrn1-AF488
2 µm

Figure S19. All P-body proteins overlap with Pat1 and RNA in *in vitro* P-bodies under acidic (pH 5.8) conditions.

(A) Representative micrographs for Dhh1, Edc3, and Lsm1-7 overlapping with Pat1 under acidic conditions (pH 5.8).

(B) Representative micrographs for Dcp1, Dcp2, Dhh1, Edc3, Lsm1-7, and Pat1 overlap with RPL41A RNA under acidic conditions (pH 5.8).

Dcp1, Dcp2, Dhh1, and Edc3 are EGFP-fusion proteins and Lsm1-7 and Xrn1 are conjugated to AlexaFluor488.

See also Fig. 5A-B and S22.

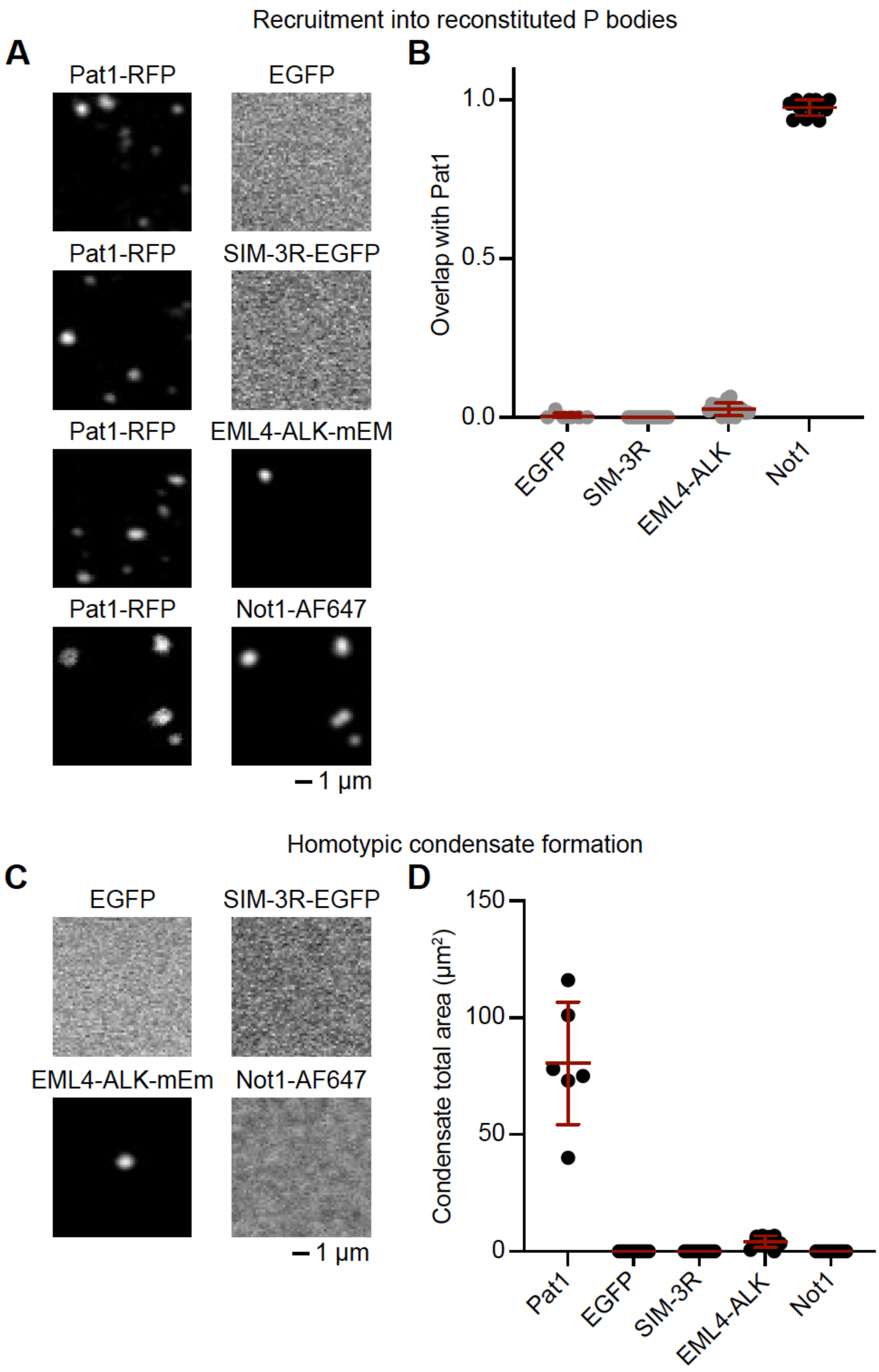

Recruitment into reconstituted P bodies
A
Pat1-RFP
EGFP
Pat1-RFP
SIM-3R-EGFP
Pat1-RFP
EML4-ALK-mEM
Pat1-RFP
Not1-AF647
1 μm
B
Overlap with Pat1
1.0
0.5
0.0
EGFP
SIM-3R
EML4-ALK
Not1
Homotypic condensate formation
C
EGFP
SIM-3R-EGFP
EML4-ALK-mEm
Not1-AF647
1 μm
D
Condensate total area (μm²)
150
100
50
0
Pat1
EGFP
SIM-3R
EML4-ALK
Not1

Figure S20. Specific interactions drive partitioning into reconstituted P-bodies.
(A) Representative micrograph of reconstituted P-bodies using Pat1-mCh as a marker, and with free EGFP (not tagged to any P-body protein), SIM-3R-EGFP, EML4-ALK-mEmerald, and Not1$^{754-1000}$-Alexa Flour 647 added. All P-body proteins and RNA were included in the reaction mixture, and P-bodies were formed at pH 5.8 and 300 mM KOAc. Micrographs in the same row represent different channels of the same experiment.
(B) Quantification of overlap in condensates between the Pat1-mCh and other channels.
(C) Representative micrographs of homotypic condensates formed by EGFP, SIM-3R, EML4-ALK, and Not1$^{754-1000}$. Experimental conditions as described in (A), except only the individual proteins were included in these reactions (no P body proteins and RNA).
(D) Total condensate area per slide for control proteins. Pat1 data included for reference.
EGFP and SIM-3R do not partition into reconstituted P bodies, nor do they form homotypic condensates on their own. EML4-ALK forms a few small homotypic condensates, and these condensates rarely overlap with reconstituted P bodies. Not1, a protein recruited to P bodies in cells, does not form homotypic condensates and is recruited into reconstituted P bodies, likely through interaction with Dhh1 (21). Collectively, these results suggest that *in vitro* P bodies are formed by specific interactions involving P-body protein and RNA molecules.

See also Fig. 5.

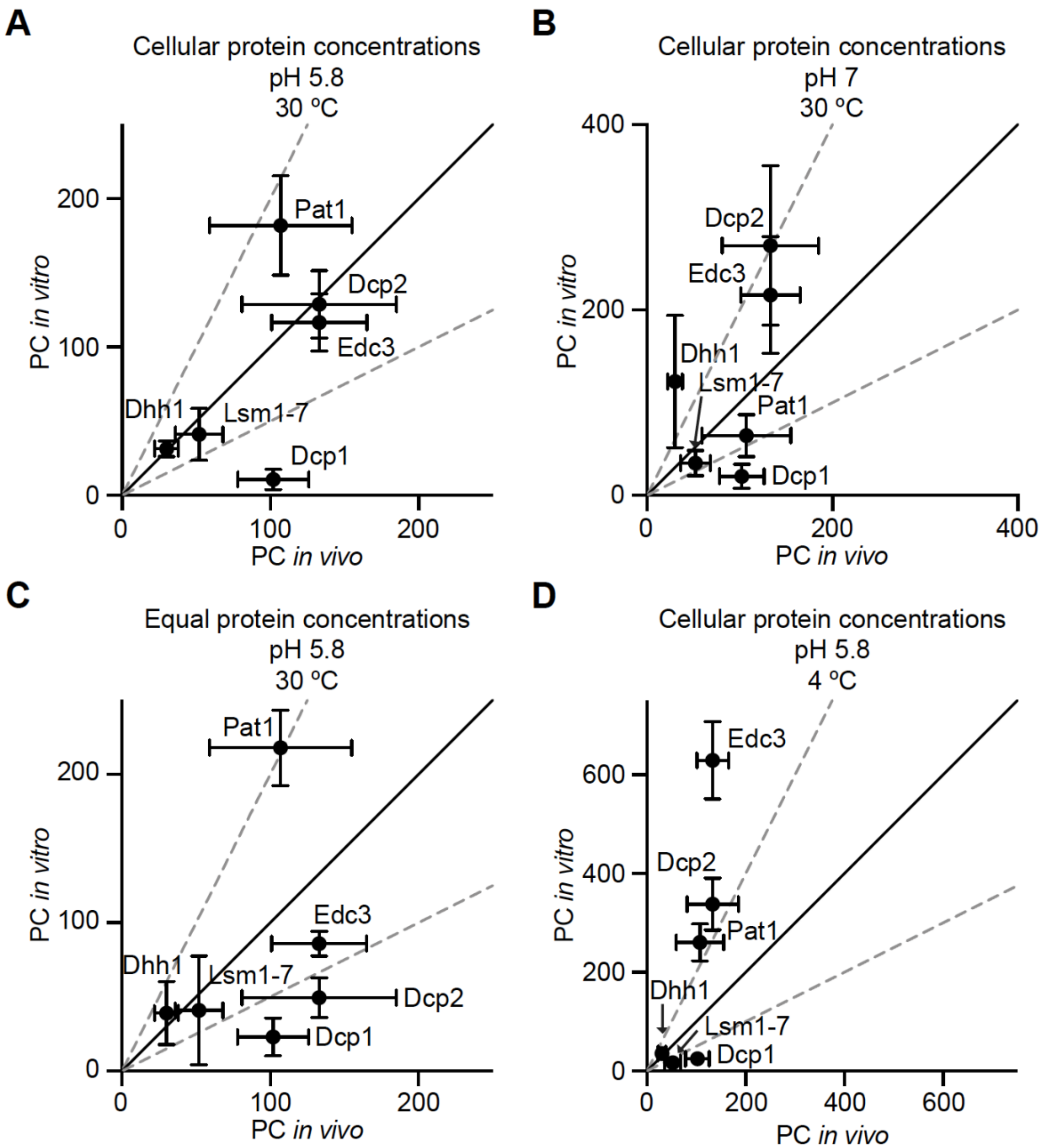

Figure S21. Correlation between P-body protein partition coefficients *in vitro* and *in vivo*.
We compared the partition coefficients of P-body proteins in *S. cerevisiae (1)*, to *in vitro* values observed under different experimental conditions.
(A) Cellular protein concentrations (range 90 – 370 nM, see Figure S5 and Methods), pH 5.8, and 30 °C incubation temperature. Black diagonal line is where *in vivo* and *in vitro* values are equivalent, gray dotted lines indicate twofold differences. This is the same data as in Fig. 5C, included here for reference to other experimental conditions.

(B) Same as (A) but pH 7 instead of pH 5.8.
(C) Same as (A) but with 150 nM input concentrations for all proteins and RNA.
(D) Same as (A) but with 4 °C instead of 30 °C incubation.
Note the difference in axes scales in (B) and (D). Partition coefficients for all proteins are closest to *in vivo* values with cellular protein concentrations, pH 5.8, and 30 °C incubation, and provide validation for using these experimental conditions. Furthermore, these data suggest that the stoichiometry of P bodies are sensitive to parameters such as pH, protein concentrations, and temperature.

See also Fig. 5C and S5.

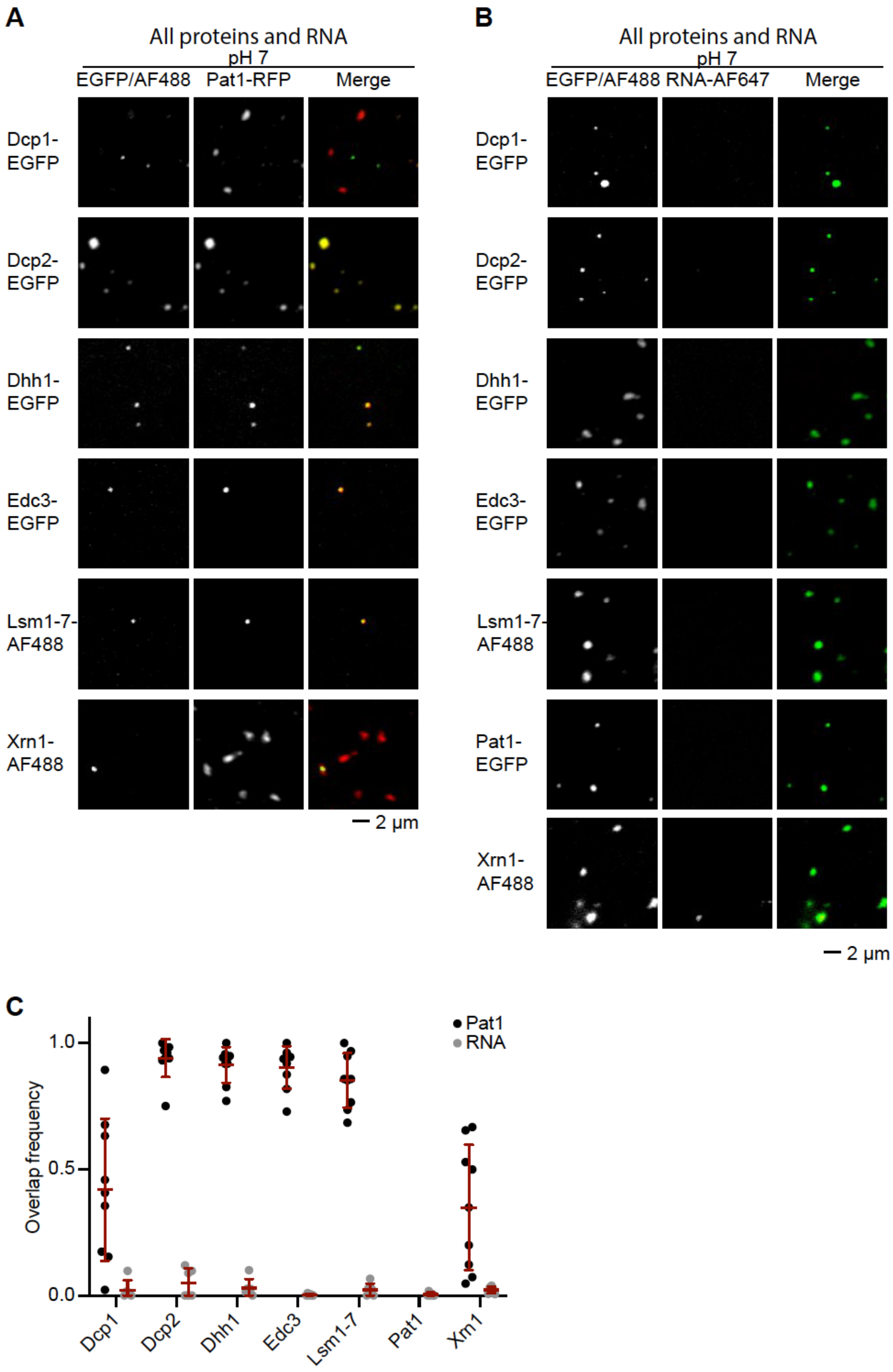
A
All proteins and RNA
pH 7
EGFP/AF488
Pat1-RFP
Merge
Dcp1-EGFP
Dcp2-EGFP
Dhh1-EGFP
Edc3-EGFP
Lsm1-7-AF488
Xrn1-AF488
2 µm
B
All proteins and RNA
pH 7
EGFP/AF488
RNA-AF647
Merge
Dcp1-EGFP
Dcp2-EGFP
Dhh1-EGFP
Edc3-EGFP
Lsm1-7-AF488
Pat1-EGFP
Xrn1-AF488
2 µm
C
Overlap frequency
1.0
0.5
0.0
Pat1
RNA
Dcp1
Dcp2
Dhh1
Edc3
Lsm1-7
Pat1
Xrn1

Figure S22. Less overlap between P-body components at neutral pH (7.0).
(A) Representative micrographs for Dcp1, Dcp2, Dhh1, Edc3, and Lsm1-7 overlapping with Pat1 under neutral conditions (pH 7.0).
(B) Representative micrographs for Dcp1, Dcp2, Dhh1, Edc3, Lsm1-7, and Pat1 overlap with RPL41A RNA under neutral conditions (pH 7.0).
(C) Overlap between Pat1 (black) or RNA (gray) and P-body proteins.
Dcp1, Dcp2, Dhh1, and Edc3 are EGFP-fusion proteins and Lsm1-7 and Xrn1 are conjugated to AlexaFluor488.
Relative to acidic pH (5.8) conditions (Fig. 5A-B and S19), there is less overlap between RNA with all P-body proteins, and between Dcp1 and Xrn1 with Pat1. These results suggest that acidic pH are the more relevant conditions for P-body formation as all components overlap in that condition.

See also Fig. 5A-B and S20.

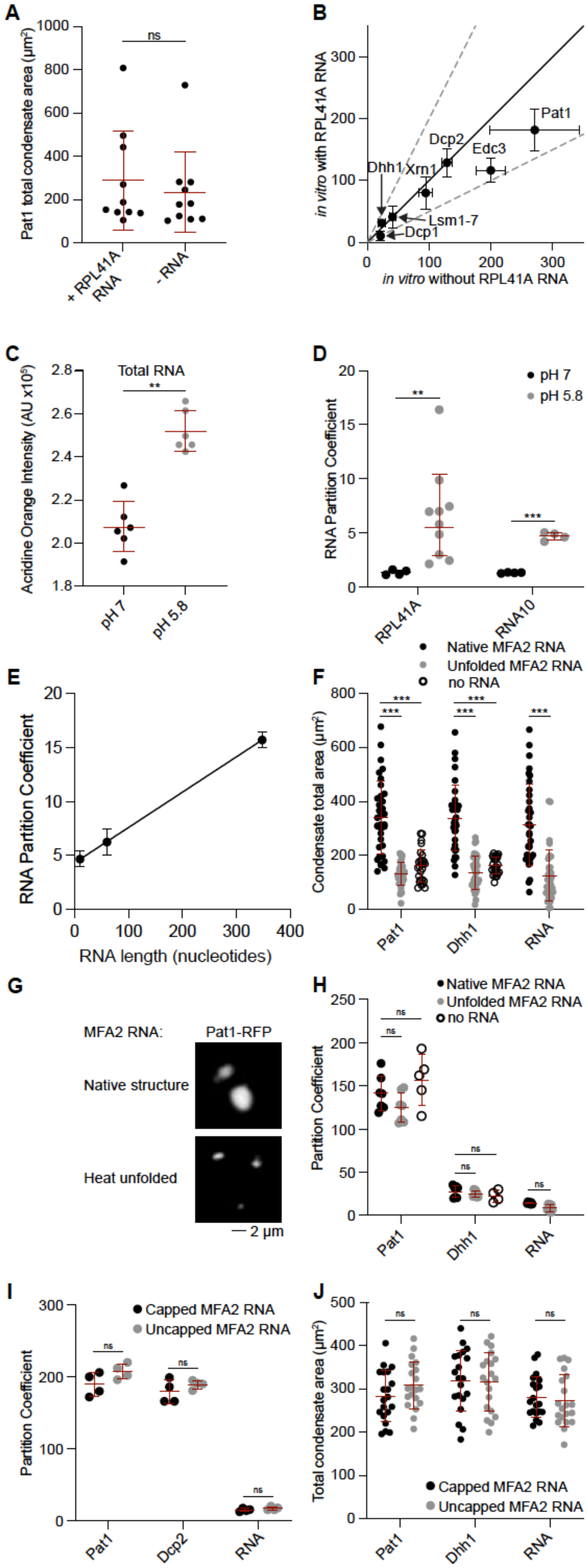
A
Pat1 total condensate area (µm²)
1000
800
600
400
200
0
ns
+ RPL41A RNA
- RNA
B
in vitro with RPL41A RNA
in vitro without RPL41A RNA
300
200
100
0
Pat1
Dcp2
Edc3
Dhh1
Xrn1
Lsm1-7
Dcp1
C
Acridine Orange Intensity (AU x10⁵)
Total RNA
**
2.8
2.6
2.4
2.2
2.0
1.8
pH 7
pH 5.8
D
RNA Partition Coefficient
pH 7
pH 5.8
**
***
20
15
10
5
0
RPL41A
RNA10
E
RNA Partition Coefficient
RNA length (nucleotides)
0
100
200
300
400
F
Condensate total area (µm²)
Native MFA2 RNA
Unfolded MFA2 RNA
no RNA
***
800
600
400
200
0
Pat1
Dhh1
RNA
G
MFA2 RNA:
Pat1-RFP
Native structure
Heat unfolded
— 2 µm
H
Partition Coefficient
Native MFA2 RNA
Unfolded MFA2 RNA
no RNA
ns
250
200
150
100
50
0
Pat1
Dhh1
RNA
I
Partition Coefficient
Capped MFA2 RNA
Uncapped MFA2 RNA
ns
300
200
100
0
Pat1
Dcp2
RNA
J
Total condensate area (µm²)
Capped MFA2 RNA
Uncapped MFA2 RNA
ns
500
400
300
200
100
0
Pat1
Dhh1
RNA

Figure S23. RNA does not strongly impact protein partitioning into *in vitro* P bodies.
(A) Condensate area, using Pat1 as a marker, is similar when all proteins are present with and without RPL41A RNA. Condensate area is similar with and without RNA regardless of which molecule is used as a marker.
(B) Protein partitioning is similar with and without MFA2 RNA.
(C) Acridine Orange intensity in P bodies when using total yeast RNA as the source of RNA.
(D) Partition coefficients for RPL41A and RNA10 RNAs at pH 7 (black) and pH 5.8 (gray).
(E) Correlation between RNA length and condensed phase concentration for different RNA species. Longer RNAs are more highly enriched in *in vitro* P bodies.
(F) Condensate area, using Pat1-RFP, Dhh1-EGFP, or MFA2 RNA-Cy5 as a marker, with natively structured MFA2 RNA (black filled circles), heat unfolded MFA2 RNA (gray filled circles, or no RNA (black open circles). Heat unfolded MFA2 RNA is incubated at 70 C for 15 minutes whereas natively structured MFA2 RNA is not.
(G) Representative micrograph sections for in vitro P bodies with natively structured and heat unfolded MFA2 RNA. Pat1-RFP used as a marker. Condensates look similar using Dhh1-EGFP and MFA2 RNA-Cy5 as the marker.
(H) Partition coefficients for Pat1-RFP, Dhh1-EGFP and MFA2 RNA-Cy5 with natively structured MFA2 RNA (black filled circles), heat unfolded MFA2 RNA (gray filled circles, or no RNA (black open circles).
(I) Partition coefficients for Pat1-RFP, Dhh1-EGFP and MFA2 RNA-Cy5 with capped MFA2 RNA (black circles) and uncapped MFA2 RNA (gray circles). MFA2 RNA is natively structured in this experiment.
(J) Condensate area for Pat1-RFP, Dhh1-EGFP and MFA2 RNA-Cy5 with capped MFA2 RNA (black circles) and uncapped MFA2 RNA (gray circles). MFA2 RNA is natively structured in this experiment.

Four different types of RNA were tested in our reconstitution: RNA10, a 10 nucleotide single stranded RNA; RPL41A, a 60 nucleotide RNA consisting of portions of the coding and untranslated regions of ribosomal protein of the large subunit 41A; MFA2, a 348 nucleotide full-length mRNA of yeast Mating Factor Alpha that is known to localize to P bodies; and total RNA from yeast. For all RNA sources we found that RNA had little impact on condensate formation and partitioning of proteins into condensates. Furthermore, we observed that all RNAs were more highly enriched into condensates under acidic pH conditions, consistent with enhanced binding with P-body proteins under these conditions (Fig. 3). Lastly, the partitioning of RNA species correlates with their length. These data indicate that RNA is not required for in vitro P body formation and does not impact protein partitioning, but can increase condensate size. One caveat here is that longer and more structured mRNAs, which are more highly enriched in cellular P bodies (40-42), may have more dramatic influences on protein partitioning and condensate size.

See also Fig. S11, S19, S22, and Methods.

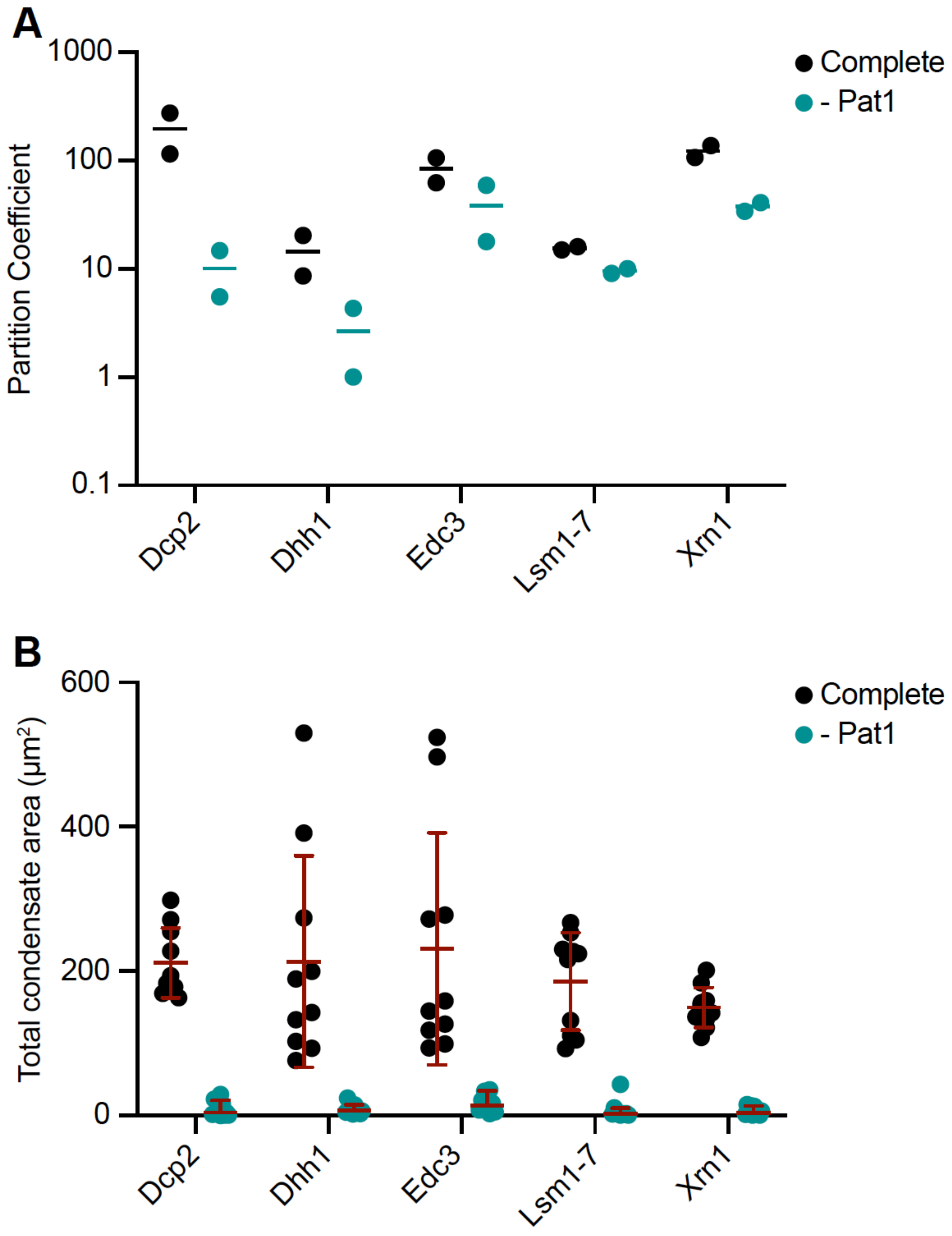

Figure S24. Loss of Pat1 reduces the partition coefficient of other P-body proteins.

(A) Partition coefficients with samples containing all proteins and RPL41A RNA (Complete, black circles) and with all proteins, except Pat1, and RPL41A RNA (- Pat1, teal circles).
(B) Total condensate area per micrograph with the same conditions as (A).
Pat1 contributes to P-body formation and increases the partitioning of the other molecules into P bodies.

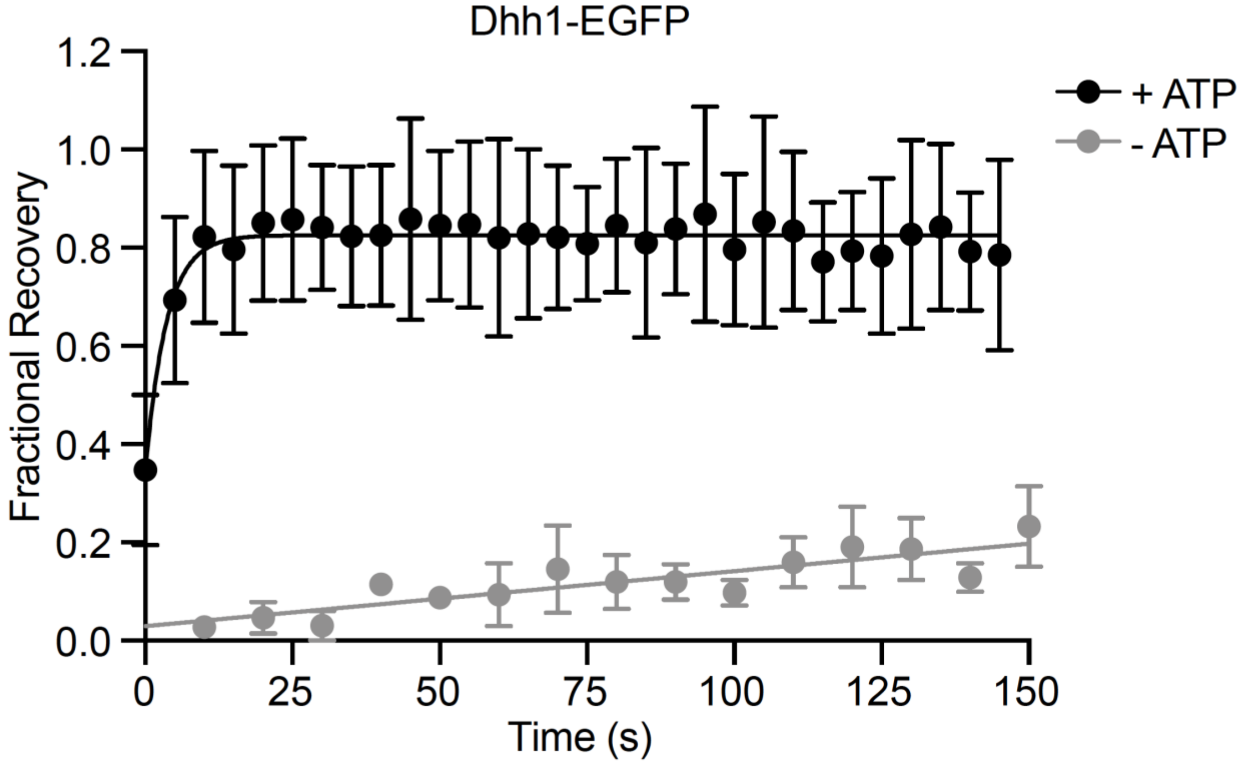

Figure S25. ATP is required for rapid dynamics of Dhh1 *in vitro*.
Recovery of Dhh1 in FRAP experiments with (black) or without (gray) ATP included in the reaction mix. The condensates for these experiments contained all proteins and RPL41A RNA. This finding suggests that ATP hydrolysis is necessary to mimic the cellular dynamics of Dhh1 *in vitro*, and is consistent with the finding that an ATP-binding deficient mutant of Dhh1 has reduced dynamics in cells (1, 21).

See also Fig. 5D.

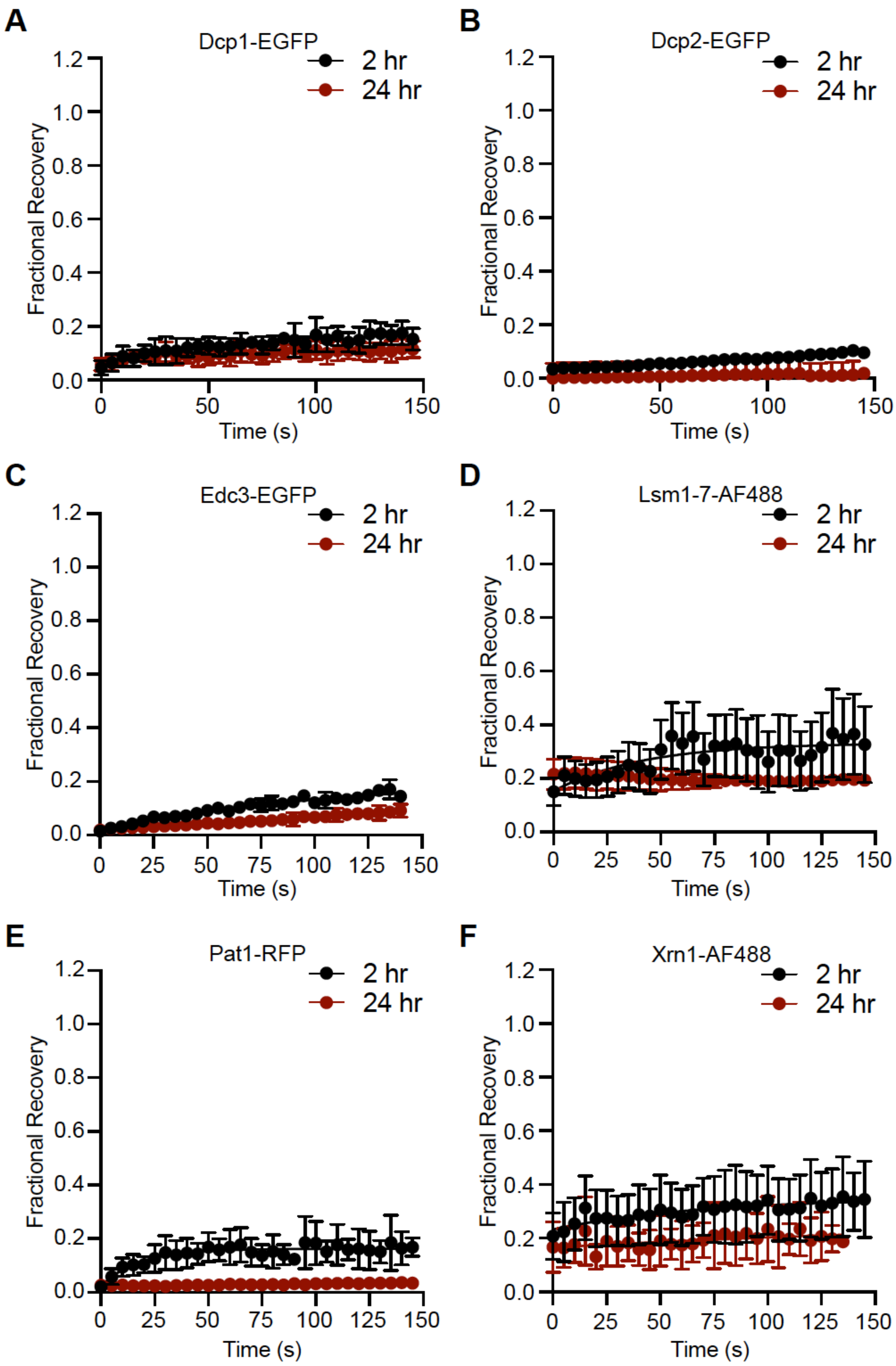
A
Dcp1-EGFP
B
Dcp2-EGFP
C
Edc3-EGFP
D
Lsm1-7-AF488
E
Pat1-RFP
F
Xrn1-AF488
2 hr
24 hr
Fractional Recovery
Time (s)

Figure S26. Proteins become less dynamic as P-bodies mature.

(A) Representative FRAP recovery curves for Dcp1 in heterotypic condensates with all P-body proteins and RPL41A RNA at 2 hr (black) and 24 hr (red).

(B) Dcp2 FRAP recovery curves as in (A).

(C) Edc3 FRAP recovery curves as in (A).

(D) Lsm1-7 FRAP recovery curves as in (A).

(E) Pat1 FRAP recovery curves as in (A).

(F) Xrn1 FRAP recovery curves as in (A).

See also Fig. 5E and 5F.

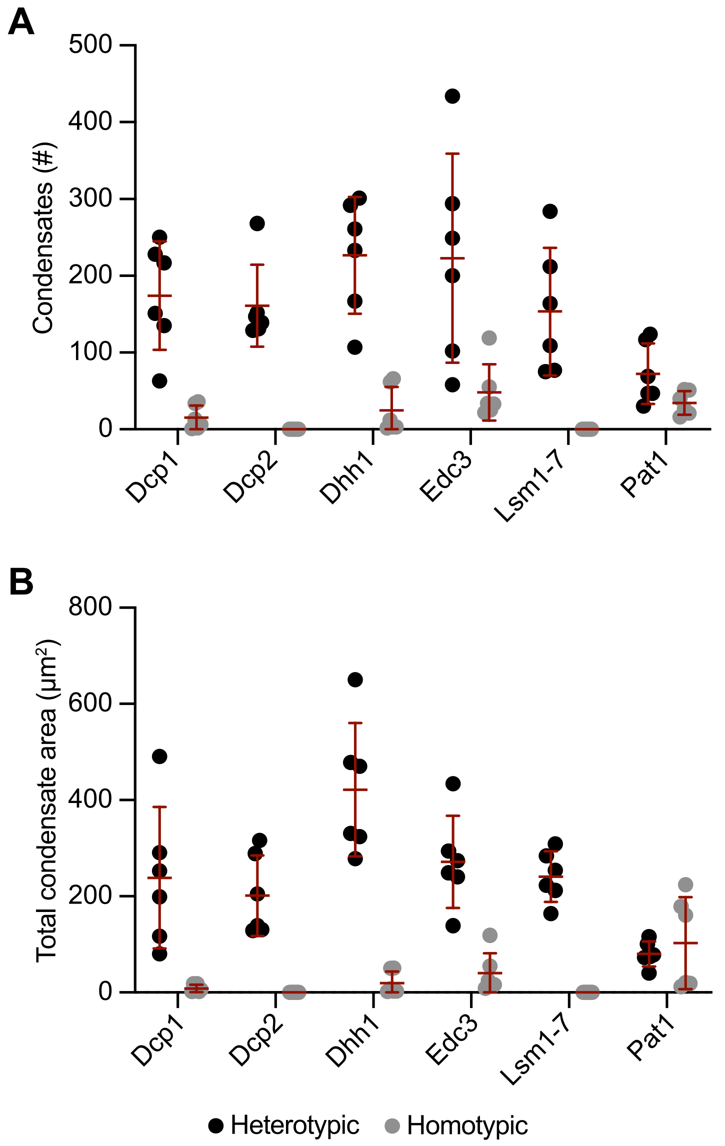

Figure S27. Comparison between homotypic and heterotypic condensates.
(A) Number of condensates were compared for P-body proteins either in the presence of all other P-body molecules (heterotypic, black) or as individual proteins (homotypic, gray).
(B) Total condensate area, as in (A).

The individual proteins Pat1, Edc3, and Dhh1 had the highest number of condensates and total condensate area, supporting their importance in P-body formation. The number and area of Pat1 alone is comparable to Pat1 in heterotypic condensates, suggesting the importance of Pat1 for P-body formation.

See also Fig. 5G.

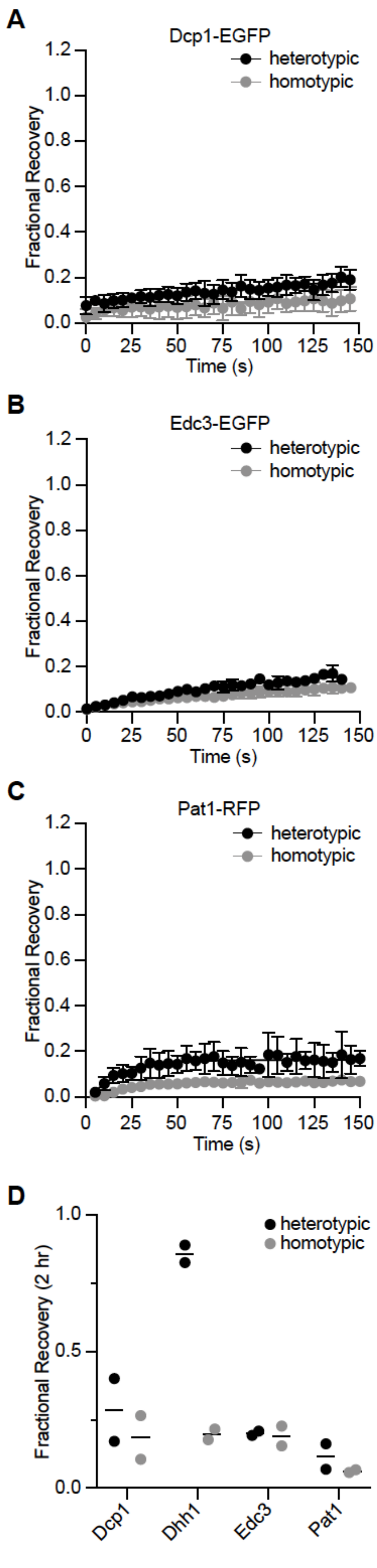

A
Dcp1-EGFP
heterotypic
homotypic
Fractional Recovery
Time (s)
B
Edc3-EGFP
heterotypic
homotypic
Fractional Recovery
Time (s)
C
Pat1-RFP
heterotypic
homotypic
Fractional Recovery
Time (s)
D
heterotypic
homotypic
Fractional Recovery (2 hr)
Dcp1
Dhh1
Edc3
Pat1

Figure S28. Dcp1, Edc3, and Pat1 are slightly more dynamic in heterotypic condensates.
(A) Representative FRAP recovery curves for Dcp1 in heterotypic (black) and homotypic (gray) condensates.
(B) Representative FRAP recovery curves for Edc3 as in (A).
(C) Representative FRAP recovery curves for Pat1 as in (A).
(D) Fractional recoveries of P-body proteins in heterotypic (all proteins and RPL41A RNA) and homotypic condensates.

All three proteins are slightly more dynamic in heterotypic condensates as compared to homotypic condensates.

See also Fig. 5H.

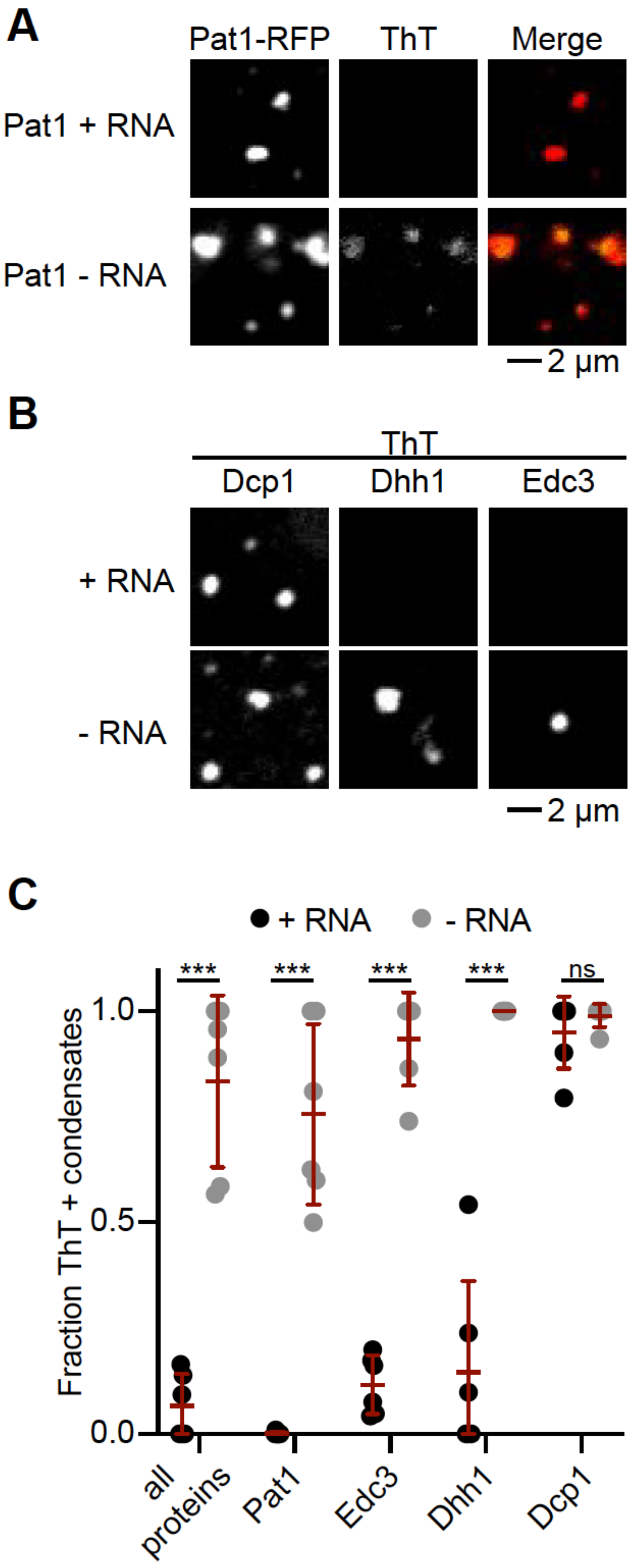
A
Pat1-RFP
ThT
Merge
Pat1 + RNA
Pat1 - RNA
2 μm
B
ThT
Dcp1
Dhh1
Edc3
+ RNA
- RNA
2 μm
C
+ RNA
- RNA
***
***
***
***
ns
Fraction ThT + condensates
1.0
0.5
0.0
all proteins
Pat1
Edc3
Dhh1
Dcp1

Figure S29. RNA prevents ThT staining of single-protein condensates.
(A) Representative micrographs monitoring Pat1-RFP (left), ThT staining (middle) and merge (left). Samples include RPL41A RNA in top row and are lack RNA in the bottom row.
(B) Representative micrographs of ThT staining for Dcp1 (left), Dhh1 (middle), and Edc3 (right). Fraction of ThT positive condensates for these proteins were estimated with comparison to GFP-tagged proteins measured in parallel.
(C) Fraction of ThT positive condensates for proteins with (black) and without (gray) RNA.

See also Fig. 5I.

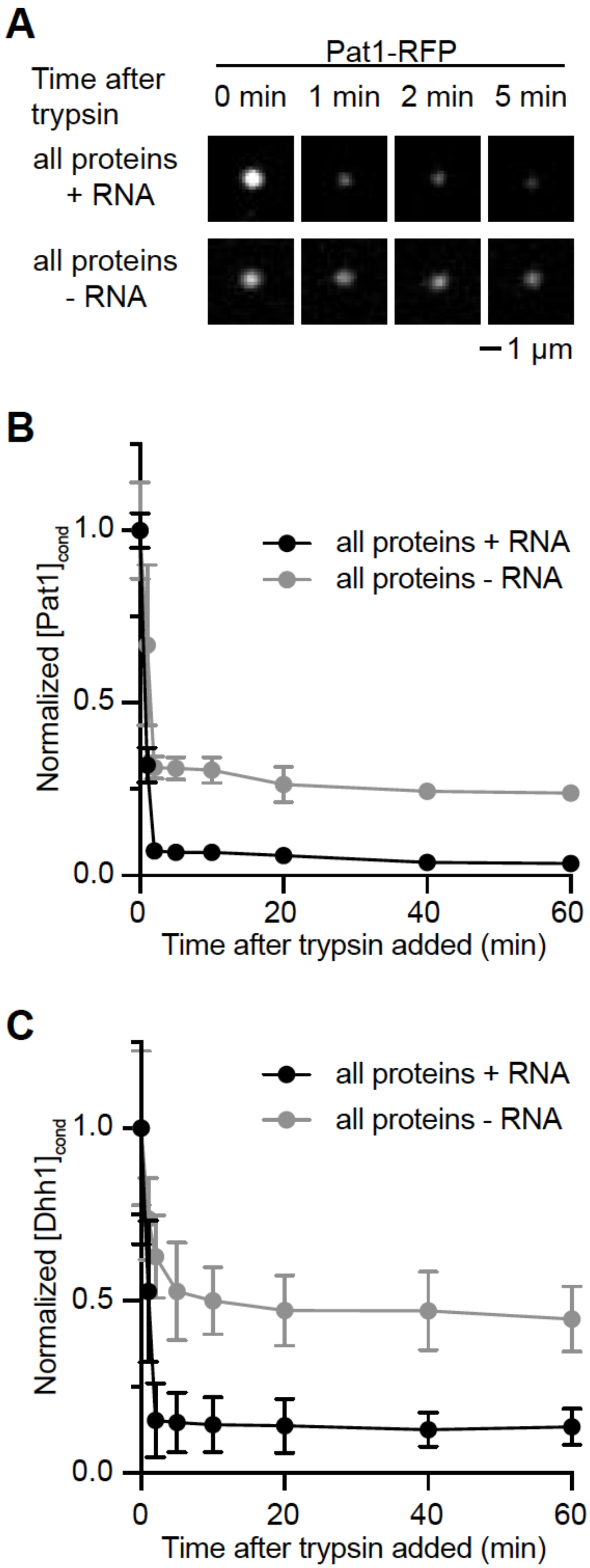
A
Time after trypsin
Pat1-RFP
0 min
1 min
2 min
5 min
all proteins + RNA
all proteins - RNA
1 μm
B
Normalized [Pat1]cond
1.0
0.5
0.0
0
20
40
60
Time after trypsin added (min)
all proteins + RNA
all proteins - RNA
C
Normalized [Dhh1]cond
1.0
0.5
0.0
0
20
40
60
Time after trypsin added (min)
all proteins + RNA
all proteins - RNA

Figure S30. RNA promotes proteolytic degradation of condensates.
(A) Micrographs of condensates, using Pat1-RFP as a marker, before and after adding trypsin. Top row are condensates with all proteins and RPL41A RNA, bottom row are condensates with all proteins but no RNA.
(B) Relative Pat1 condensate protein concentration before trypsin is added (0 min), and after trypsin is added. Black circles and line are for all proteins with RPL41A RNA; gray circles and line are for all proteins without RPL41A RNA.
(C) Relative Dhh1-EGFP condensate protein concentration before trypsin is added (0 min), and after trypsin is added.

Condensates with RPL41A RNA are more rapidly and completely proteolyzed by trypsin, suggesting that RNA promotes a more mobile and reversible condensate material state.

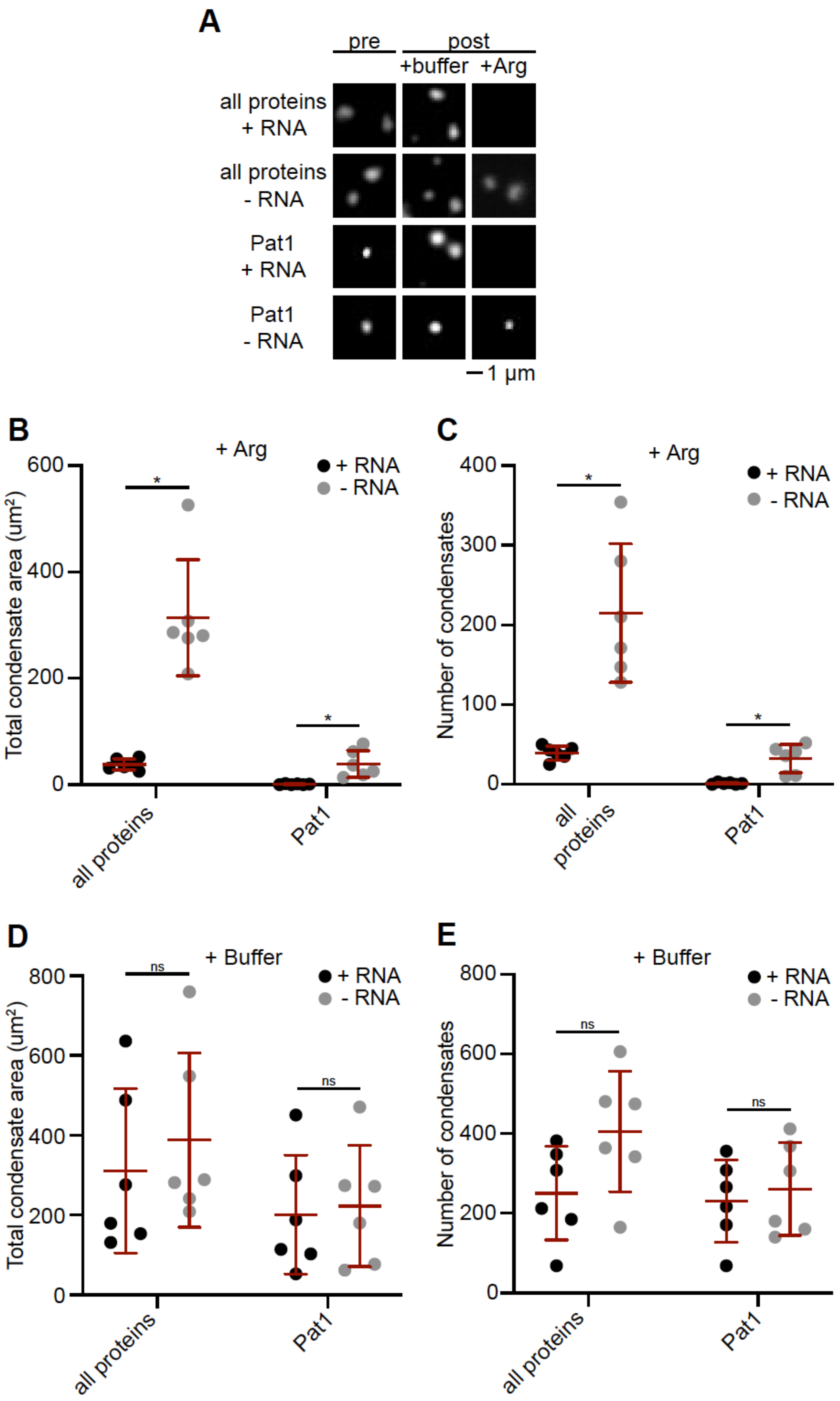
A
pre
post
+buffer
+Arg
all proteins
+ RNA
all proteins
- RNA
Pat1
+ RNA
Pat1
- RNA
1 µm
B
+ Arg
+ RNA
- RNA
Total condensate area (um²)
all proteins
Pat1
C
+ Arg
Number of condensates
all proteins
Pat1
D
+ Buffer
Total condensate area (um²)
ns
E
+ Buffer
Number of condensates
ns

Figure S31. Dissolution of RNA-containing condensates with arginine.
(A) Micrographs pre and post addition of either arginine chloride or equivalent volume of buffer. Molecules in condensates listed on the left. All condensates are being visualized with Pat1-mCh.
(B) Total condensate area per micrograph for condensates with (black) and without RPL41A RNA (gray) after addition of 500 mM arginine.
(C) Number of condensates per micrograph for condensates with (black) and without RPL41A RNA (gray) after addition of 500 mM arginine.
(D) Total condensate area per micrograph for condensates with (black) and without RPL41A RNA (gray) after addition of an equivalent volume of buffer to control for dilution as compared to the arginine treatment.
(E) Number of condensates per micrograph for condensates with (black) and without RPL41A RNA (gray) after addition of an equivalent volume of buffer to control for dilution as compared to the arginine treatment.

Arginine more readily dissolves condensates with RPL41A RNA compared to those without RNA. The buffer control demonstrates that condensates with all proteins or with Pat1 have similar number of condensates and similar condensate area with or without RNA.

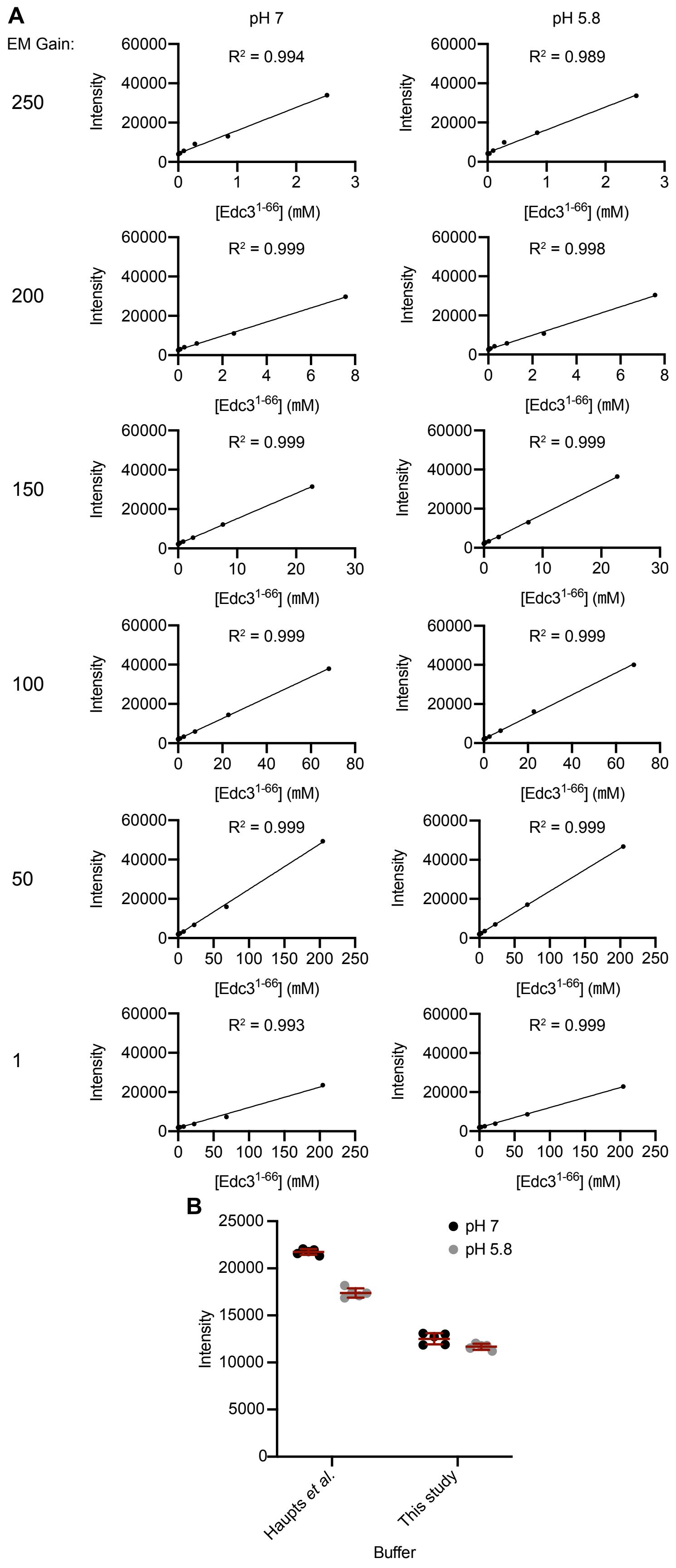
A
pH 7
pH 5.8
EM Gain:
250
200
150
100
50
1
R² = 0.994
R² = 0.989
R² = 0.999
R² = 0.998
R² = 0.999
R² = 0.999
R² = 0.999
R² = 0.999
R² = 0.999
R² = 0.999
R² = 0.993
R² = 0.999
Intensity
[Edc3^1-66] (mM)
B
pH 7
pH 5.8
Intensity
Haupts et al.
This study
Buffer

Figure S32. EGFP standard curves for quantitative microscopy.
(A) Standard curves were measured using six different EM gain settings to obtain a linear relationship between protein concentration and pixel intensity over a wide range of protein concentrations. Note the change in x-axis scale between different EM gain settings. Standard curves were done in pH 7 (left) and pH 5.8 (right). MBP-Edc3$^{1\text{-}66}$-EGFP was used to generate standard curves because it did not form condensates at any of these concentrations.
(B) In our buffer conditions we did not observe a significant difference in EGFP fluorescence at pH 7 and pH 5.8 [compare left and right columns in (A)], in contrast with a previous finding (43). Using previously reported buffer conditions ("Haupts *et al.*":10 mM citrate/citric acid buffer and 100 mM KOAc), we observe higher fluorescence intensity at pH 7, consistent with a previous report (43). However, under our buffer conditions fluorescence intensity is similar at pH 7 and pH 5.8. We hypothesize this difference is due to higher salt in our buffer (300 mM vs 125 mM KOAc), but did not further pursue identifying key differences between buffers.

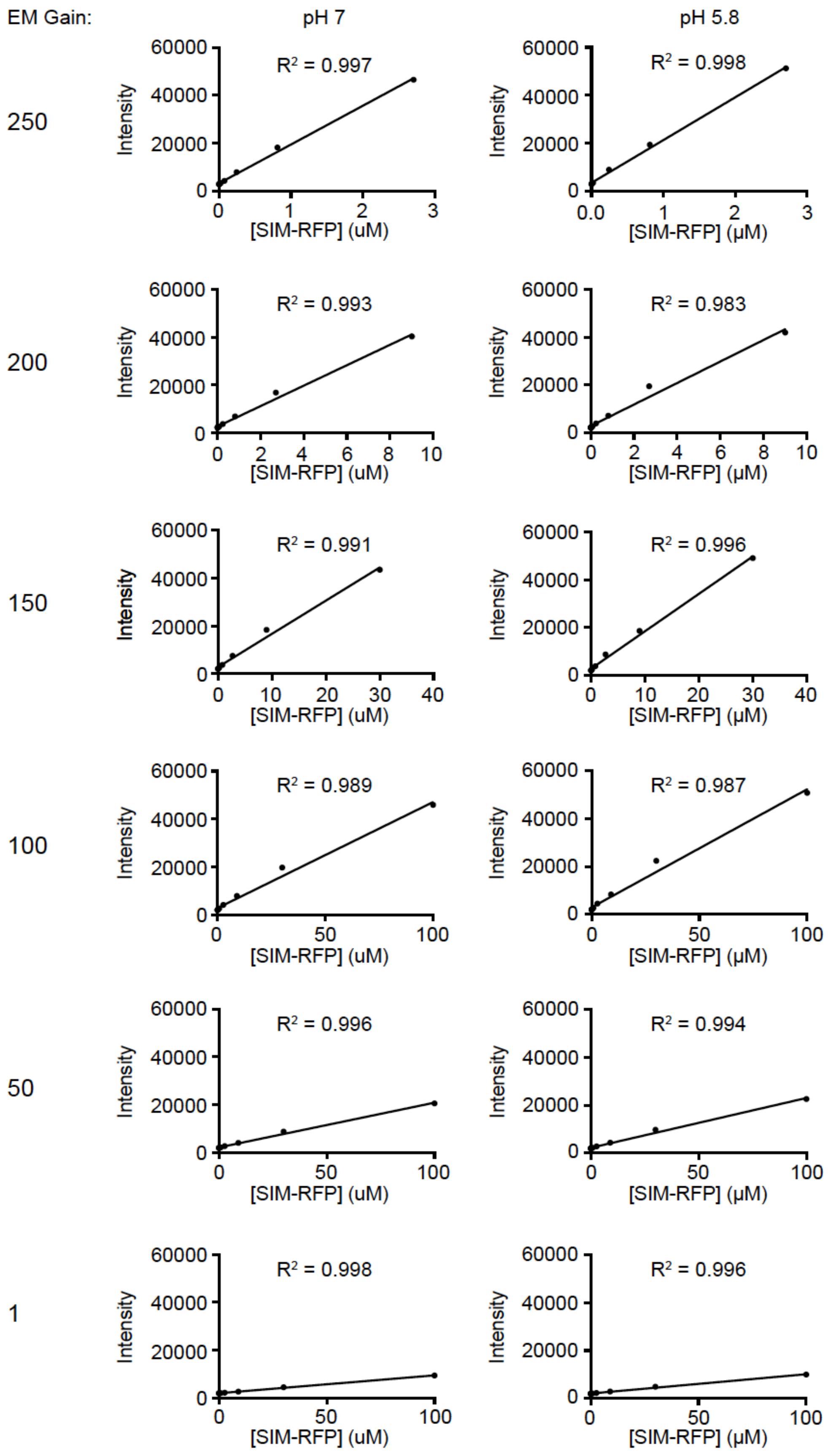
EM Gain:
pH 7
pH 5.8
250
R2 = 0.997
R2 = 0.998
200
R2 = 0.993
R2 = 0.983
150
R2 = 0.991
R2 = 0.996
100
R2 = 0.989
R2 = 0.987
50
R2 = 0.996
R2 = 0.994
1
R2 = 0.998
R2 = 0.996
Intensity
[SIM-RFP] (uM)
[SIM-RFP] (μM)

Figure S33. RFP standard curves for quantitative microscopy.
Standard curves were measured using six different EM gain settings to obtain a linear relationship between protein concentration and pixel intensity over a wide range of protein concentrations. Note the change in x-axis scale between different EM gain settings. Standard curves were done in pH 7 and pH 5.8. A single SUMO interacting motif (SIM) fused to RFP was used to generate standard curves because it did not form condensates at any of these concentrations.

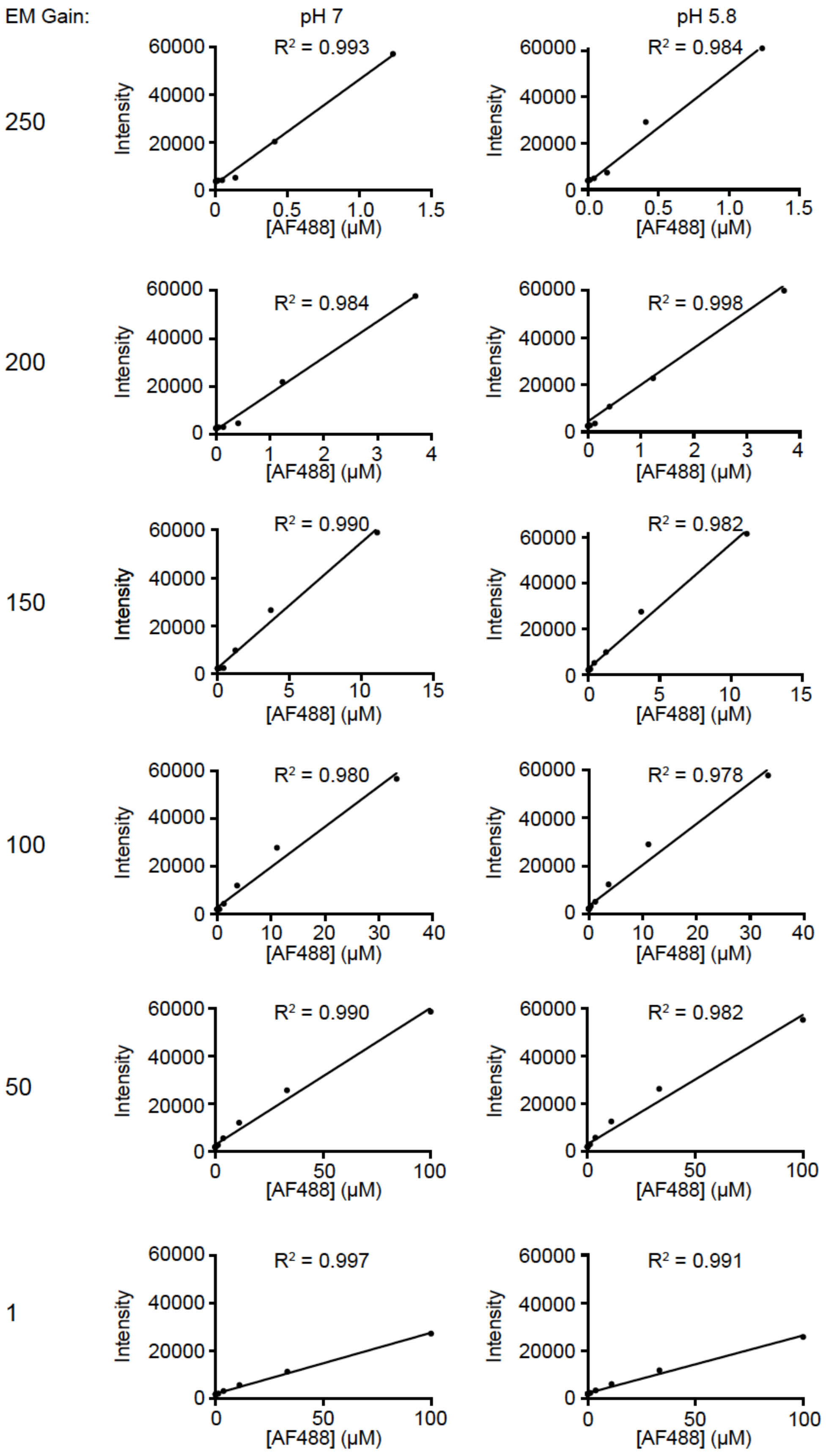
EM Gain:
pH 7
pH 5.8
250
R² = 0.993
R² = 0.984
200
R² = 0.984
R² = 0.998
150
R² = 0.990
R² = 0.982
100
R² = 0.980
R² = 0.978
50
R² = 0.990
R² = 0.982
1
R² = 0.997
R² = 0.991
Intensity
[AF488] (μM)

Figure S34. AlexaFluor488 (AF488) standard curves for quantitative microscopy.
Standard curves were measured using six different EM gain settings to obtain a linear relationship between protein concentration and pixel intensity over a wide range of protein concentrations. Note the change in x-axis scale between different EM gain settings. Standard curves were done in pH 7 and pH 5.8. Free AF488 (not bound to any protein) was used to generate standard curves.

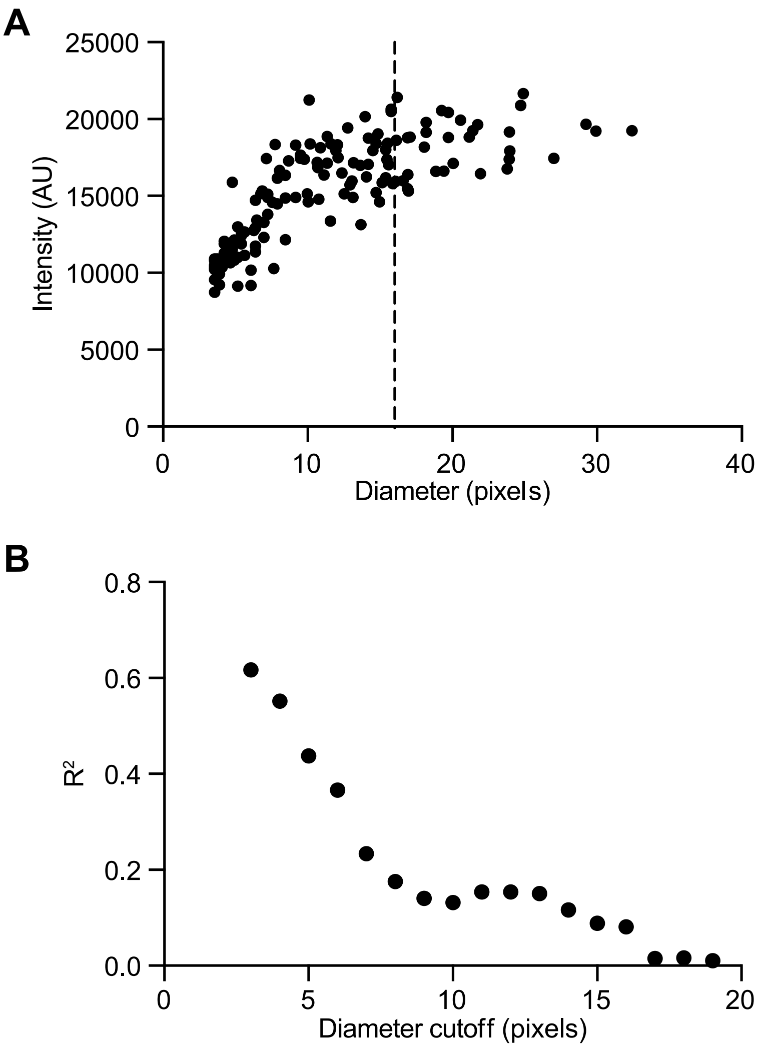

Figure S35. Diameter cutoff for measuring condensate concentration.
(A) Fluorescence intensity versus condensate diameter for Dhh1-GFP as a marker of *in vitro* P-bodies, i.e. with all other P body proteins and RNA.
(B) R^2 values measuring the linear relationship for the data in (A) above the given diameter cutoff on the x-axis.

For diameters greater than 16 pixels the fluorescence intensity is unrelated to the size of the condensate. 16 pixels corresponds to approximately 2.5 μm on our microscope/detector. Therefore condensates with diameters greater than 2.5 μm were selected for concentration measurements to avoid dilution of intensities in condensates close in size to the PSF.

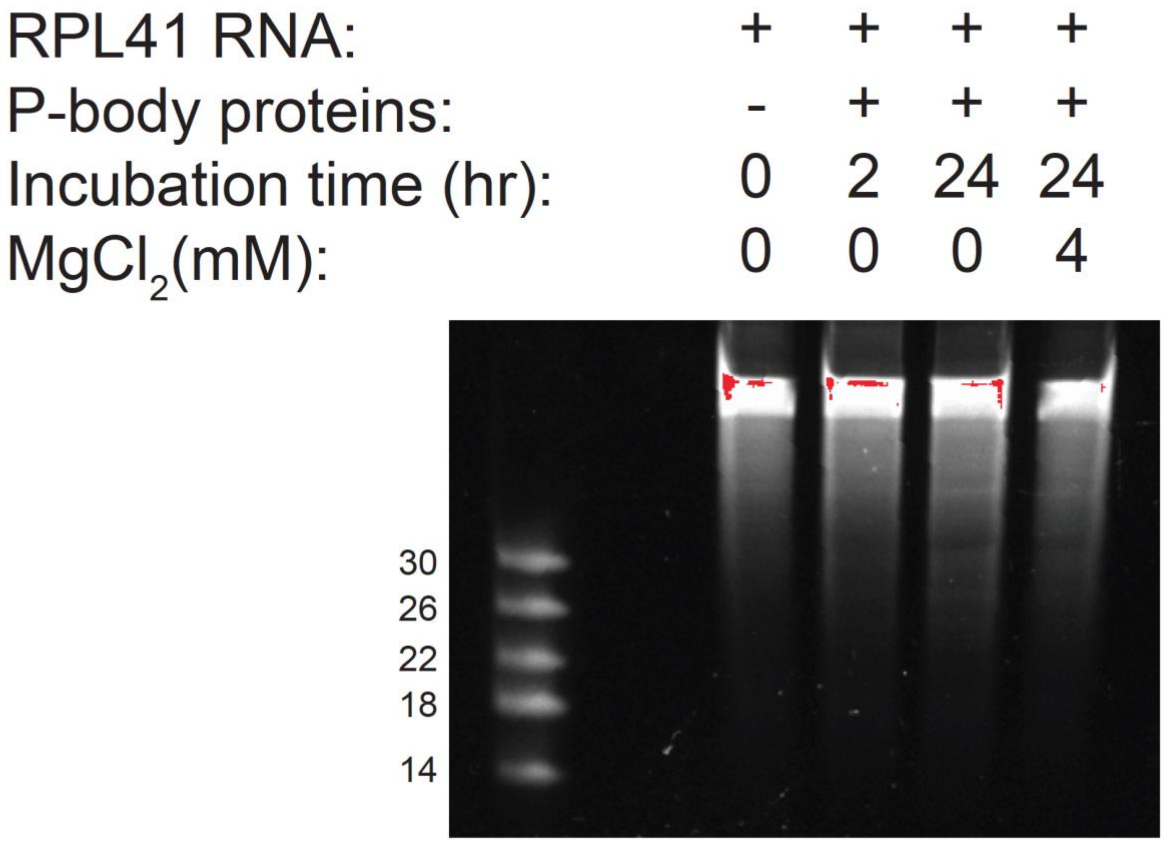

Figure S36. Xrn1 does not degrade RPL41A RNA under experimental conditions.
Urea-TBE gel of RPL41A RNA alone (first lane), and after incubating with P-body proteins under experimental conditions used for microscopy experiments for two hours (second lane), 24 hours (third lane), and 24 hours with 4 mM $MgCl_2$ added. RNA markers (nucleotides) labeled on the left.

Xrn1 does not degrade the defined RNAs used in these microscopy experiments because the RNAs lack 5’ monophosphorylation and because $MgCl_2$ is not used in the buffer. See Methods for further details.

Supplemental References

1. W. Xing, D. Muhlrad, R. Parker, M. K. Rosen, A quantitative inventory of yeast P body proteins reveals principles of composition and specificity. *Elife* **9** (2020).
2. U. Sheth, R. Parker, Targeting of aberrant mRNAs to cytoplasmic processing bodies. *Cell* **125**, 1095-1109 (2006).
3. O. Pellegrini, N. Mathy, C. Condon, L. Benard, In vitro assays of 5' to 3'-exoribonuclease activity. *Methods Enzymol* **448**, 167-183 (2008).
4. A. W. Johnson, R. D. Kolodner, Strand exchange protein 1 from Saccharomyces cerevisiae. A novel multifunctional protein that contains DNA strand exchange and exonuclease activities. *J Biol Chem* **266**, 14046-14054 (1991).
5. L. Zhou *et al.*, Crystal structure and biochemical analysis of the heptameric Lsm1-7 complex. *Cell Res* **24**, 497-500 (2014).
6. A. Chowdhury, J. Mukhopadhyay, S. Tharun, The decapping activator Lsm1p-7p-Pat1p complex has the intrinsic ability to distinguish between oligoadenylated and polyadenylated RNAs. *Rna* **13**, 998-1016 (2007).
7. S. H. Ling *et al.*, Crystal structure of human Edc3 and its functional implications. *Mol Cell Biol* **28**, 5965-5976 (2008).
8. H. Sharif, E. Conti, Architecture of the Lsm1-7-Pat1 complex: a conserved assembly in eukaryotic mRNA turnover. *Cell Rep* **5**, 283-291 (2013).
9. J. E. Braun *et al.*, A direct interaction between DCP1 and XRN1 couples mRNA decapping to 5' exonucleolytic degradation. *Nat Struct Mol Biol* **19**, 1324-1331 (2012).
10. C. Charenton *et al.*, A unique surface on Pat1 C-terminal domain directly interacts with Dcp2 decapping enzyme and Xrn1 5'-3' mRNA exonuclease in yeast. *Proc Natl Acad Sci U S A* **114**, E9493-E9501 (2017).
11. H. Sharif *et al.*, Structural analysis of the yeast Dhh1-Pat1 complex reveals how Dhh1 engages Pat1, Edc3 and RNA in mutually exclusive interactions. *Nucleic Acids Res* **41**, 8377-8390 (2013).
12. C. Charenton *et al.*, Structure of the active form of Dcp1-Dcp2 decapping enzyme bound to m(7)GDP and its Edc3 activator. *Nat Struct Mol Biol* **23**, 982-986 (2016).
13. M. Jinek, S. M. Coyle, J. A. Doudna, Coupled 5' nucleotide recognition and processivity in Xrn1-mediated mRNA decay. *Mol Cell* **41**, 600-608 (2011).
14. D. W. A. Buchan, D. T. Jones, The PSIPRED Protein Analysis Workbench: 20 years on. *Nucleic Acids Res* **47**, W402-W407 (2019).
15. K. Peng, P. Radivojac, S. Vucetic, A. K. Dunker, Z. Obradovic, Length-dependent prediction of protein intrinsic disorder. *BMC Bioinformatics* **7**, 208 (2006).
16. J. Jumper *et al.*, Highly accurate protein structure prediction with AlphaFold. *Nature* **596**, 583-589 (2021).
17. M. Varadi *et al.*, AlphaFold Protein Structure Database: massively expanding the structural coverage of protein-sequence space with high-accuracy models. *Nucleic Acids Res* **50**, D439-D444 (2022).
18. M. She *et al.*, Crystal structure of Dcp1p and its functional implications in mRNA decapping. *Nat Struct Mol Biol* **11**, 249-256 (2004).

19. M. She *et al.*, Structural basis of dcp2 recognition and activation by dcp1. *Mol Cell* **29**, 337-349 (2008).
20. J. Schindelin *et al.*, Fiji: an open-source platform for biological-image analysis. *Nat Methods* **9**, 676-682 (2012).
21. C. F. Mugler *et al.*, ATPase activity of the DEAD-box protein Dhh1 controls processing body formation. *Elife* **5** (2016).
22. D. Teixeira, R. Parker, Analysis of P-body assembly in Saccharomyces cerevisiae. *Mol Biol Cell* **18**, 2274-2287 (2007).
23. C. J. Decker, D. Teixeira, R. Parker, Edc3p and a glutamine/asparagine-rich domain of Lsm4p function in processing body assembly in Saccharomyces cerevisiae. *J Cell Biol* **179**, 437-449 (2007).
24. Z. Cheng, J. Coller, R. Parker, H. Song, Crystal structure and functional analysis of DEAD-box protein Dhh1p. *RNA* **11**, 1258-1270 (2005).
25. S. A. Fromm *et al.*, The structural basis of Edc3- and Scd6-mediated activation of the Dcp1:Dcp2 mRNA decapping complex. *EMBO J* **31**, 279-290 (2012).
26. T. Nissan, P. Rajyaguru, M. She, H. Song, R. Parker, Decapping activators in Saccharomyces cerevisiae act by multiple mechanisms. *Mol Cell* **39**, 773-783 (2010).
27. S. A. Fromm *et al.*, In vitro reconstitution of a cellular phase-transition process that involves the mRNA decapping machinery. *Angew Chem Int Ed Engl* **53**, 7354-7359 (2014).
28. S. Schutz, E. R. Noldeke, R. Sprangers, A synergistic network of interactions promotes the formation of in vitro processing bodies and protects mRNA against decapping. *Nucleic Acids Res* 10.1093/nar/gkx353 (2017).
29. Y. Lin, D. S. Protter, M. K. Rosen, R. Parker, Formation and Maturation of Phase-Separated Liquid Droplets by RNA-Binding Proteins. *Mol Cell* **60**, 208-219 (2015).
30. G. R. Pilkington, R. Parker, Pat1 contains distinct functional domains that promote P-body assembly and activation of decapping. *Mol Cell Biol* **28**, 1298-1312 (2008).
31. R. Sachdev *et al.*, Pat1 promotes processing body assembly by enhancing the phase separation of the DEAD-box ATPase Dhh1 and RNA. *Elife* **8** (2019).
32. M. Ernoult-Lange *et al.*, Multiple binding of repressed mRNAs by the P-body protein Rck/p54. *RNA* **18**, 1702-1715 (2012).
33. B. Ho, A. Baryshnikova, G. W. Brown, Unification of Protein Abundance Datasets Yields a Quantitative Saccharomyces cerevisiae Proteome. *Cell Syst* **6**, 192-205 e193 (2018).
34. M. Uchida *et al.*, Quantitative analysis of yeast internal architecture using soft X-ray tomography. *Yeast* **28**, 227-236 (2011).
35. G. W. Rogers, Jr., N. J. Richter, W. C. Merrick, Biochemical and kinetic characterization of the RNA helicase activity of eukaryotic initiation factor 4A. *J Biol Chem* **274**, 12236-12244 (1999).
36. D. Teixeira, U. Sheth, M. A. Valencia-Sanchez, M. Brengues, R. Parker, Processing bodies require RNA for assembly and contain nontranslating mRNAs. *RNA* **11**, 371-382 (2005).
37. S. Bellaousov, J. S. Reuter, M. G. Seetin, D. H. Mathews, RNAstructure: Web servers for RNA secondary structure prediction and analysis. *Nucleic Acids Res* **41**, W471-474 (2013).

38. M. Hondele *et al.*, DEAD-box ATPases are global regulators of phase-separated organelles. *Nature* **573**, 144-148 (2019).
39. S. C. Weber, Sequence-encoded material properties dictate the structure and function of nuclear bodies. *Curr Opin Cell Biol* **46**, 62-71 (2017).
40. C. Wang *et al.*, Correction: Context-dependent deposition and regulation of mRNAs in P-bodies. *Elife* **7** (2018).
41. A. Hubstenberger *et al.*, P-Body Purification Reveals the Condensation of Repressed mRNA Regulons. *Mol Cell* **68**, 144-157 e145 (2017).
42. M. Courel *et al.*, GC content shapes mRNA storage and decay in human cells. *Elife* **8** (2019).
43. U. Haupts, S. Maiti, P. Schwille, W. W. Webb, Dynamics of fluorescence fluctuations in green fluorescent protein observed by fluorescence correlation spectroscopy. *Proc Natl Acad Sci U S A* **95**, 13573-13578 (1998).
